# Standardization of Radiation Therapy to Inguinal and Pelvic Lymph Nodes in Locally Advanced Cancer of the Penis, as Defined by the International Penile Advanced Cancer Trial (InPACT)

**DOI:** 10.1016/j.ijrobp.2025.03.022

**Published:** 2025-09-01

**Authors:** Sian Cooper, Steve Nicholson, Juanita Crook, Nick Watkin, Curtis Pettaway, Jim Barber, Anita Mitra, Owain Woodley, Anthony Millin, Emma Hall, Angela Pathmanathan, Steven Penegar, Stephanie Burnett, Philippe Spiess, Elizabeth Miles, Karen Hoffman, Huiqi Yang, Alison C. Tree

**Affiliations:** a The Royal Marsden Hospital, London, United Kingdom; b The Institute of Cancer Research, London, United Kingdom; c Mid and South Essex NHS Foundation Trust, United Kingdom; d University of British Columbia, BCCancer Center for the Southern Interior, Kelowna, British Columbia; e St George's University Hospitals NHS Foundation Trust, United Kingdom; f The University of Texas MD Anderson Cancer Center, Texas; g Velindre University NHS Trust, Cardiff, United Kingdom; h University College London Hospitals NHS FT, London, United Kingdom; i National Radiotherapy Trials QA (RTTQA) Group, Northwood, United Kingdom; j Department of GU Oncology, Moffitt Cancer Center; Tampa, Florida; k East and North Hertfordshire NHS Trust, United Kingdom; l Department of Oncology, Addenbrooke's Hospital NHS Foundation Trust, Hills Rd, Cambridge, UK

## Abstract

**Purpose:**

InPACT addresses the optimal management of locally advanced penile cancer, aiming to prospectively evaluate the relative benefits and sequencing of surgery, chemotherapy, and chemoradiotherapy. At trial inception, radiation therapy protocols for this rare cancer lacked consistency and standardization, necessitating multicenter, international collaboration to develop comprehensive radiation therapy planning, delivery, and quality assurance guidelines.

**Methods and Materials:**

InPACT has 2 main aims; to establish the efficacy of neoadjuvant chemotherapy or chemoradiotherapy in patients with macroscopically-involved inguinal nodes. Second, to compare prophylactic pelvic lymph node dissection plus chemoradiation to the inguinal and pelvic fields versus chemoradiation alone in patients whose inguinal node histology predicts a high risk of occult pelvic node involvement. The primary outcome measure for the trial is survival time.

An international group was convened to achieve consensus on radiation therapy contouring, planning, dose, fractionation, and delivery for this rare cancer. These guidelines have been used throughout the conduct of the trial to date and form part of the radiation therapy quality assurance for each participating center.

**Results:**

International consensus radiation therapy guidelines were established, encompassing risk status assessment and indications for each treatment region based on radiological and pathologic risk status of nodal basins. Guidance provides a nodal contouring atlas, addresses prepubic fat coverage, and specifies dose fractionation for both neoadjuvant and adjuvant settings, including recommendations for macroscopic disease. Trial recruitment is ongoing. Oncological and toxicity outcomes will be reported in due course.

**Conclusions:**

The InPACT radiation therapy guidelines offer a step toward international consensus on contouring for inguino-pelvic radiation therapy in penile cancer.

## Introduction

Penile cancer is rare, with a limited body of evidence on which to base management decisions. Published data primarily includes case reports, retrospective studies, or single-institution experience. InPACT (the International Penile Advanced Cancer Trial) addresses the treatment of patients with penile squamous carcinoma (SCC) who have inguinal lymph node metastases (i.e., locally advanced disease). Five-year survival declines from 80% for patients with a single involved inguinal lymph node to between 0% and 12% for patients with pelvic node involvement.^1^, ^2^, ^3^ Conventional management for patients with clinically or radiologically overt groin nodes is therapeutic inguinal lymph node dissection (ILND). Chemoradiotherapy has traditionally been used in 2 scenarios: as palliative treatment for patients with locally advanced disease who are not fit for surgery; and as adjuvant therapy following groin dissection where disease has penetrated beyond the lymph node (extracapsular extension).^4^ This is distinct to other pelvic SCC such as vulva and anal canal where chemoradiotherapy is the standard of care recommended by NCCN guidelines.^5^ This has led to some centers adopting a similar approach for advanced penile cancer despite the lack of level-1 evidence.^6^^,^^7^

The study will determine the optimal sequencing of surgery, chemotherapy and chemoradiotherapy in the management of locally advanced penile cancer and define the relative benefits of these approaches in routine clinical practice.^8^ The trial will also address the question of whether there is any added benefit for prophylactic pelvic lymph node dissection (PLND) plus adjuvant chemoradiotherapy over adjuvant chemoradiotherapy alone in patients at high risk of occult pelvic lymph node involvement.

The purpose of this manuscript is to share the InPACT radiation therapy guidelines for this rare malignancy to provide guidance for clinicians managing a rare clinical scenario.

### International collaborative consensus

Prior to inception of InPACT, there was no consensus of radiation therapy sequencing, dose, fractionation, or volumes. Most clinicians infrequently treat penile cancer, due to its rarity. Under the auspices of the International Rare Cancers Initiative,^9^ the InPACT collaborative group developed a novel and practice-changing process of international consensus meetings among an expert peer group to standardize high quality radiation therapy delivery.^10^ The uniquely robust international quality assurance (QA) program, led by the National Radiotherapy Trials QA (RTTQA) group in the United Kingdom (UK) and the IROC Rhode Island QA Center in the United States (US) (File E1, File E2) established a contouring atlas, derived from joint review of clinician contours with detailed discussion of areas of discord. Benchmark cases were contoured by 6 international experts (UK and US) to ensure all were reporting and reviewing in the same manner. The benchmark cases were then used to credential clinicians at individual sites. The iterative expert review of contouring, and subsequent rigorous QA program to standardize radiation therapy delivery is both practice changing and evidence-building.

## Methods and Materials

### Study design

**Fig. 1:**
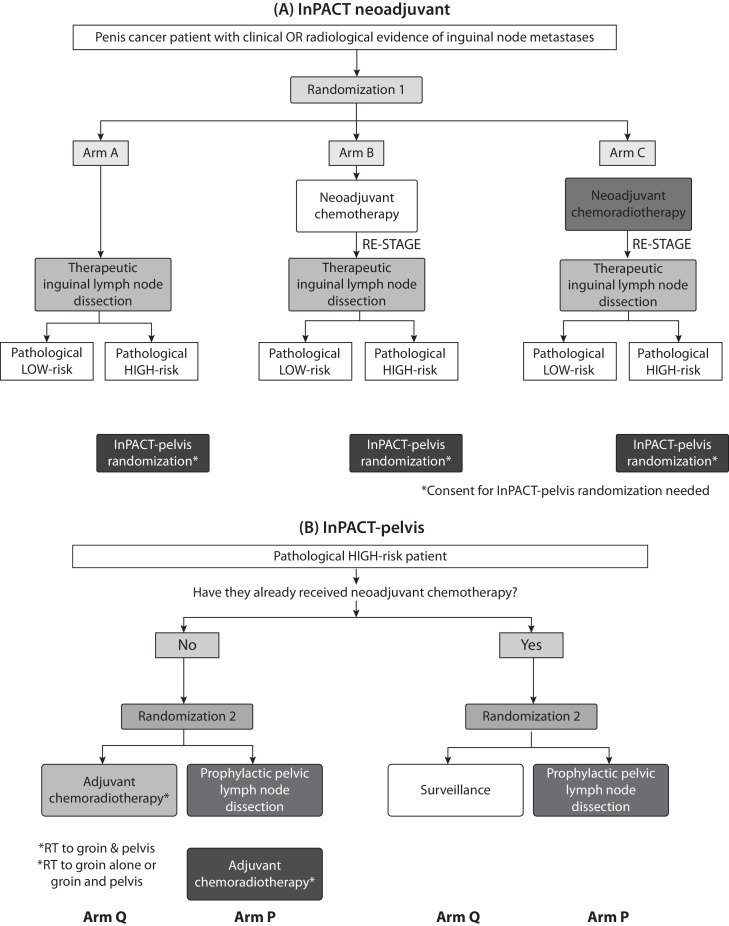
Trial schema for (A) InPACT neoadjuvant study; and (B) InPACT-pelvis study. Abbreviations: RT = radiation therapy.

InPACT is an international, randomized, multiarm, open-label, phase 3 trial with a Bayesian design. Trial governance is detailed in the Appendix E1. The trial design incorporates 2 randomizations: InPACT- neoadjuvant and InPACT-pelvis (trial schema shown in Fig. 1A, B). At registration, patients are stratified by disease burden (low, intermediate, or high) based on both physical examination and the computed tomography (CT) scan criteria developed by Graafland et al.^11^ Patients with clinical or radiological evidence of intermediate or high-risk inguinal node metastases are allocated to 1 of 3 initial treatments: standard surgery (ILND), neoadjuvant chemotherapy followed by standard surgery (ILND) or neoadjuvant chemoradiotherapy followed by standard surgery (ILND). After ILND, the patients are defined as being at low or high risk of recurrence based on histological interpretation of the ILND specimen. Pathological high risk is considered to be extranodal extension, bilateral nodal involvement, or 3 or more involved nodes. Patients at high risk of relapse are eligible for InPACT-pelvis, which randomizes between prophylactic PLND or no prophylactic PLND, plus chemoradiotherapy to the pelvis where not already given. Target radiation therapy volumes and doses, whether given as neoadjuvant or adjuvant chemoradiotherapy are detailed below.

The primary outcome measure for the trial is survival time. Secondary outcome measures are disease-specific and disease-free survival time, and freedom from locoregional recurrence and distant metastasis. Feasibility, toxicity, surgical complications, and quality of life will be assessed as secondary endpoints for all InPACT treatment arms.

### Participants and eligibility

Target recruitment is set at 200 participants, all of whom have histological confirmation of penile SCC with inguinal lymph node involvement that is suitable for radical treatment. Eligible patients have either radiological inguinal node involvement (InPACT neoadjuvant) or high-risk ILND specimen (InPACT-pelvis).^12^ Detailed eligibility is available in Protocol E1.

### Radiation therapy planning and delivery

Chemoradiotherapy should be delivered via intensity-modulated radiation therapy (IMRT), volumetric arc therapy (VMAT), or other rotational platform using 6 to 10-MV photons given in 25 fractions over 5 weeks. Concurrent chemotherapy is weekly cisplatin 40 mg/m^2^ (provided GFR > 45 mL/minute). Patient preparation, planning scan and general planning requirements are detailed in the appendix (Methods E1).

### Target volume definition

A nodal atlas for penile cancer was developed for the trial, based on experience of the vascular expansion method already published for other cancers.^13^^,^^14^ All clinical target volumes (CTVs) were qualitatively reviewed by a group of international experts. Areas of variation were discussed to form a consensus. For example, the presacral nodes are excluded, as this is an infrequent area of nodal drainage and relapse in penile cancer. Clinical knowledge of the frequency of in-transit metastases in the suprapubic region stipulated inclusion of the prepubic fat. Indications for each treatment region are detailed in Table 1 and Figure E1, based on the radiological and/or pathological status of each nodal basin. Each side of the pelvis should be considered separately, e.g., if the left groin is node negative, then left-sided pelvic treatment is not indicated. In contrast to locally advanced vulvar and anal SCC, where bilateral nodal CTVs are symmetrical,^15^^,^^16^ this approach allows for asymmetric nodal volumes. Comparison to the treatment of other pelvic SCC is detailed in the appendix (File E3).

**Table 1:** Indications for macroscopic disease/nodal groups dose selection. Each side of the pelvis to be considered separately

Neoadjuvant				
	Nodal disease/groups	Indication	Dose (in 25 fractions)	CTV
**Macroscopic nodal disease**	CTV_P	Macroscopic pelvic nodes + 5 mm	45 Gy	CTV_4500
	CTV_I	Macroscopic inguinal nodes + 5 mm		
**Lymph node group**	CTV_Common iliac	No pelvic nodal involvement	No treatment	N/A
		Any pelvic nodal involvement	45 Gy	CTV_4500
	**CTV_Pelvis_R** and/or **CTV_Pelvis_L** (consider right and left regions separately)	No inguinal disease (evidenced radiologically and histopathologically)	No treatment	N/A
		Presence of inguinal disease or if histologic staging unknown	45 Gy	CTV_4500
	**CTV_Inguinal_R** and/or **CTV_Inguinal_L** (consider right and left regions separately)	No inguinal disease (evidenced radiologically and histopathologically)	No treatment	N/A
		Presence of inguinal disease or if histological staging unknown	45 Gy	CTV_4500
	CTV_PrepubicFat	All patients	45 Gy	CTV_4500

Postoperative—no pelvic nodal dissection				
	Nodal disease/groups	Indication	Dose (in 25 fractions)	CTV
**Macroscopic nodal disease**	CTV_P	Boost macroscopic pelvic nodes + 5 mm	54 Gy	CTV_5400
	CTV_I	Boost macroscopic inguinal nodes + 5 mm	57 Gy	CTV_5700
**Lymph node group**	CTV_Common iliac	No pelvic nodal involvement	No treatment	N/A
		Pelvic nodal involvement (note CTV_P will be boosted to 54 Gy in presence of macroscopic disease at time of RT)	45 Gy	CTV_4500
	**CTV_Pelvis_R** and/or **CTV_Pelvis_L** (consider right and left regions separately)	No inguinal disease or Inguinal disease with low pathologic risk - No extracapsular spread - 1 positive node	No treatment	N/A
		Inguinal disease with high pathological risk: - Extracapsular spread and/or - 2 or more positive nodes and/or Presence of macroscopic inguinal disease at time of RT (i.e., lower pelvic boost)* Presence of macroscopic pelvic disease at time of RT	54 Gy	CTV_5400
	**CTV_Inguinal_R** and/or **CTV_Inguinal_L** (consider right and left regions separately)	No inguinal disease	No treatment	N/A
		Inguinal disease with low pathological risk† - No extracapsular spread - 1 positive node	45 Gy	CTV_4500
	**CTV_Inguinal_R** and/or **CTV_Inguinal_L** (consider right and left regions separately)	High pathological risk: Presence of inguinal disease with - Extracapsular spread - 2 or more positive nodes Presence of macroscopic inguinal disease at time of RT (note that CTV_I will be boosted to 57 Gy) Presence of macroscopic pelvic disease at time of RT (on the same side as the groin in question)	54 Gy	CTV_5400
	CTV_PrepubicFat	To be treated to 54 Gy in all patients (note that CTV_I will be boosted to 57Gy in the presence of macroscopic disease at time of RT)	54 Gy	CTV_5400

Postoperative—post pelvic nodal dissection				
	Nodal disease/groups	Indication	Dose (in 25 fractions)	CTV
**Macroscopic nodal disease**	CTV_P	Boost macroscopic pelvic nodes + 5 mm	54 Gy	CTV_5400
	CTV_I	Boost macroscopic inguinal nodes + 5 mm	57 Gy	CTV_5700
**Lymph node group**	CTV_CommonIliac	No pelvic nodal involvement (i.e., negative pelvic nodal dissection)	No treatment	N/A
		Pelvic nodal involvement (i.e., positive pelvic nodal dissection) (note CTV_P will be boosted to 54 Gy in presence of macroscopic disease at time of RT)	45 Gy	CTV_4500
	**CTV_Pelvis_R** and/or **CTV_Pelvis_L** (consider right and left regions separately)	No pelvic nodal involvement (i.e., negative pelvic nodal dissection)	No treatment	N/A
		Pelvic nodal involvement (i.e., positive pelvic nodal dissection) (note CTV_P will be treated to 54 Gy in presence of macroscopic disease at time of RT)	54 Gy	CTV_5400
	**CTV_Inguinal_R** and/or **CTV_Inguinal_L** (consider right and left regions separately)	No inguinal disease	No treatment	N/A
		Inguinal disease with low pathological risk - No extracapsular spread - 1 positive node	45 Gy	CTV_4500
		High pathological risk: Presence of inguinal disease with any of the following: - Extracapsular spread - 2 or more positive nodes Presence of macroscopic inguinal disease at time of RT (note that CTV_I will be boosted to 57 Gy) Presence of macroscopic pelvic disease at time of RT (on the ipsilateral side).	54 Gy	CTV_5400
	CTV_PrepubicFat	To be treated to 54 Gy in all patients (note that CTV_I will be boosted to 57 Gy in the presence of macroscopic disease at time of RT)	54 Gy	CTV_5400

*Abbreviations:* CTV = clinical target volume; RT = radiation therapy.

⁎ Lower pelvic boost: The whole of the ipsilateral external iliac CTV nodal chain is included in the CTV boost. The internal pudendal nodal chain is also included in this region due to the lymphatic drainage from the penis via the anterior branch of the internal iliac artery.

† If macroscopic pelvic nodal disease in the contralateral pelvis, the contralateral inguinal region should be treated to the same dose as the contralateral pelvis (i.e., 54 Gy) irrespective of the disease/risk status of the contralateral inguinal region.

### Primary tumor site

Both neoadjuvant and adjuvant nodal radiation therapy commence after complete surgical excision of the primary tumor with negative margins. If penile margins were involved, these areas should be defined as part of CTV prepubic fat.

### Guidance for contouring the nodal volumes

**Fig. 2:**
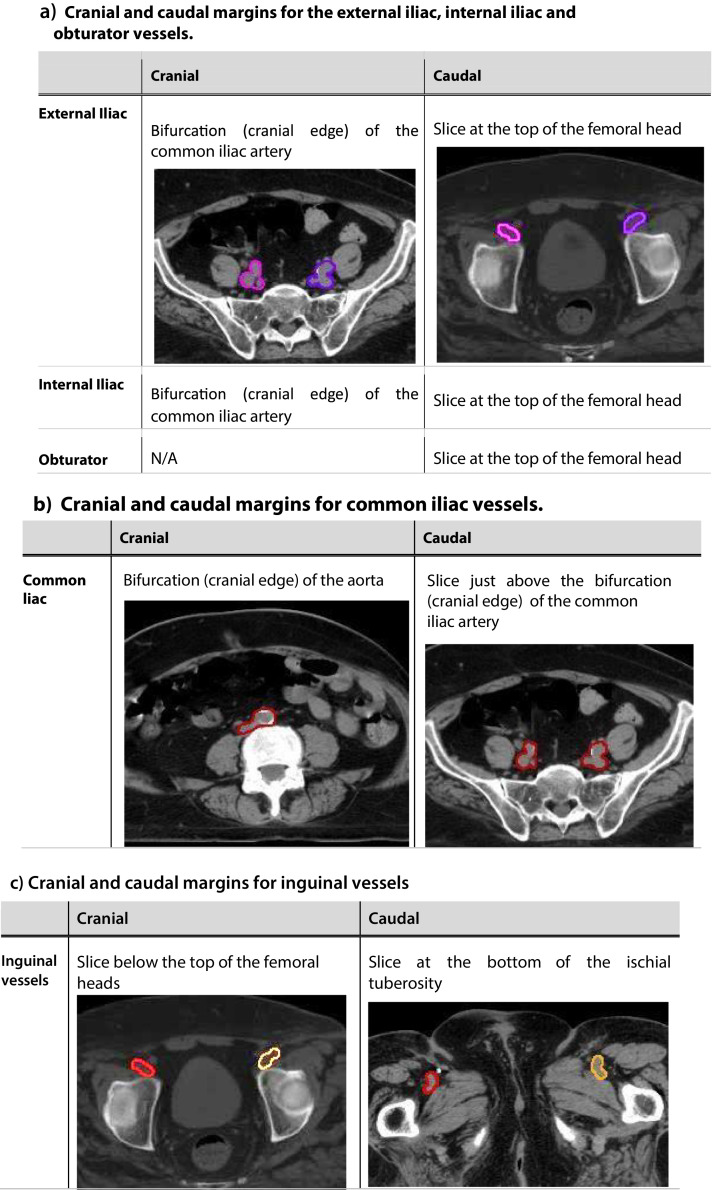

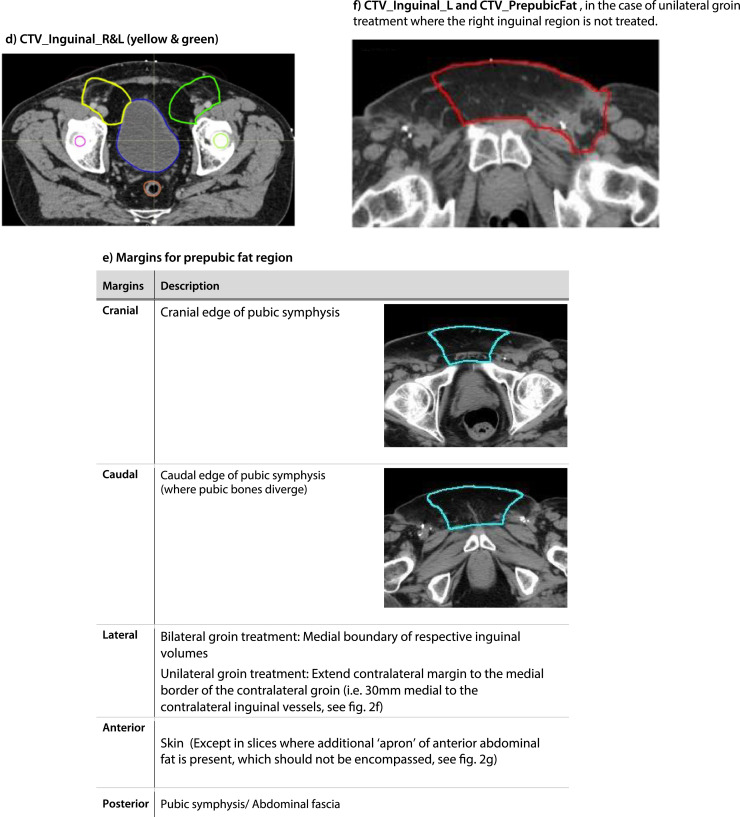

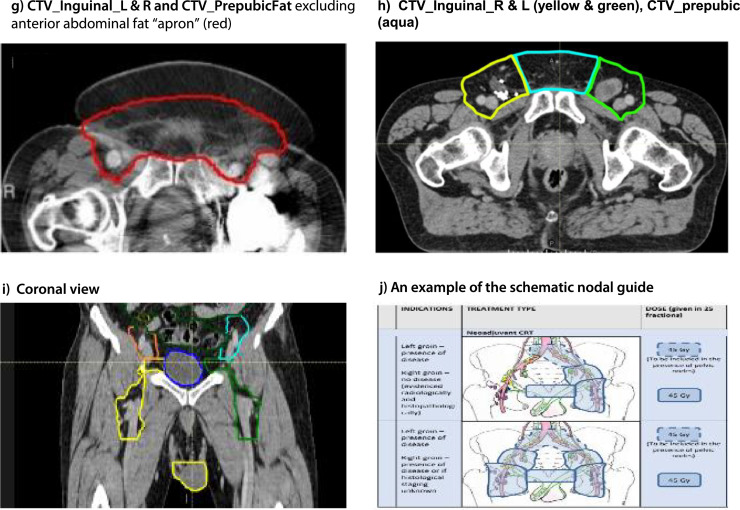
Nodal contouring guidance. (a) Cranial and caudal margins for the external iliac, internal iliac and obturator vessels (pink and purple). (b) Cranial and caudal margins for common iliac vessels (red). (c) Cranial and caudal margins for inguinal vessels (red and gold). (d) CTV_Inguinal_R and L (yellow and green). (e) Margins for prepubic fat region (aqua). (f) CTV_Inguinal_L and CTV_PrepubicFat (red). (g) CTV_Inguinal_L and R and CTV_PrepubicFat excluding anterior abdominal fat “apron” (red). (h) CTV_Inguinal_R and L (yellow and green), CTV_prepubic (aqua). (i) Coronal view. (j) An example of the schematic nodal guide. *Abbreviation:* CTV = clinical target volume.

The cranial margin must be at least 2 cm above the most superior aspect of macroscopic disease (if present) for all nodal group CTVs. Figure 2 denotes cranial and caudal extent of nodal volumes and the CTV prepubic fat boundaries.

#### CTV_Pelvis_R and CTV_Pelvis_L (External iliac, internal iliac, obturator nodal regions)

Two separate volumes are generated, CTV_Pelvis_R and CTV_Pelvis_L, encompassing the external iliac, internal iliac, and obturator vessels along each pelvic sidewall. Only the main internal iliac vessels are included in the volumes. The cranial and caudal margins are detailed in Figure 2. The vessels are expanded by a 7 mm margin in the lateral/medial/anterior/posterior axes. Muscle and bone are edited from the volume, as well as bowel and bladder. The anterolateral margin of the external iliac region is expanded by an additional 10 mm along the iliopsoas muscle to cover distal lateral external iliac nodes. The lateral internal iliac volume should reach the pelvic sidewall. In the superior aspect, the volume extends postero-laterally to include the region between the psoas and vertebral body. The external iliac region is joined to the internal iliac region using an 18 mm roller ball along lateral pelvis, excluding muscle if present. The caudal extent is the slice at the top of the femoral heads. At the level of the sciatic notch, ensure that the gluteal vessels running exterior to the bony pelvis are not included within the internal iliac contours. The presacral region is not included in the contours.

#### CTV_CommonIliac (common iliac nodal region)

The common iliac nodal regions are to be treated neoadjuvantly in the presence of pelvic disease. If there are suspicious or borderline pelvic nodes present in the postoperative setting, treatment of the common iliac nodal regions is at the judgment of the treating clinician. The common iliac vessels are outlined bilaterally as a single volume. This volume is expanded by a 7 mm margin in the lateral/medial/anterior/posterior axes, excluding bone and muscle, as well as bowel and sacral nerve roots. The volume should extend postero-laterally to include the region between the psoas and vertebral body.

#### CTV_Inguinal_R, CTV_Inguinal_L and CTV_PrepubicFat

The right and left main inguinal vessels are outlined as separate volumes, excluding smaller branching vessels. Each is expanded asymmetrically with a margin of 30 mm anteriorly and medially and a margin of 10 mm posteriorly, laterally, and inferiorly. There should be no expansion superiorly. The caudal border of CTV_Inguinal_R/L must be at least 2 cm below the most inferior aspect of any macroscopic disease (if present). Bone, muscle, and bladder are excluded from the CTV. In the presence of disease within the femoral canal, the CTV_Inguinal_R/L can be extended more medially to encompass the tissue at risk. The prepubic fat is outlined as a separate structure between the 2 inguinal volumes, to adjoin both inguinal volumes. For ipsilateral groin treatment, please note that the contralateral margin for prepubic fat should extend across the midline to the (untreated) contralateral groin. This margin should be at 30 mm medial to the contralateral inguinal vessels. The anterior border for prepubic fat should be extended to the skin. However, if an “apron” of anterior abdominal fat is present, this region should not be covered, and the anterior border of the prepubic fat should not extend to the skin for the affected slices. For all postoperative cases irrespective of the absence or presence of groin disease, extend the lateral and anterior boundary of the inguinal volumes to the inguinal fold/skin for the slices with the prepubic fat contours.

#### Other considerations

The inclusion of whole/part of seroma, surgical clips, or scar tissue outside the generated volumes is at the clinician's discretion.

### Dose guidance

#### Neoadjuvant

The CTV prepubic fat, and inguinal and pelvic lymph nodes in the presence of disease should be treated to 45 Gy in 25 fractions. Common iliac nodes should be treated if there are radiologically involved pelvic lymph nodes.

#### Adjuvant—no pelvic nodal dissection

A dose of 54 Gy in 25 fractions is delivered to all compartments with high-risk disease, plus prepubic fat. Low-risk (N1) contralateral groins receive 45 Gy. A boost to 57 Gy is given for residual macroscopic tumor (example shown in Fig. E2), and in the presence of pelvic nodal disease, the ipsilateral common iliac region is treated to 45 Gy. If there is macroscopic disease in the common iliac region, 45 Gy should be given with a boost to 57 Gy to the involved node.

#### Adjuvant—post pelvic nodal dissection

Where there is high-risk disease elsewhere in the pelvis, the low-risk groin should receive 45 Gy. If there was unexpected residual disease in the groin postoperatively, a boost to 57 Gy can be given. High-risk disease requires 54 Gy to the affected lymph node region and prepubic fat, but not the common iliac region unless there was pelvic nodal disease at PLND. If there is macroscopic disease in the common iliac region, 45 Gy should be given with a boost to 57 Gy.

#### Margins

All macroscopic nodal disease should be expanded 5 mm to CTV, edited to anatomical barriers. A margin is added in all direction to the CTV to generate the planning target volume (PTV) and should not be edited.^17^, ^18^, ^19^ It should be used for the reporting of doses to the target volumes. We propose the PTV margin should be 5 to 8 mm, but this margin will vary between institutions. The target volume definitions are delineated in Table 2.

**Table 2:** Overview of GTVs and CTVs in neoadjuvant and postoperative settings

Target volumes	GTV	CTV
Neoadjuvant	- GTV_I (if applicable) - GTV_P (if applicable)	- CTV_4500
Postoperative	- GTV_I (if applicable) - GTV_P (if applicable)	- CTV_4500 as applicable - CTV_5400 - CTV_5700 as applicable

Abbreviations: CTV = clinical target volume; GTV = gross tumor volume.

### Organs at risk delineation

The following are considered organs at risk (OARs): rectum, bowel bag, bladder, femoral head/neck, and scrotum; these should be outlined as solid structures by defining their outer wall (Methods E2).

### Radiation therapy planning

All patients should be CT planned. Radiation therapy should be delivered with either a forward or inverse planned IMRT/VMAT technique. Dose should be prescribed to the median of the respective dose target volumes. Dose objectives to the PTV should not be compromised to meet OAR constraints. The planning aim hierarchy is detailed in Methods E3. Prescription doses and target volume dose objectives are shown in Table 3. OAR dose–volume constraints were derived from PIVOTAL and INTERLACE trial protocols and are summarized in Table 4.^20^^,^^21^

**Table 3:** Prescription dose and target volume dose objectives

	PTV_5700 (boost)		PTV_5400		PTV_4500	
Prescription dose (Gy)	57		54		45	
Dose per fraction (Gy)	2.28		2.16		1.8	
	Mandatory	Optimal (PlanPTV)	Mandatory	Optimal (PlanPTV)	Mandatory	Optimal (PlanPTV)
D99%	≥90%	≥95%	≥ 90%	≥95%	≥ 90%	≥95%
D98%						
D95%	≥95%	≥98%	≥ 95%	≥98%	≥ 95%	≥98%
D50%	= 100% ± 1 Gy	= 100% ±1 Gy	= 100% ± 1 Gy	= 100% ±1 Gy	= 100% ± 1 Gy	= 100% ± 1 Gy
D5%	≤105%	≤105%	≤105%*	≤105%*	≤105%*	≤105%*
D2%	≤107%	≤107%	≤107%*	≤107%*	≤107%*	≤107%*

Abbreviations: PTV_5700 (BOOST) = Planning target volume to 57 Gy; PTV_5400 = Planning target volume to 54 Gy; PTV_4500 = Planning target volume to 45 Gy.

⁎ Not applicable if higher dose PTVs present, eg, PTV_5700 contained within PTV_5400 volume. Note as the PTV may extend outside of the skin, a planning PTV cropping to skin surface may be needed.

**Table 4:** Organs at risk dose objectives^20^^,^^21^

	Dose	Mandatory targets	Optimal targets
Rectum	V28 Gy	-	<80%
	V36 Gy	-	<65%
	V45 Gy	<60%	<50%
	V54 Gy	<50%	<35%
Bladder	V45 Gy	-	<50%
	V54 Gy	-	<25%
Bowelbag	V41 Gy	<158 cm^3^	<78 cm^3^
	V45 Gy	<110 cm^3^	<17 cm^3^
	V50 Gy	<28 cm^3^	<14 cm^3^
	V54 Gy	<6 cm^3^	<0.5 cm^3^
Femoralheadneck_R and Femoralheadneck_L	V50 Gy	<50%	

### Treatment delivery

Radiation therapy should be delivered 5 days a week until completion, avoiding breaks where at all possible (File E3). Treatment verification is recommended for at least the first 3 fractions to determine and correct for any systematic error in all 3 cardinal planes. This correction is applied on fraction 4, and a further image should be taken to confirm the move. Verification is then performed once weekly throughout the remaining treatment with a tolerance maximum of 5 mm. Planar MV or kV electronic portal imaging, volumetric imaging (cone beam CT), or a combination of these, can be used.^22^

### Radiation therapy protocol compliance program

The InPACT study is subject to a radiation therapy QA (RT QA) program which aims to standardize contouring, planning, and radiation therapy delivery. Benchmark cases contoured by international experts were used to credential clinicians at individual sites. The QA program for the study is coordinated by the RTTQA group in the UK, and the IROC Rhode Island QA Center in the US (File E2).

### Safety reporting

All serious adverse events have been monitored during the course of the trial and reviewed by the Independent Data Monitoring Committee. The trial expects to complete recruitment by May 2025.

## Conclusion

InPACT uses a pragmatic design to provide a randomized treatment approach for a rare cancer. The trial represents a significant step toward international protocol standardization and will provide high-level evidence with which to guide therapy for locally advanced penile cancer. The expert consensus contouring guideline, supported by a rigorous QA program, helps establish consistent radiation therapy practices for this rare cancer.

## Appendix Supplementary materials

InPACT protocol version 5.0 Image, application 1

mmc2 Image, application 2

## Footnotes

Supplementary material associated with this article can be found, in the online version, at doi:10.1016/j.ijrobp.2025.03.022.
