# Supplementary material for: Standardization of Radiation Therapy to Inguinal and Pelvic Lymph Nodes in Locally Advanced Cancer of the Penis, as Defined by the International Penile Advanced Cancer Trial (InPACT)
Source: Int J Radiat Oncol Biol Phys. 2025 Sep 1;123(1):171–82. doi: 10.1016/j.ijrobp.2025.03.022 (PMC12396133; doi:10.1016/j.ijrobp.2025.03.022)
Supplement: InPACT protocol version 5.0 [file mmc1.docx]

Contents

[eFile 1 Trial Governance 2](#_Toc184756835)

[eFile 2 RTQA 3](#_Toc184756836)

[eMethods 1 Patient preparation 4](#_Toc184756837)

[eFigure 1 Nodal Compartments for irradiation 5](#_Toc184756838)

[eFile 3 Comparison to other pelvic SCCs 10](#_Toc184756839)

[eMethods 2 Volumes 11](#_Toc184756840)

[eMethods 3 Planning Objectives 11](#_Toc184756841)

[eFile 4 Treatment delivery 12](#_Toc184756842)

[eMethods 4 Follow up protocol 13](#_Toc184756843)

[eMethods 5 Patient and public involvement 14](#_Toc184756844)

## eFile 1 Trial Governance

InPACT is sponsored by The Institute of Cancer Research (UK) and National Cancer Institute (NCI) (US). Trial coordination is managed by the Clinical Trials and Statistics Unit at the Institute of Cancer Research (ICR-CTSU) (UK) collaborating with ECOG-ACRIN Cancer Research Group (US). Funded by Cancer Research UK (CRUK/13/005), supported by Stand Up To Cancer (ref: 16050)

Support is acknowledged from the National Institute for Health Research Cancer Research Network and NHS funding to the NIHR Biomedical Research Centre at the Royal Marsden NHS Foundation Trust and The Institute of Cancer Research, London and the National Cancer Institute of the United States. The National Radiotherapy Trials Quality Assurance (RTTQA) Group is funded by the National Institute for Health and Care Research (NIHR).

InPACT (ECOG-ACRIN EA8134) was supported in the United States by the National Cancer Institute of the National Institutes of Health under award numbers: U10CA180820 and U10CA180858. The content is solely the responsibility of the authors and does not necessarily represent the official views of the National Institutes of Health

The trial adheres to the Research Governance Framework for Health and Social Care and principles of Good Clinical Practice. The trial is approved in the UK by the London Riverside ethics committee (ref: 16/LO/1355) and elsewhere by the relevant institutional review boards.

Trial data are obtained, managed, stored, shared, and archived according to ICR-CTSU standard operating procedures to ensure the enduring quality, integrity, and utility of the data. The trial is overseen by the trial management group and independent trial steering and data monitoring committees who provide expert oversight who safeguard the interests of trial participants and review emerging safety and efficacy data.

The trial is registered on the clinicaltrials.gov database (NCT02305654) and is included in the National Institute for Health and Care Research (NIHR) Clinical Research Network portfolio.

## eFile 2 RTQA

The details of the programme can be found on the RTTQA website, [www.rttrialsqa.org.uk](http://www.rttrialsqa.org.uk). Coordinators of radiotherapy QA in North America are https://irochouston.mdanderson.org/rpc/credentialing/credentialing.htm.

## eMethods 1 Patient preparation

Patients should be immobilised supine with a minimum of ankle and/or knee supports, and arms comfortably out of the radiotherapy treatment field. Local bladder and bowel preparation protocols should be followed. Bladder should be comfortably full and reproducible, ideally with a volume >150mls and <300mls. Bowel preparation aims to achieve a reproducible empty rectum, ideally with a rectal AP diameter of $\leq$4cm.

Scan in supine position from top of L5 (top of L2 if requiring common iliac treatment) to the lower limit of any surgical scar or mid-thigh, whichever is lower. Slice thickness should be 3mm or less. Intravenous contrast should be used unless clinically contraindicated.

Target volume definition: Volumes will be defined according to the International Commission on Radiation Units and Measurements (ICRU) report 50, supplement report ICRU 62, and ICRU 83^11–13^

## eFigure 1 Nodal Compartments for irradiation


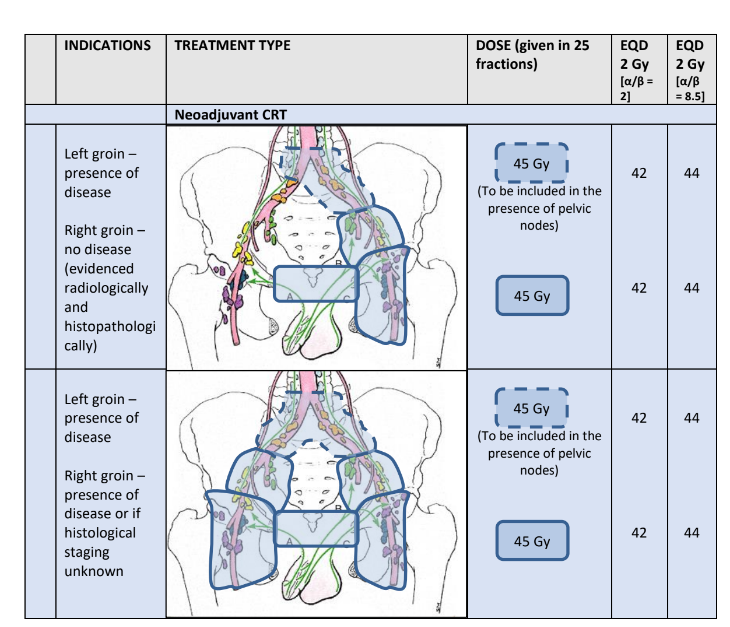


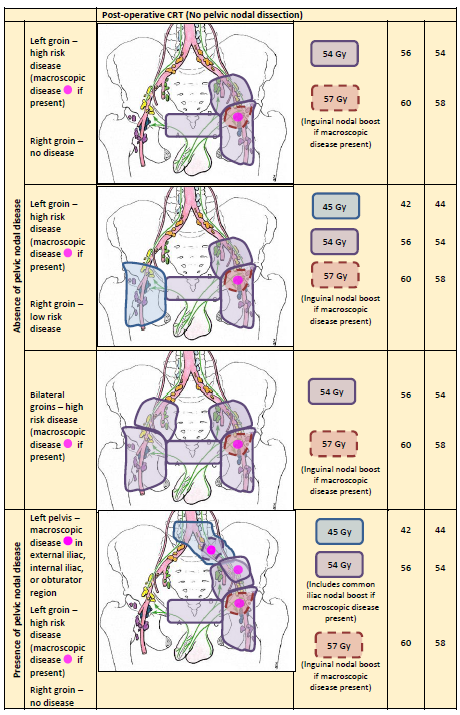


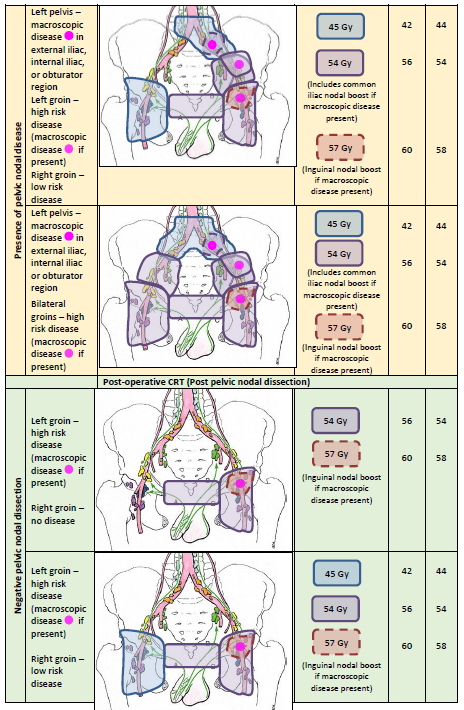


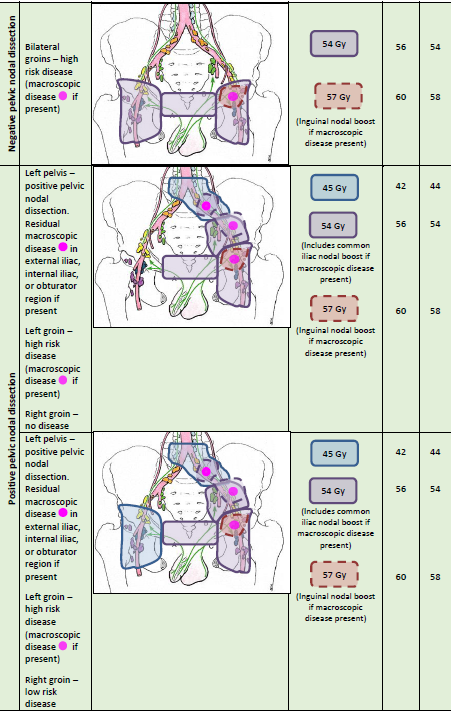


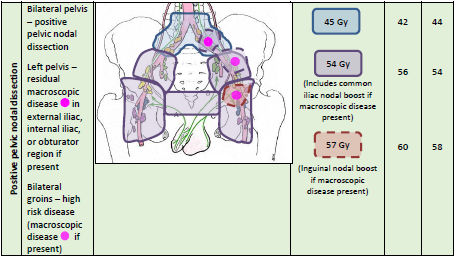


## eFigure 2 Gross residual volume contours

An example of contours for involved inguinal lymph nodes. The case shows post-operative imaging with residual right inguinal macroscopic disease, in addition to bilateral external iliac and right common iliac nodal involvement with no evidence of distant metastases. The patient received adjuvant chemoradiotherapy to the groin and pelvis. The residual macroscopic nodal disease in the right groin was defined as GTV_I, and the nodal disease in the pelvis was contoured separately as GTV_P. CTV_I and CTV_P were created from GTV_I and GTV_P respectively using a 5mm isotropic expansion margin, which were edited from bone and muscle.


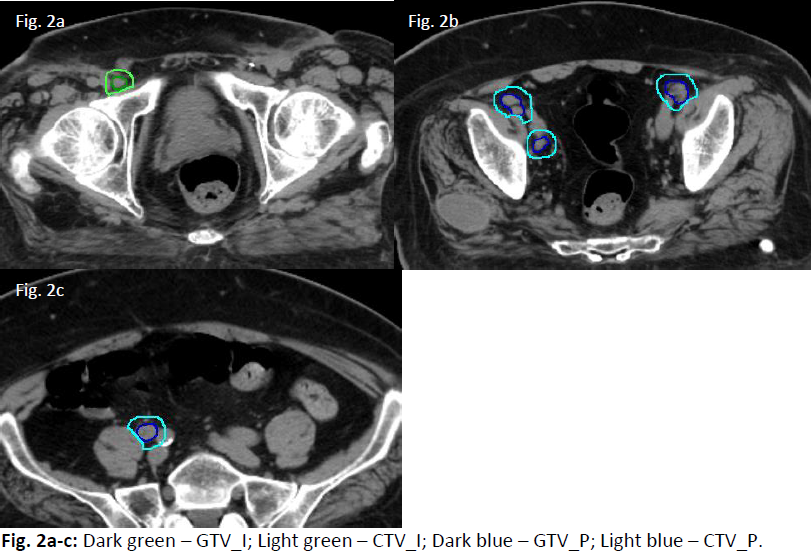


## eFile 3 Comparison to other pelvic SCCs

Inguinal radiotherapy is given in anal, vulvar and penile malignancy. All three disease sites share an identical superior CTV border, defined where the external iliac artery exits the pelvis to become the femoral artery. For the inferior border, while vulvar cancer extends 2cm below the saphenofemoral junction^15,20^, both anal and penile cancer protocols use anatomical landmarks - the lesser trochanter and ischial tuberosity respectively, which notably appear at the same axial CT level^21^. Volume expansions differ. Penile cancer employs asymmetric margins of 10-30mm, while vulvar cancer requires larger expansions between 22-35mm. Anal cancer defines specific anatomical boundaries using the medial edge of sartorius or iliopsoas laterally, and the pectineus, adductor longus and iliopsoas posteriorly, approximately 5mm from the skin surface. Dose prescriptions also vary by disease site. Vulvar cancer typically receives 45-50.4Gy in 25-28 fractions to elective volumes, with involved nodal beds boosted up to 60 Gy. Elective nodes in anal cancer receive 45Gy in 25 fractions, with 50.4-54Gy delivered to involved nodes. Uniquely in anal cancer, the radiotherapy field encompasses the presacral space and mesorectum, and inguinal nodes are always included for locally advanced disease^22^.

## eMethods 2 Volumes

If the PTV extends beyond skin contour, sometimes a planning PTV may be used. The organs at risk are: rectum, bowel bag, bladder, femoral head neck and scrotum, will be outlined as solid structures by defining their outer wall. The rectum is outlined to include the full circumference (maximum ideal diameter 4cm A-P) and rectal contents from the ano-rectal junction to the recto-sigmoid junction. The small and large bowel (including sigmoid colon) will be outlined as a single structure labelled ‘bowelbag’. The cranial extent of ‘bowelbag’ outlining should be 2 cm beyond the superior extent of the PTV.

Both the femoral heads and necks are outlined to the inferior border of the lesser trochanter. The scrotum and testicles should be outlined as a single structure, to the cranial edge of the testicles.

## eMethods 3 Planning Objectives

Prioritisation of planning objectives:

- Mandatory PTV coverage
- Mandatory OAR objectives
- Optimal PTV coverage
- Optimal OAR objectives
- Other non-specified normal tissue objectives

All patients are CT planned. Radiotherapy is to be delivered with either a forward or inverse planned IMRT technique. Rotational arc therapies are permitted (Rapid Arc™, VMAT™ and Tomotherapy™). When using static IMRT, a 5 to 7 field technique is recommended to obtain uniform coverage of the PTV and satisfy the dose constraints to the organs at risk (OARs). Dose distributions should be calculated and corrected for inhomogeneities. Where the PTV extends beyond the skin, if the optimiser struggles to deliver a solution, the volume can be withdrawn from the skin surface to achieve an optimal plan.

## eFile 4 Treatment delivery

Neoadjuvant chemoradiotherapy should start within 4 weeks of randomisation, and within 8 weeks post-surgery in the adjuvant arm. Treatment breaks should only be permissible for severe acute toxicity or intercurrent illness. The same patient preparation instructions used at planning CT should be implemented prior to each fraction delivered for verification. In the event of missed fractions due to logistic reasons, compensation for the missed fraction can be achieved by either treating at a weekend or by hyperfractionating, that is, undertaking two fractions a day (ideally on a Friday) with a minimum 6-hour gap between treatments.

## eMethods 4 Follow up protocol

Example of study follow up: InPACT-neoadjuvant – Arm C (neoadjuvant chemoradiotherapy + ILND) only as per EAU guidelines^18^. Cross sectional imaging preferred method is CT chest, abdomen, and pelvis. Alternatively, MRI pelvis and abdomen and CT chest.

|  | **Neoadjuvant chemoradiotherapy** | **Restage** | **ILDN surgery** | **Follow-up (months)** | | | | | | | | | | | | |
| --- | --- | --- | --- | --- | --- | --- | --- | --- | --- | --- | --- | --- | --- | --- | --- | --- |
| **Time from start of treatment** | **0**  **Start within 4 weeks of trial entry** | **6 weeks** | **12 weeks** | **6** | **9** | **12** | **15** | **18** | **21** | **24** | **30** | **36** | **42** | **48** | **54** | **60** |
| Bloods: haematology & Biochemistry | X | X | X |  |  |  |  |  |  |  |  |  |  |  |  |  |
| GFR | X |  | X |  |  |  |  |  |  |  |  |  |  |  |  |  |
| Cross-sectional imaging | X | X |  | X |  | X |  | X |  | X |  |  |  |  |  |  |
| Tumour Assessment RECIST v1.1 |  | X |  |  |  |  |  |  |  |  |  |  |  |  |  |  |
| CTCAEv4.0 toxicity assessment | X |  | X |  | X |  | X |  | X |  | X | X | X | X | X | X |
| Quality of life questionnaire |  | X | X |  |  | X |  | X |  | X |  | X |  |  |  |  |
| RTOG |  |  | X |  | X |  | X |  | X |  | X | X | X | X | X | X |

## eMethods 5 Patient and public involvement

PPI input has been actively sought from the early development of the InPACT study. There is PPI representation on the trial oversight committees. Members guide trial design, contribute to patient documentation, and raise awareness of the trial amongst relevant disease advocacy groups. All InPACT PPI representatives have been supportive of capitalising on international efforts to get the best possible results from the trial and will help disseminate and communicate the research findings for patient benefit.
