# Supplementary material for: Standardization of Radiation Therapy to Inguinal and Pelvic Lymph Nodes in Locally Advanced Cancer of the Penis, as Defined by the International Penile Advanced Cancer Trial (InPACT)
Source: Int J Radiat Oncol Biol Phys. 2025 Sep 1;123(1):171–82. doi: 10.1016/j.ijrobp.2025.03.022 (PMC12396133; doi:10.1016/j.ijrobp.2025.03.022)
Supplement: mmc2 [file mmc2.pdf]

InPACT

International Penile Advanced Cancer Trial (International Rare Cancers Initiative study)

Version 5.0
Dated: 16th February 2023

Study Chair:	Dr Steve Nicholson
Sponsors:	The Institute of Cancer Research (UK)<br>National Cancer Institute (US)
Approval:	UK: Cancer Research UK: Clinical Trials Awards & Advisory Committee (CTAAC)<br>US: National Cancer Institute, Cancer Therapy Evaluation Programme (NCI CTEP)
Funders:	Cancer Research UK – Stand Up to Cancer
Coordinating Trials Unit:	ICR Clinical Trials and Statistics Unit (ICR-CTSU)<br>The Institute of Cancer Research
Collaborating Groups/Trials Units:	ECOG-ACRIN Cancer Research Group

EudraCT Number:	2015-001199-23
Main REC Reference Number:	16/LO/1355
ICR-CTSU Protocol Number:	ICR CTSU/2014/10048
ISRCTN:	13580965
ClinicalTrials.gov Identifier	NCT02305654
CRUK Reference Number:	CRUK/13/005
IRCI Reference Number:	IRCI004
CCR Number	4241

This protocol is a controlled document and should not be copied, distributed or reproduced without the written permission of the ICR-CTSU

ADMINISTRATION

Clinical & Scientific Coordination

Steve Nicholson (Study Chair)
c/o ICR Clinical Trials & Statistics Unit (ICR-CTSU)
Division of Clinical Studies
The Institute of Cancer Research
Sir Richard Doll Building
15 Cotswold Road
Sutton Surrey SM2 5NG
Phone (0044) 7808480411
Fax (0044) 2087707876
Email: wondersn@doctors.org.uk

Curtis Pettaway (US Co-Chair)
Professor of Urology
University of Texas
MD Anderson Cancer Centre
1515 Holcombe Blvd. Unit 1373
Houston, Texas 77030
Phone (713)792-3250
Fax (713) 794-4824
Email: cpettawa@mdanderson.org

Nick Watkin (National Coordinator for the UK)
St George's Healthcare NHS Trust
Blackshaw Road
Tooting, London, SW17 0QT

Surgical lead:
Nick Watkin (Surgical subgroup co-chair) — St. Georges Hospital, London
Philippe Spiess (North American Oversite Committee chair) — Moffitt Cancer Center, Florida

Chemotherapy lead:
Lance Pagliaro (Chemotherapy subgroup co-chair) — Mayo Clinic, Minnesota

Chemoradiotherapy lead:
Jim Barber (ChemoRT subgroup chair) — Velindre Hospital, Cardiff

Imaging lead:
Mark Rosen (Imaging subgroup chair) — Imaging and Radiation Oncology Core (IROC), Philadelphia

Pathology lead:
Jon Oxley (UK lead) — North Bristol NHS Trust
Pheroze Tamboli (US lead) — MD Anderson Cancer Centre, Texas

Statistical advisor:
Lucinda Billingham — University of Birmingham

Quality of Life advisor:
Peter Branney — Leeds Metropolitan University

Coordinating Trials Unit Scientific lead:
Emma Hall — The Institute Of Cancer Research

Coordinating Trials Unit Methodology lead:
Christina Yap — The Institute Of Cancer Research

Coordinating Trials Unit Clinical Trials Programme Manager:
Stephanie Burnett — The Institute Of Cancer Research

Trial Coordination

ICR-CTSU (a UKCRC registered NCRI cancer clinical trials unit) is responsible for the central coordination of the trial on behalf of the Sponsors.

ICR Clinical Trials & Statistics Unit (ICR-CTSU),
Division of Clinical Studies,
The Institute of Cancer Research,
London, SM2 5NG
United Kingdom
Email: InPACT-icrctsu@icr.ac.uk

ICR-CTSU Statistician: Abdullahi Omar
Email: InPACT-icrctsu@icr.ac.uk Tel: 020 8722 4535

InPACT Trial Manager: Steven Penegar
Email: InPACT-icrctsu@icr.ac.uk Tel: 020 8722 4238

In the UK: Any questions relating to this protocol should be addressed in the first instance to the InPACT Trial Manager at ICR-CTSU:

Email: InPACT-icrctsu@icr.ac.uk General enquiries: +44 (0)208 722 4238

In the US and South America: InPACT runs under a parallel protocol (EA8134) with a country-specific appendix and is sponsored by the NCI. Any questions relating to this protocol should be addressed in the first instance to the ECOG-ACRIN Operations Office in Boston. Please call during normal business hours for protocol related questions and ask for the DA for EA8134.
Email: ea8134team@jimmy.harvard.edu General enquiries: (857)504-2900.

In mainland Europe: Any questions relating to this protocol should be addressed in the first instance to the InPACT Trial Manager at ICR-CTSU:

Email: InPACT-icrctsu@icr.ac.uk General enquiries: +44 (0)208 722 4238 Fax: 0208 770 7876

Protocol Authorised by:

Name & Role	Signature	Date
Steve Nicholson<br>Study Chair		19/02/2023
Curtis Pettaway<br>US Study co-Chair		21/02/2023
Nick Watkin<br>National Coordinator for the UK		17/02/2023

This protocol describes the InPACT trial and provides information about procedures for entering participants into this trial. The protocol should not be used as a guide for the treatment of patients outside of this trial.

Every care was taken in the preparation of this protocol, but corrections or amendments may be necessary. Protocol amendments will be circulated to participating sites as they occur.

UK sites entering patients for the first time are advised to contact ICR-CTSU to confirm they have the most recent version.

US sites entering patients for the first time are advised to contact the ECOG-ACRIN Operations Office to confirm they have the most recent version.

CONTENTS

InPACT TRIAL SUMMARY 1
TRIAL SCHEMA 2
1. INTRODUCTION 3
1.1. Background 3
1.2. Study rationale 3
1.2.1. Rationale for investigation of the role of chemoradiotherapy 3
1.2.2. Rationale for investigation of the role of neoadjuvant chemo(radio)therapy 4
1.2.3. Rationale for investigation of the role of prophylactic pelvic lymph node dissection (PLND) 4
1.2.4. Role of HPV in penis cancer 4
1.3. Risks and benefits of treatment regimens in InPACT 4
1.3.1. Standard surgery 4
1.3.2. Neoadjuvant chemotherapy 4
1.3.3. Neoadjuvant chemoradiotherapy 5
1.3.4. Adjuvant chemoradiotherapy following ILND 6
1.3.5. Prophylactic pelvic lymph node dissection (PLND) and chemoradiotherapy following ILND 6
2. TRIAL OBJECTIVES 6
2.1. Primary objective 6
2.2. Secondary objectives 7
2.3. Exploratory objectives 7
3. TRIAL DESIGN 7
4. STUDY OUTCOME MEASURES 8
4.1. Primary outcome measure 8
4.2. Secondary outcome measures 8
5. PATIENT SELECTION AND ELIGIBILITY 8
5.1. Number of participants 8
5.2. Source of participants 8
5.3. Inclusion criteria 9
5.4. Exclusion criteria 10
5.5. InPACT-neoadjuvant additional eligibility criteria 11
5.5.1. Nodal disease burden 11
5.5.2. Glomerular filtration rate (GFR) 11
5.5.3. Radiological evidence of macroscopic pelvic node involvement 12
5.5.4. ROLE OF PET-CT scanning in determining eligibility for InPACT-neoadjuvant 13
5.5.5. Summary of InPACT-neoadjuvant randomisation/treatment options 13
5.6. InPACT-pelvis (randomisation 2) additional eligibility criteria 14
5.7. Life style guidelines 14
6. SCREENING 15
6.1. Screening log 15
6.2. Procedure for obtaining informed consent 15
6.3. Participation in other clinical trials 16
7. TRIAL ENTRY/RANDOMISATION 16
7.1. InPACT-neoadjuvant – trial entry or randomisation 16
7.2. InPACT-pelvis – randomisation 17
8. TRIAL ASSESSMENTS 17
8.1. Baseline assessments 17
8.2. On-treatment assessments – InPACT-neoadjuvant 18
8.2.1. Arm A - standard surgery (ILND) 18
8.2.2. Arm B – neoadjuvant chemotherapy + ILND 18
8.2.3. Arm C – neoadjuvant chemoradiotherapy + ILND 19
8.3. On treatment assessments – InPACT-pelvis 19
8.3.1. Arm P – prophylactic PLND 20

8.3.2. Arm Q – no prophylactic PLND 20
8.4. Adjuvant chemoradiotherapy for patients not proceeding to InPACT-pelvis 20
8.5. Follow-up 21
8.6. Procedure at disease progression/recurrence 22
8.7. Discontinuation from treatment or follow-up 22
8.8. Pathology review 22
8.8.1. Primary tumour and lymph node review (prior to randomisation into InPACT) and response review22
8.8.2. Post-operative risk classification 23
8.8.3. Pathology quality assurance (QA) 23
8.9. Radiology review of staging scans 23
8.10. Schedules of assessments 24
9. STUDY TREATMENT 34
9.1. Therapeutic options 34
9.2. Inguinal lymph node dissection (ILND) 35
9.3. Neoadjuvant chemotherapy 35
9.3.1. TIP chemotherapy regimen 36
9.3.2. Supportive medication 37
9.3.3. Dose banding and rounding 38
9.3.4. Dose capping 38
9.3.5 Chemotherapy dose modifications/dose delays 38
9.3.6 Toxicity grading and criteria to discontinue chemotherapy 39
9.4. Chemoradiotherapy 40
9.4.1. Target volume 40
9.4.2. Dose 40
9.4.3. Duration of Treatment 41
9.5. Pelvic lymph node dissection (PLND) 41
9.5.1. Prophylactic Pelvic lymph node dissection (prophylactic PLND) 41
9.5.2. Pelvic lymph node dissection (therapeutic PLND) 41
10. CONCOMITANT THERAPY 41
11. RADIOTHERAPY QUALITY ASSURANCE (RT QA) 42
12. SURGICAL QUALITY ASSURANCE 42
13. PATIENT CARDS 42
14. PHARMACY RESPONSIBILITIES AND DRUG ACCOUNTABILITY 43
15. PHARMACOVIGILANCE 43
15.1. Definitions 43
15.1.1. Adverse event (AE) 43
15.1.2. Serious adverse event (SAE) 43
15.1.3. Serious adverse reaction (SAR) 44
15.1.4. Suspected unexpected serious adverse reaction (SUSAR) 44
15.1.5. Related Unexpected Serious Adverse Event 44
15.2. UK reporting adverse events to ICR-CTSU 44
15.3. UK reporting of serious adverse events to ICR-CTSU 45
15.4. UK review of serious adverse events 45
15.5. UK expedited reporting of SUSARs 45
15.6. UK expedited reporting of Related Unexpected SAEs 46
15.7. UK follow up of serious adverse events 46
15.8. UK annual reporting of serious adverse reactions 46
15.9. Reporting pregnancies 46
16. STATISTICAL CONSIDERATIONS 49
16.1. Trial design 49
16.2. Treatment Allocation 50

16.2.1. InPACT-neoadjuvant – Randomisation 1 50
16.2.2. InPACT-pelvis – Randomisation 2 51
16.3. Outcome measure definitions 51
16.3.1. Primary outcome measure 51
16.3.2. Secondary outcome measures 51
16.4. Statistical analysis plan 52
16.5. Interim analyses and stopping rules 54
17. TRIAL OVERSIGHT 55
17.1. Executive committee 55
17.2. Trial Management Group (TMG) 55
17.3. Trial Steering Committee (TSC) 56
17.4. Independent Data Monitoring Committee (IDMC) 56
18. RESEARCH GOVERNANCE 56
18.1. Sponsor responsibilities 56
18.2. Participating site responsibilities 56
19. TRIAL ADMINISTRATION AND LOGISTICS 56
19.1. Site activation 56
19.2. Data acquisition 57
19.3. Central data monitoring 57
19.4. On-site monitoring 57
19.5. Completion of the study and definition of study end date 57
19.6. Archiving 57
20. PATIENT PROTECTION AND ETHICAL CONSIDERATIONS 57
20.1. Trial approvals 57
20.2. Trial conduct 58
20.3. Informed consent 58
20.4. Patient confidentiality 58
20.5. Data Protection 58
20.6. Liability 58
21. FINANCIAL MATTERS 58
22. PUBLICATION POLICY 59
23. ASSOCIATED STUDIES 59
23.1. Translational study 59
23.2. Quality of Life (QL) study 61
24. REFERENCES 62
APPENDIX 1 - PRE HYDRATION AND POST HYDRATION EXAMPLES FOR TIP REGIMEN. 64
APPENDIX 2 - ECOG PERFORMANCE STATUS 65
APPENDIX 3 - CREATININE CLEARANCE CALCULATION 66
APPENDIX 4 - QUALITY OF LIFE STUDY 67
APPENDIX 5 – Chemotherapy dose levels and use of GCSF 71
APPENDIX 6 - Modifed Clavien- Dindo Classification* 75
APPENDIX 7 - Response Evaluation Criteria in Solid Tumours (RECIST v 1.1) Quick Reference 77
APPENDIX 8 – Expected serious adverse events for radiotherapy or surgery alone 79
Glossary 80

InPACT TRIAL SUMMARY

PROTOCOL TITLE	InPACT (International Penile Advanced Cancer Trial)
PRIMARY STUDY OBJECTIVES	In the management of patients with locally advanced squamous carcinoma of the penis:<br>1. (a) Is there a role for neoadjuvant therapy and, if so, (b) does chemotherapy or chemoradiotherapy produce superior outcomes (either for survival endpoints or for morbidity/quality of life endpoints)?<br>2. Does prophylactic pelvic lymph node dissection (PLND) improve survival in patients at high risk of recurrence following ILND?
STUDY DESIGN	International phase III trial with a Bayesian design incorporating two sequential randomisations.
TRIAL POPULATION	Patients with squamous carcinoma of the penis who have inguinal lymph node metastases (i.e. locally-advanced disease).
RECRUITMENT TARGET	200 patients.
TREATMENT REGIMEN	Randomisation 1 (InPACT-neoadjuvant): Dependent on disease burden strata, patients are allocated to one of three initial treatment arms:<br>A. standard surgery;<br>B. neoadjuvant chemotherapy followed by standard surgery; or<br>C. neoadjuvant chemoradiotherapy followed by standard surgery.<br><br>Randomisation 2 (InPACT-pelvis): Patients at high risk of relapse based on histological interpretation of the inguinal lymph node dissection (ILND) (standard surgery) specimen are randomised to either:<br>P. prophylactic pelvic lymph node dissection; or<br>Q. no prophylactic pelvic lymph node (PLND).<br><br>Patients receiving initial treatment of surgery alone or neoadjuvant chemotherapy followed by surgery in whom high-risk disease is resected will receive adjuvant chemoradiotherapy.
PRIMARY ENDPOINT	Survival time.
KEY SECONDARY ENDPOINTS	Disease specific and disease free survival time;<br>Toxicity;<br>Occurrence of surgical complication;<br>Feasibility of pathological nodal assessment after chemotherapy;<br>Quality of life.
QUALITY OF LIFE (QL) SUB-STUDY	For centres participating in the QL sub-study, patients will receive QL booklets at the following intervals; baseline (prior to randomisation), post-neoadjuvant treatment (where applicable), post-ILND, 12 months from the start of treatment, and then at 18, 24 and 36 months. Further details are given in section 23.2 and in appendix 4.
FOLLOW UP	All patients will undergo clinical review in accordance with the guidelines of the European Association of Urology (EAU), namely every 3 months (from the first day of trial treatment) for years 1 and 2, then every 6 months for years 3, 4 and 5.

TRIAL SCHEMA

InPACT-neoadjuvant

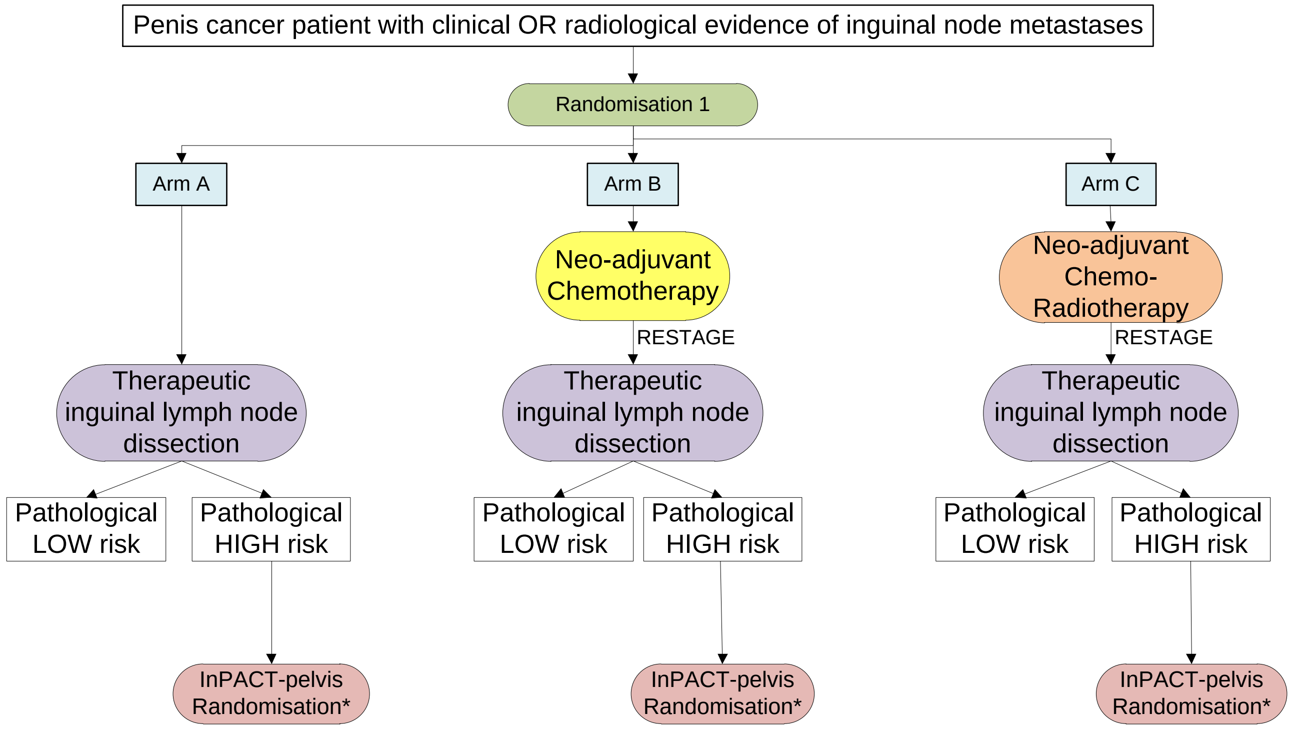

*Consent for InPACT-pelvis randomisation needed

InPACT-pelvis

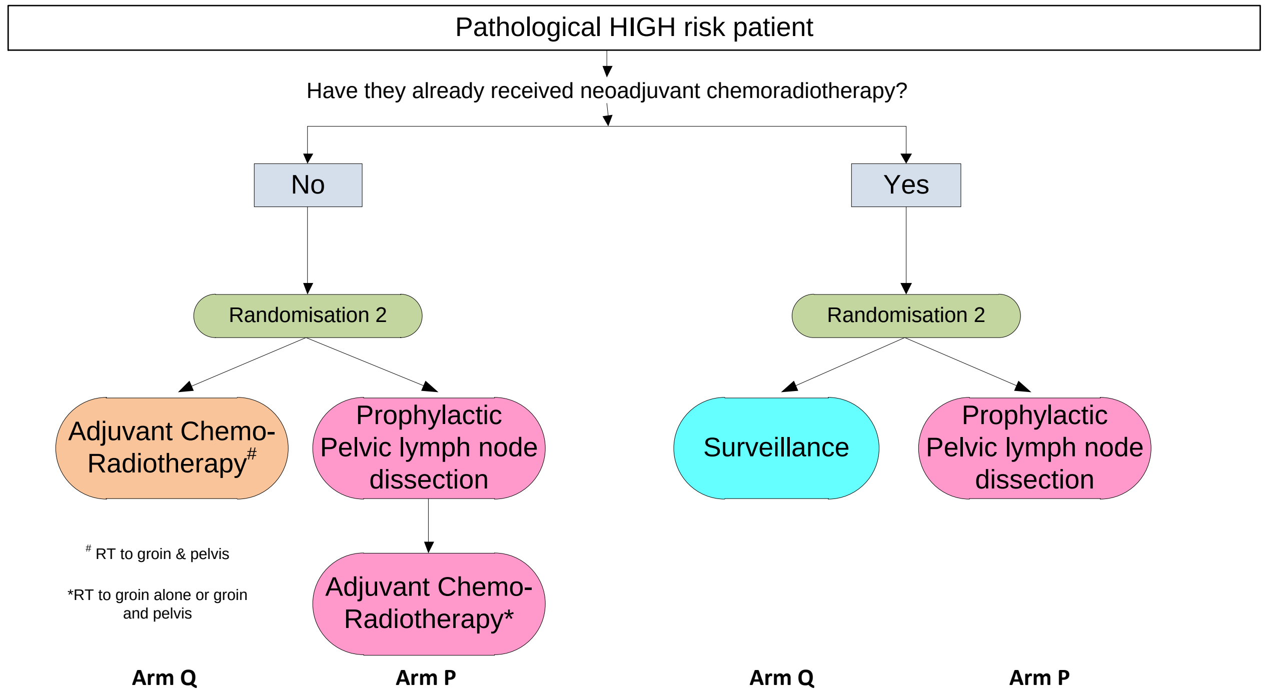

1. INTRODUCTION

1.1. Background

Penis cancer is a rare disease with a limited body of evidence on which to base the majority of management decisions. The UK registered 558 new cases of penis cancer (1.5/100,000 population) in 2011 [1]; 1570 new cases were reported in the US in 2013 [2]. Locoregional lymph node status is the most clinically-significant prognostic factor [3,4]. Risks of recurrence and of mortality are directly related to inguinal disease burden. Five-year survival for patients with a single involved inguinal lymph node is in the region of 80%, whilst for patients with pelvic node involvement (N3) reported 5-year survival figures range from 0 to 12%[3,4].

Conventional management for patients with clinically- or radiologically-overt involvement of groin nodes is therapeutic inguinal lymph node dissection (ILND). Neoadjuvant chemotherapy has been explored in the phase II setting, identifying high response rates [5,6], but the impact of such an approach on survival outcomes has not been assessed. The use of synchronous chemoradiotherapy has traditionally been in two scenarios: as palliative treatment for patients with locally-advanced disease who are not fit for surgery; and as adjuvant therapy following groin dissection where disease has infiltrated through the wall of the lymph node ("extra-capsular extension" or "extra-nodal extension"). Neoadjuvant chemoradiotherapy is of interest because of the high risk of locoregional recurrence and the subsequent increased risk of death after recurrence [4], but this approach has not been formally tested in penis cancer. The InPACT study will assess the value, toxicity and deliverability of both neoadjuvant strategies in patients with inguinal lymph node metastases.

Prophylactic pelvic lymph node dissection (PLND) is often performed in patients whose ILND reveals extra-capsular extension or high nodal disease burden (i.e. conferring a high likelihood of pelvic and distant relapse)[7]. The incidence of post-operative complications is high, including intractable scrotal and lower limb lymphoedema. It is far from clear whether PLND confers any survival benefit over and above adjuvant chemoradiotherapy, which is commonly used in this scenario. InPACT will address the question of whether there is any added benefit for PLND when given in addition to adjuvant chemoradiotherapy (or following neoadjuvant chemoradiotherapy) over chemoradiotherapy alone.

The last five years have seen a number of developments in penis cancer, but a more cohesive approach is required to define more accurately the evidence base for "standard" management. InPACT, an International Rare Cancers Initiative study, aims to determine the relative benefits and optimum sequencing of surgery, chemotherapy and chemoradiotherapy in the routine clinical management of patients with squamous carcinoma of the penis who have inguinal lymph node metastases (i.e. locally-advanced disease). The study may result in a recommended sequence for some or all of these interventions, delivering uniformity of practice and an outcome-related evidence base. This would then be the basis on which to test newer interventions, including the place of targeted therapy in penis cancer management.

1.2. Study rationale

1.2.1. Rationale for investigation of the role of chemoradiotherapy

Penis cancer belongs to a family of squamous carcinomas associated with HPV infection, including anal, cervix and oropharyngeal squamous cell cancers. There is a growing consensus that HPV-related squamous cell tumours are relatively more sensitive to chemotherapy and radiotherapy. Treatment with radiotherapy and chemotherapy is well established in other HPV-related cancers in a range of scenarios: chemoradiotherapy is the standard of care in anal, cervix and oropharyngeal cancer, and adjuvant radiotherapy has been shown to be highly effective in vulval cancer. Parallels in treatment paradigms may be drawn from these other cancers sites. There is some clinical data suggesting that radiotherapy may decrease local recurrence rates in penis cancer [8], but there is no published clinical data on chemoradiotherapy. Generating data in this area is a high priority and could change clinical practice.

1.2.2. Rationale for investigation of the role of neoadjuvant chemo(radio)therapy

Published data on the use of primary or neoadjuvant taxane-platinum chemotherapy in penis cancer shows that some patients are effectively down-staged. The combination of paclitaxel, ifosfamide and cisplatin (TIP) shows high response rates in patients with locally-advanced disease, such that inoperable patients may be rendered operable and even (in 10% of patients) histologically free of cancer[4]. The UK National Cancer Research Institute (NCRI) penile cancer subgroup studied the TPF (docetaxel, cisplatin and 5FU) combination in a more heterogeneous group of patients[9]. That trial (CRUK/09/001) recruited 29 patients in 15 months, demonstrating that it is possible to run studies in this rare tumour within a manageable timeframe. The inferior overall response rate to TPF (38.5%) has led to TIP (response rate 50%) being selected for this current trial, which will study neoadjuvant chemotherapy in a wider group of patients. TIP is internationally recognised as a treatment option in this setting and is considered the reference standard in the US. Neoadjuvant chemotherapy is widely used in head and neck cancer, but randomised trials in cervical cancer and anal cancer have shown no benefit. Quantifying the relative merits of neoadjuvant chemotherapy vs. chemoradiotherapy could, again, change current clinical practice.

1.2.3. Rationale for investigation of the role of prophylactic pelvic lymph node dissection (PLND)

Patients who are found to have extracapsular inguinal lymph node disease at groin dissection (ILND) have a substantial risk of both local recurrence and of the development of pelvic nodal disease. Prophylactic PLND for patients with high-risk groin disease has, therefore, become routine in many major penile cancer centres. There is, however, minimal clinical data on long-term outcomes from this procedure. Adjuvant chemoradiotherapy is regarded as the standard of care for the majority of these patients, and InPACT will address both the additional toxicity and the added value of prophylactic PLND in this patient group.

1.2.4. Role of HPV in penis cancer

Approximately 50% of penile cancers in the UK are associated with human papilloma virus (HPV) infection [10]. The role of HPV in the aetiology of penis cancer [10,11] may be due to its effect on the PTEN/Akt pathway[12]. InPACT will help to identify the prognostic significance of HPV infection in established squamous carcinoma of the penis.

1.3. Risks and benefits of treatment regimens in InPACT

1.3.1. Standard surgery

Inguinal lymph node dissection (ILND) is standard treatment for patients with metastases to the inguinal lymph nodes. The known or intended benefit is eradication of macroscopic disease. Risks include:

- Haematoma.
- Lymphatic leak or seroma.
- Infection.
- Necrosis of wound edges.
- Lymphoedema.

These risks are not expected to be greater in the InPACT trial population than in the non-trial population undergoing therapeutic ILND as part of routine care.

1.3.2. Neoadjuvant chemotherapy

Neoadjuvant chemotherapy has been adopted as standard treatment in some centres for selected patient groups (mainly those patients with a heavy burden of lymph node disease), but it cannot be regarded as the international standard of care. The known, potential or intended benefits are:

- Reduction in the volume of metastatic disease, up to and including pathological complete remission.

- Improvement in locoregional disease control.
- Improvement in overall survival and/or disease-free survival.
- Improvement in quality of life.

Known risks of neoadjuvant chemotherapy using the TIP regimen are:

- Nausea, vomiting, mucositis, constipation and/or diarrhoea.
- Haematological toxicity, including febrile neutropenia, thrombocytopenia and anaemia.
- Biochemical/renal toxicity, including electrolyte disturbances and acute kidney injury.
- Neurological toxicity, including sensory neuropathy and reversible ifosfamide-induced encephalopathy.

Potential or unquantified risks are:

- Detrimental outcome due to the delay in surgery engendered by administering chemotherapy.
- Death or lasting morbidity due to chemotherapy toxicity.

The rates of toxicity for this regimen are described for its use in other tumour types and, to a limited extent, in the neoadjuvant setting in penis cancer. Data from this study will further describe the regimen's safety profile.

1.3.3. Neoadjuvant chemoradiotherapy

Chemoradiotherapy (radiotherapy to the inguinal node basin with concomitant weekly cisplatin) is in routine use in penis cancer as both adjuvant therapy and as definitive therapy where inguinal disease is inoperable, or where the patient is not fit for surgery. There is no primary source of evidence on which to base an assessment of the risks and benefits of neoadjuvant chemoradiotherapy in penis cancer.
The potential or intended benefits are:

- Reduction in the volume of metastatic disease, up to and including pathological complete remission.
- Improvement in locoregional disease control.
- Improvement in overall survival and/or disease-free survival.
- Improvement in quality of life.

Known risks of chemoradiotherapy to involved inguinal node basins are:

- Exposure of / toxicity to skin, bladder, bowels, spine, femoral heads, and testicles/scrotum.
- Nausea, vomiting, constipation and/or diarrhoea.
- Haematological toxicity, typically thrombocytopenia, but including febrile neutropenia and anaemia.
- Biochemical/renal toxicity, including electrolyte disturbances and acute kidney injury.
- Neurological toxicity limited to sensory neuropathy.

These risks are not expected to be greater in the InPACT trial population than in the historical non-trial population.

Potential or unquantified risks are:

- Detrimental outcome due to the delay in surgery engendered by administering chemoradiotherapy.
- Lymphoedema, which may be worse than that seen with either surgery or chemoradiotherapy alone.
- Increased rate or increased severity of surgical morbidity compared to that seen with surgery alone (see above).

1.3.4. Adjuvant chemoradiotherapy following ILND

Post-operative or adjuvant chemoradiotherapy (radiotherapy to the inguinal node basin with concomitant weekly cisplatin) is in routine use in penis cancer. The potential or intended benefits are:

- Improvement in locoregional disease control.
- Improvement in overall survival and/or disease-free survival.
- Improvement in quality of life.

Known risks of post-operative chemoradiotherapy to involved inguinal node basins are:

- Exposure of / toxicity to skin, bladder, bowels, spine, femoral heads, and testicles/scrotum.
- Nausea, vomiting, constipation and/or diarrhoea.
- Haematological toxicity, typically thrombocytopenia, but including febrile neutropenia and anaemia.
- Biochemical/renal toxicity, including electrolyte disturbances and acute kidney injury.
- Neurological toxicity limited to sensory neuropathy.
- Lymphoedema, which may be worse than that seen with surgery alone.

These risks are not expected to be greater in the InPACT trial population than in the non-trial population where adjuvant chemoradiotherapy is given as part of usual care.

Potential or unquantified risks are:

- Increased rate or increased severity of surgical morbidity compared to that seen with surgery alone (see above).

1.3.5. Prophylactic pelvic lymph node dissection (PLND) and chemoradiotherapy following ILND

Post-operative/adjuvant chemoradiotherapy and prophylactic pelvic lymph node dissection are both in routine use for selected patients who have undergone inguinal lymph node dissection. The risks of PLND are similar to those of ILND. The potential benefits of PLND are:

- Improvement in locoregional disease control.
- Improvement in overall survival and/or disease-free survival.

The potential risk of the combination of PLND and post-operative chemoradiotherapy is a worsening in all those risks detailed for both modalities.

The potential benefits are additional improvement in locoregional disease control and/or survival.

2. TRIAL OBJECTIVES

2.1. Primary objective

Two questions regarding the management of patients with locally-advanced squamous carcinoma of the penis are addressed as primary objectives:

1. (a) Is there a role for neoadjuvant therapy and, if so, (b) does chemotherapy or chemoradiotherapy produce superior outcomes (either for survival endpoints or for morbidity/quality of life endpoints)?
2. What is the additional survival benefit of prophylactic pelvic lymph node dissection (PLND) given after neoadjuvant chemoradiotherapy or with adjuvant chemoradiotherapy of the pelvic nodes over and above that of chemoradiotherapy alone in patients at high risk of recurrence following ILND?

These questions are addressed through sequential randomisations (InPACT-neoadjuvant and InPACT-pelvis) at two key decision points in the clinical management pathway.

2.2. Secondary objectives

In InPACT-neoadjuvant:

- Can neoadjuvant therapy prior to surgery (ILND) reduce recurrence rates?
- Which is the more active of neoadjuvant chemotherapy or neoadjuvant chemoradiotherapy?
- What is the operative/post-operative complication rate following neoadjuvant therapy of both types?
- Is neoadjuvant chemoradiotherapy feasible in this setting?

In InPACT-pelvis:

- What is the rate of additional complications for the combination of PLND and chemoradiotherapy?

2.3. Exploratory objectives

Two additional questions will be addressed, in participating patients:

- What is the relationship between human papillomavirus (HPV) status and outcome for all groups studied?
- What is the impact on quality of life of the (sequential) treatments studied?

3. TRIAL DESIGN

This is an international phase III trial, with a Bayesian design, incorporating two sequential randomisations. It efficiently examines a series of questions that routinely arise in the sequencing of treatment. The study design has evolved from lengthy international consultation that has enabled us to build consensus over which questions arise from current knowledge and practice. It will enable potential randomisation for the majority of patients with inguinal lymph node metastases and will provide data to inform future clinical decisions.

InPACT-neoadjuvant patients are prospectively stratified by disease burden as assessed by radiological criteria. Treatment options are then defined according to the disease burden strata. Treatment is allocated by randomisation where more than one treatment is considered suitable (see section 5.5). Patients may be allocated to one of three initial treatments:

A. standard surgery (ILND);
B. neoadjuvant chemotherapy followed by standard surgery (ILND); or
C. neoadjuvant chemoradiotherapy followed by standard surgery (ILND).

After ILND, patients are defined as being at low or high risk of recurrence based on histological interpretation of the ILND specimen. Patients at high risk of relapse are eligible for InPACT-pelvis, where they are randomised to either:

P. prophylactic PLND;
Q. no prophylactic PLND.

All patients in InPACT-pelvis receive chemoradiotherapy at some point in their treatment pathway:

- InPACT-pelvis patients who <u>have not</u> received chemoradiotherapy in the neoadjuvant setting prior to ILND will receive PLND followed by adjuvant chemoradiotherapy (arm P) OR adjuvant chemoradiotherapy alone (arm Q).
- Those patients who <u>have</u> already received chemoradiotherapy in the neoadjuvant setting are randomised to PLND (arm P) or surveillance (arm Q).

This is a complex study, with any patient being potentially offered up to four separate interventions (neoadjuvant chemotherapy/chemoradiotherapy, ILND, PLND, adjuvant chemoradiotherapy). It is entirely possible therefore, that some patients may decline randomisation to certain treatment arms. Randomisation in InPACT-neoadjuvant is encouraged based on disease burden, but the trial design is pragmatic, accepting that some patients may be included (non-randomised) in any of the trial treatment arms. This will reduce the unbiased information available in some arms of the trial if it were to become a common occurrence. Non-randomised patients will not be included in the primary comparative analyses, but their inclusion will provide prospective, supportive data and will allow subsequent entry into InPACT-pelvis if appropriate. The aim is to identify eligible patients prior to ILND i.e. through InPACT-neoadjuvant. Recruitment to InPACT-pelvis will be monitored and direct entry to the trial at this second randomisation point may be considered by amendment if advised by the independent Trial Steering Committee.

4. STUDY OUTCOME MEASURES

4.1. Primary outcome measure

- The primary outcome measure (all patients) is survival time.

4.2. Secondary outcome measures

Secondary outcome measures for the trial as a whole (all patients) are:

- Disease-specific survival time.
- Disease-free survival time with subsidiary outcomes of locoregional recurrence free survival time and distant metastasis free survival time.
- Toxicity and specifically the occurrence of at least one grade 3 or 4 adverse event.
- Occurrence of surgical complication.
- Feasibility of pathological nodal assessment after chemotherapy.
- Quality of life (in participating patients).

Secondary outcome measures for InPACT-neoadjuvant are:

- Occurrence of pathological complete remission.
- Operability.
- Feasibility of on-schedule delivery of neoadjuvant therapy.

Secondary outcome measures for InPACT-pelvis are:

- Occurrence of lower limb/scrotal oedema.

5. PATIENT SELECTION AND ELIGIBILITY

5.1. Number of participants

The aim is to recruit 200 participants internationally.

5.2. Source of participants

Cancer of the penis is a rare disease and there is a requirement that all patients are managed under the auspices of a supranetwork multidisciplinary team. Discussion of all eligible patients by the relevant supranetwork MDT is mandatory.
Non-UK patients will be recruited in accordance with the relevant group-specific appendix.

5.3. Inclusion criteria

All inclusion criteria must be met for both InPACT-neoadjuvant and subsequently InPACT-pelvis, unless otherwise specified. Timelines for assessments to confirm eligibility are detailed in section 8.1 of the protocol.

Inclusion criteria for InPACT-neoadjuvant

1. Male, aged 18 years or older.
2. Histologically-proven squamous cell carcinoma of the penis.
3. Stage:
   - any T, N1 (i.e. a palpable mobile unilateral inguinal lymph node OR a single radiologically-abnormal inguinal lymph node with no evidence of extra-nodal extension), M0 or;
   - any T, N2 (i.e. palpable mobile multiple or bilateral inguinal lymph nodes OR radiologically evident multiple or bilateral inguinal nodes with no evidence of extra-nodal extension), M0 or;
   - any T, N3 (i.e. fixed inguinal nodal mass or any pelvic lymphadenopathy), M0
4. Patients being considered for InPACT-neoadjuvant must have either:
   - measurable disease as determined by RECIST (version 1.1) criteria or;
   - a single, unilateral lymph node that does not meet RECIST criteria for measurable disease, but that is either palpable or radiologically abnormal, and with histological/cytological evidence of metastatic involvement. <u>This applies only to low disease burden patients who would then be eligible for direct entry into arm A.</u>

   **Patients with radiological evidence of macroscopic pelvic node involvement are eligible for randomisation in InPACT-neoadjuvant but are not be eligible for entry into InPACT-pelvis.*
5. Performance Status ECOG 0, 1 or 2.
6. Patient is fit to receive the randomisation options for which he is being considered.
7. Haematology/biochemistry (as dictated by local hospital practice) should indicate fitness for randomisation options and parameters should be in line with considerations specified in the summary of product characteristics. Haematological parameters should not be supported by transfusion to enable entry into the trial. Liver function and renal function tests must form part of the pre-treatment assessment for patients who may be randomised to receive TIP chemotherapy e.g. patients with impaired renal function may not be considered for arms B and C of InPACT-neoadjuvant (see below) but may be considered for arm A.
8. Nodal disease burden must be assessable, with all patients stratified into one of three categories (low / intermediate / high disease burden) in accordance with physical examination and Graafland radiological criteria (see Section 5.5.1).
9. Glomerular filtration rate (GFR) must be assessed for all patients (see Section 5.5.2). Requirements for GFR vary with treatment eligibility:-
   - GFR ≥50 mL/min: eligible for all treatment options
   - GFR 45–49 mL/min: eligible for surgery alone (arm A) and for synchronous chemoradiotherapy (arm C); not eligible for neoadjuvant TIP chemotherapy
   - GFR < 45 mL/min eligible for surgery alone (arm A) or radiotherapy (arm C with cisplatin omitted)
10. Willing and able to comply with follow-up schedule.
11. Written informed consent.

Inclusion criteria for InPACT-pelvis

1. Patient has met eligibility criteria for InPACT-neoadjuvant.
2. Patient has completed ILND within arms A, B or C of InPACT-neoadjuvant.
3. There must be no radiological evidence of residual inguinal disease on cross-sectional imaging performed after therapeutic inguinal lymph node dissection.
4. There must be no radiological evidence of pelvic lymphadenopathy on cross-sectional imaging performed after therapeutic inguinal lymph node dissection.
   **Any patient who underwent synchronous ispilateral pelvic lymph node dissection at the time of inguinal lymph node dissection is automatically ineligible for InPACT Pelvis.*
5. Patient must be at high risk of relapse following ILND, risk of relapse being assigned on the basis of histological assessment of the ILND specimen. High-risk disease is defined as any patient where ILND reveals either: extranodal extension, bilateral nodal involvement, or 3 or more involved nodes. These patients should be considered at high risk of harbouring occult micrometastatic disease in the ipsilateral pelvic nodes.
6. Performance Status ECOG 0, 1 or 2.
7. Patient is fit to receive the randomisation options for which he is being considered.
8. Haematology/biochemistry (as dictated by local hospital practice) should indicate fitness for randomisation options and parameters should be in line with considerations specified in the summary of product characteristics. Haematological parameters should not be supported by transfusion to enable entry into the trial.
9. Willing and able to comply with follow-up schedule.
10. Written informed consent.

5.4. Exclusion criteria

Patients who have any of the following are not eligible:

1. Pure verrucous carcinoma of the penis.
2. Non-squamous malignancy of the penis.
3. Squamous carcinoma of the urethra.
4. Stage M1.
5. Previous systemic chemotherapy or chemoradiotherapy outside of the InPACT trial.
6. Any absolute contraindication to chemotherapy if eligible for a chemotherapy/chemoradiotherapy randomisation.
7. Concurrent malignancy (other than SCC or Basal Cell Carcinoma of non-penile skin) that has required surgical or non-surgical treatment in the last 3 years.
8. Patients who are sexually active and unwilling to use effective contraception (if they are not already surgically sterile).
9. Radiological evidence of macroscopic pelvic lymph node disease on post-ILND cross-sectional imaging (InPACT-pelvis only).
10. Patients with regionally advanced (N1-3, M0) penile cancer with disease burden that is considered unresectable by the accredited InPACT surgeon* utilising standard inguinal, ilioinguinal lymphadenectomy resection and reconstructive techniques. For example, where procedures would require circumferential resection of the femoral or iliac vessels, or the requirement for hemipelvectomy.
    ** InPACT surgeon should consider reviewing the case with their National InPACT surgical lead where resectablity is unclear.*

Randomisation between various combinations of treatment arms is permitted subject to the following additional criteria:

5.5. InPACT-neoadjuvant additional eligibility criteria

5.5.1. Nodal disease burden

Low disease burden

Patients with a single unilateral inguinal lymph node are classified as having a low disease burden (see Table 3) and are not eligible for the randomised component of InPACT-neoadjuvant, i.e. are not eligible to receive neoadjuvant therapy within the trial. Such patients should be entered directly into InPACT-neoadjuvant Arm A without randomisation and they will be treated with ILND in accordance with guidance for Arm A. Data will be collected to facilitate secondary analyses, including subsequent randomisation in InPACT-pelvis (if eligible), assessment of quality of life (optional) and HPV status.

Intermediate or high disease burden

Patients with intermediate or high disease burden, (high-risk disease on the radiological criteria of Graafland - see table 1, below) are considered suitable to receive neoadjuvant therapy and, in the absence of any absolute contraindication to chemotherapy, are eligible for the randomised component of InPACT-neoadjuvant. (Patients with absolute contraindication to chemotherapy should be entered directly into InPACT-neoadjuvant Arm A without randomisation (see Table 3) and they will be treated with ILND in accordance with guidance for Arm A).

- Patients with radiological evidence of the unilateral involvement of two nodes (see Table 2, below) will be randomised between surgery (Arm A), neoadjuvant chemotherapy (Arm B) or neoadjuvant chemoradiotherapy (Arm C) in a 2:1:1 ratio.

- For patients with bilateral disease or with high disease burden the preference is randomisation between neoadjuvant chemotherapy (Arm B) and neoadjuvant chemoradiotherapy (Arm C) (1:1). "High-risk disease" is defined by Graafland et al [13] as 3 or more involved nodes in an inguinal basin, or extra-nodal extension, or pelvic node involvement, and is regarded as synonymous with "high disease burden" for the purposes of this trial. The presence of central nodal necrosis or irregular nodal border *per se* is correlated with pathologically high-risk disease being found at surgery, therefore patients with either central nodal necrosis or irregular nodal border should be considered to have radiological evidence of high disease burden / high-risk disease, and be randomised between arms B and C.

Table 1 - Graafland radiological criteria

	Predictive of involvement of an individual node	Predictive of high risk disease
Short axis diameter >15mm	Yes	No
Central nodal necrosis	Yes	Yes
Irregular nodal border	Yes	Yes
Infiltration of adjacent tissue	Yes	Yes

I. High-risk disease is defined as 3 or more involved nodes in an inguinal basin, or extra-nodal extension, or pelvic node involvement.

II. Ipsilateral pelvic nodal disease is highly unlikely (negative predictive value 100%) if both central nodal necrosis and irregular nodal border are absent from the inguinal nodes.

5.5.2. Glomerular filtration rate (GFR)

General principles

Various methods are used to calculate GFR, and all have their inaccuracies. Measured GFR (by isotopic methods) is more accurate, but is not routinely repeated prior to each cycle of chemotherapy and therefore GFR should be assessed according to both the MDRD formula **and** the Cockcroft & Gault formula (see Appendix 3). Measured GFR is encouraged either where this is local standard of care prior to

commencing treatment, or when a calculated GFR is <5ml/min below eligibility criteria using either the MDRD formula or the Cockcroft & Gault formula.

Secondary research questions relating to GFR
Core data will be collected on each patient at trial entry enabling a direct comparison of GFR calculations according to the MDRD formula and the Cockcroft & Gault equation. These will be compared, where available, with GFR that has been measured (by isotopic methods). Not only will this provide clinical data that can be related to patients' trial entry, it can also be reviewed in relation to subsequent renal toxicity.

Eligibility for InPACT-neoadjuvant treatment arms according to renal function (Table 2)
Patients who are eligible to receive either/all treatment option(s) should be randomised according to nodal burden. GFR is not a key consideration for patients entering directly into arm A (ILND).

Good GFR – 50ml/min or greater:
Neoadjuvant chemotherapy (arm B): A GFR of 50ml/min or greater is acceptable for neoadjuvant chemotherapy (arm B) and patients should be assessed with regard to their fitness for the TIP regimen.
Neoadjuvant chemoradiotherapy (arm C): A GFR of 50ml/min or greater is also acceptable for neoadjuvant chemoradiotherapy (arm C), and patients should similarly be assessed with regard to their fitness to receive concurrent cisplatin.

Poor GFR – less than 50ml/min
Any patient whose GFR is below 50ml/min is not considered suitable for neoadjuvant chemotherapy (arm B), but may be suitable for neoadjuvant chemoradiotherapy (arm C) if GFR≥45mls/min (see section 9.4 of the protocol); they should be assessed for their fitness to receive the latter and they should be randomised 1:1 to surgery (arm A) or neoadjuvant chemoradiotherapy (arm C) where appropriate (any patient whose GFR is below 45mls/min, may still be considered for randomisation between arm A and arm C, with the omission of concurrent cisplatin if allocated to arm C (see Table 2 below)).

Table 2 - Eligibility for InPACT-neoadjuvant treatment arms according to renal function

GFR ml/min	Possible treatment arm*
≥50	A, B or C
≥45-<50	A, C
<45	A, C (omit concurrent cisplatin)<br>or adjuvant chemoradiotherapy (omit concurrent cisplatin)

*A – ILND; B- neoadjuvant chemotherapy followed by ILND; C – neoadjuvant chemoradiotherapy followed by ILND.

5.5.3. Radiological evidence of macroscopic pelvic node involvement

Patients with radiological evidence of macroscopic pelvic lymph node involvement are eligible for randomisation in InPACT-neoadjuvant. The preferred option for these patients is randomisation between neoadjuvant chemotherapy (arm B) and neoadjuvant chemoradiotherapy (arm C) (as for other patients with high burden of disease). Randomisation between surgery (arm A) and neoadjuvant therapy (arms B and C; or arm C if GFR<50ml/min) will be permitted, but such patients cannot be assigned to surgery alone (arm A) without randomisation if they are to remain in the trial.

Any patients with radiological evidence of macroscopic pelvic lymph node involvement will not be eligible for Randomisation 2, InPACT-pelvis.

All InPACT patients with macroscopic pelvic lymph node disease shall be offered chemoradiotherapy; this will either be given as part of their neoadjuvant randomisation, or it will be given post-operatively as adjuvant therapy.

5.5.4. Role of PET-CT scanning in determining eligibility for InPACT-neoadjuvant

Concerns about the sensitivity of PET-CT in identifying nodal metastases in cancer of the penis [14, 15] means that conventional staging with CT or CT plus MRI remains the preferred approach in this study.

There is some evidence, however, that PET scans may identify nodal disease not seen on conventional CT or MRI scanning [16] There is also interest in the use of PET scanning to identify patients most likely to benefit from neoadjuvant chemotherapy [17]. Many centres now use PET scans as part of their pre-operative assessment for patients with loco-regional lymph node involvement, although PET scans are not universally recognised as the standard of care.

- PET scanning is not mandated as part of InPACT.
- Disease identified on PET scans that was not evident on CT or MRI may lead to a change in a patient's stage. The highest radiological stage (incorporating findings from any PET scans) should be used when assigning a patient to a disease burden category. Relevant examples related to InPACT:
    - FDG-avid inguinal nodes not meeting RECIST criteria (via CT or MRI) would need histologic confirmation prior to trial entry into InPACT
    - FDG-avid pelvic node(s) not meeting CT or MRI RECIST criteria should have histologic confirmation prior to randomisation in order to assign TNM stage. If histologically confirmed, patient would be eligible for InPACT-neoadjuvant but not InPACT-pelvis.
    - FDG-avidity at other sites (extra-pelvic lymph nodes, other viscera) must be explained by known co-morbidity or must have metastasis excluded on biopsy for the patient to be eligible for InPACT.

ASSESSMENT OF GRAAFLAND CRITERIA

The co-localisation CT scan used in PET-CT scanning is not of sufficient resolution to enable assessment of inguinal nodes by Graafland criteria. Conventional CT assessment of a single node may demonstrate central necrosis or an irregular border or extranodal extension – all features that predict for occult pelvic node involvement. Such factors would affect InPACT disease burden status and randomisation options. All patients who are entering InPACT-Neoadjuvant & who do not have pelvic node involvement on PET scan must, therefore, have had CT or MRI imaging of the pelvis in accordance with trial entry criteria.

5.5.5. Summary of InPACT-neoadjuvant randomisation/treatment options

All patients must meet the eligibility criteria described in 5.3 and 5.4. Patients are eligible for various randomisation options or for trial entry directly to a treatment arm without randomisation in InPACT-neoadjuvant (as described above) and summarised in Table 3.

Participating centres are encouraged to engage with the InPACT trial office prior to entering a patient onto the trial, to discuss trial entry pathways and ensure all understanding of the proposed trial entry/randomisation options.

Any questions relating to randomisation/treatment options should be directed to:
In the UK / mainland Europe: the InPACT Trial Manager at ICR-CTSU:
Email: InPACT-icrctsu@icr.ac.uk General enquiries: +44 (0)208 722 4238.

In the US and South America: the ECOG-ACRIN Operations Office in Boston. Please call during normal business hours for protocol related questions and ask for the DA for EA8134:
Email: ea8134team@jimmy.harvard.edu General enquiries: (857)504-2900.

Table 3 Burden of disease risk stratification for treatment allocation in InPACT-neoadjuvant

			**Direct entry**	**Randomisation**			**Direct entry**	**Direct entry**
Disease burden	Inclusions	GFR ml/min	**ARM A Surgery**	**ARMs A B C Surgery vs neoCT vs neochemoRT**	**ARMs A C Surgery vs neochemoRT**	**ARMs B C neoCT vs neochemoRT**	**ARM B neoCT**	**ARM C neochemoRT**
LOW	One mobile node with no high risk features on CT	Any	TRIAL ENTRY					
INTER-MEDIATE	Two ipsilateral mobile nodes with no high risk features on CT	≥50	TRIAL ENTRY	**RANDOMISE**				
		<50	TRIAL ENTRY		**RANDOMISE**			
HIGH	Bilateral nodes, pelvic nodes, or fixed nodes, or radiological evidence of 3 or more involved nodes OR; the presence of high risk features on CT	≥50	TRIAL ENTRY ***n.b. not if pelvic lymph nodes involved (see 5.5.3)***	**RANDOMISE**		**RANDOMISE**	TRIAL ENTRY	
		<50	TRIAL ENTRY ***n.b. not if pelvic lymph nodes involved (see 5.5.3)***		**RANDOMISE**			TRIAL ENTRY

(white)	Recommended option for trial inclusion
(grey)	Permitted at investigator's discretion
(black)	Not permitted in trial

5.6. InPACT-pelvis (randomisation 2) additional eligibility criteria

All patients must meet the eligibility criteria described in 5.3 and 5.4. All patients must be at high risk of relapse following ILND, risk of relapse being assigned on the basis of histological assessment of the ILND specimen.

There must be no radiological evidence of residual inguinal disease or pelvic lymphadenopathy on cross-sectional imaging performed after therapeutic inguinal lymph node dissection.

High-risk disease is defined as any patient whose ILND reveals either extranodal extension, bilateral nodal involvement, or 3 or more involved nodes. These patients should be considered at high risk of harbouring occult micrometastatic disease in the ipsilateral pelvic nodes. They are eligible for randomisation 2 between prophylactic pelvic lymph node dissection (PLND) and surveillance.

- Eligible patients who have not received neoadjuvant chemoradiotherapy will be randomised to prophylactic PLND followed by adjuvant chemoradiotherapy (arm P) or immediate adjuvant chemoradiotherapy (arm Q).

- If chemoradiotherapy has been delivered in the neoadjuvant setting, additional chemoradiotherapy will not be given since the pelvic nodes will have been included in the treatment field; such patients are therefore randomised to prophylactic PLND (arm P) or surveillance (arm Q).

5.7. Life style guidelines

Participants who are sexually active must be surgically sterile or must agree to use effective contraception during the period of therapy and for 6 months after the last dose of study treatment. Effective

contraception is defined as double barrier contraception (e.g. condom plus spermicide in combination with a diaphragm, cervical cap or intrauterine device).

6. SCREENING

6.1. Screening log

Participating sites will be required to keep a log of all participants with squamous carcinoma of the penis that have inguinal lymph node metastases (i.e. locally-advanced disease) that are potentially eligible for this study. This log will capture the following information:

- Date patient identified
- Screening outcome (patient approached/accepted participation/declined participation)
- Reasons for not approaching / declining participation (if available)
- Trial ID (if applicable)

This information will be used to monitor recruitment activity. No patient identifiable data will be sent to the data centre at this stage.

Screening information will be collected from non-UK sites in accordance with the relevant group specific appendix.

6.2. Procedure for obtaining informed consent

The Principal Investigator (or designated individual) must ensure that each trial patient is fully informed about the nature and objectives of the trial and possible risks associated with participation. Participants should be given the current REC/Institutional Review Board approved InPACT patient information sheet for their consideration. Patients should only be asked to consent to the study after they have had sufficient time to consider the trial, and the opportunity to ask any further questions.

No protocol-specific assessments should be conducted until the InPACT consent form has been signed and dated by both the patient and the Investigator, unless they are performed routinely as part of standard patient care.

Patients will be asked to consent at two different time points. Patients will be provided with information for both InPACT-neoadjuvant and InPACT-pelvis before the first randomisation, but will initially only be asked to consent to InPACT-neoadjuvant. Following histological assessment after ILND, those patients who are eligible will be offered entry into InPACT-pelvis, with appropriate discussion and informed consent being taken.

UK patients who consent to InPACT will be asked to consent to donate archival tissue for translational studies, detailed in section 23.1. Patients should be made aware that participation in the InPACT translational sub-study is entirely voluntary. Refusal to participate in the InPACT translational sub- study will not result in ineligibility to participate in the main clinical trial and will not impact the medical care received.

Patients should be offered participation in the InPACT Quality of Life Questionnaire (QL) Sub-Study, described in section 23.2 and Appendix 4. Patients should be made aware that participation in the QL Sub-Study is entirely voluntary. Refusal to participate in the QL Sub-Study will not result in ineligibility to participate in the main clinical trial and will not impact the medical care received.

Confirmation of the patient’s consent and the informed consent process must be documented in the patient’s medical notes. A copy of the signed consent form should be provided to the patient and the

original retained in the investigator site file, which must be available for verification by the Data Centre [or their delegates] study staff or for regulatory inspection at any time.

6.3. Participation in other clinical trials

Patients who fulfil the eligibility criteria will be given the opportunity to participate in InPACT even if they have participated in other clinical trials prior to recruitment.

Patients should not participate in any other trials of investigational medicinal products whilst they are receiving active treatment in InPACT.

7. TRIAL ENTRY/RANDOMISATION

ICR-CTSU encourages PIs to consider equality, diversity and inclusion when recruiting patients into its trials. UK patients must be entered into the trial centrally by the UK data centre (ICR-CTSU) before trial treatment can commence. Details for trial entry for non-UK centres are given in the relevant group specific appendix.

UK patients should be entered into the trial by emailing ICR-CTSU to request a call back via:

randomisation-icrctsu@icr.ac.uk

09.00-17.00 (UK time) Monday to Friday

7.1. InPACT-neoadjuvant – trial entry or randomisation

Individual patients may not be eligible for randomisation into all arms of the trial. Patients with low burden disease who are not suitable for neoadjuvant treatment must be registered prospectively to be considered part of InPACT-neoadjuvant (receiving arm A) and to be potentially eligible for InPACT-pelvis.

Trial entry into InPACT-neoadjuvant should take place within 4 weeks of planned start of treatment.

An InPACT-neoadjuvant eligibility and trial entry checklist must be completed prior to trial entry.

The following information will be required at trial entry for all patients:

- Name of hospital, consultant and person randomising patient;
- Confirmation that patient is eligible by completion of the eligibility checklist;
- Confirmation that patient has given written informed consent for the study and whether or not they have consented to participate in any of the optional sub-studies;
- Patient's full name, hospital number, date of birth, postcode/zip code and NHS/CHI number;
- Disease burden risk group, GFR (if intermediate or high disease burden), confirmation of unilateral or bilateral disease.

Eligible patients will be entered into the trial in accordance with the burden of disease risk stratification (see section 5.5.5).

Low risk patients:
The caller will be given the patient's unique trial identification number. The UK data centre will confirm patient entry into the study in writing to the data management contact at the recruiting site.

Intermediate/high risk patients:

The caller will be given the patient's unique trial identification number and treatment allocation. The UK data centre will confirm patient entry into the trial and their treatment allocation in writing to the data management contact and pharmacist (if applicable) at the recruiting site.

7.2. InPACT-pelvis – randomisation

Assigned treatment in InPACT-pelvis should commence no earlier than 6 weeks after ILND, with PLND or adjuvant chemoradiotherapy commencing no more than 12 weeks after ILND. Randomisation into InPACT-pelvis can take place at any time after confirmation of high-risk disease following post-operative risk classification and prior to the planned InPACT-pelvis treatment start date.

Low-risk patients entered into arm A of InPACT-neoadjuvant may subsequently relapse. Those with surgically-managed inguinal recurrence may then become eligible for randomisation into InPACT-pelvis.

The InPACT-pelvis eligibility and randomisation checklist must be completed prior to randomisation.

For patients previously entered into InPACT-neoadjuvant, the following information will be required at randomisation into InPACT-pelvis:

- Patient Trial ID previously allocated to the patient in InPACT-neoadjuvant;
- Confirmation that patient is eligible for this randomisation by completion of the InPACT-pelvis eligibility checklist;
- Confirmation that patient has given written informed consent for this randomisation (randomisation 2) and whether or not they have consented to participate in any of the optional sub-studies;
- Post-operative risk classification;
- Confirmation of whether there is extracapsular involvement (yes/no);
- Confirmation of previous neoadjuvant treatment received (none, chemotherapy, chemoradiotherapy);
- GFR.

The caller will be given the treatment allocation.

The UK data centre will confirm patient entry into the trial in writing to the data management contact at the recruiting site.

8. TRIAL ASSESSMENTS

8.1. Baseline assessments

The following assessments should be conducted within 14 days prior to trial entry unless otherwise stated below (into InPACT-neoadjuvant or InPACT-pelvis);

- Physical examination (including height, weight and body surface area) to assess fitness and ECOG performance status.
- Cross-sectional imaging (preferably by CT scan of chest, abdomen and pelvis, but alternatively by MRI of pelvis and abdomen with CT of chest, where appropriate) to be performed within 4 weeks of trial entry and no more than 6 weeks prior to scheduled start date of trial treatment (RECIST v1.1 for InPACT-neoadjuvant only).
- ECG.
- Haematology and biochemistry (as dictated by local hospital practice) to assess fitness for randomisation options in line with the summary of product characteristics (SmPC).

Haematological parameters should not be supported (by transfusion or growth factors) to enable entry into the trial.

- GFR to be assessed according to the MDRD formula **and** the Cockcroft & Gault formula (see Appendix 3).
- Quality of life assessment (EORTC QLQ-C30, adapted GCLQ) (in participating patients).
- Baseline assessment of symptoms using CTC scoring (CTCAE v4).
- InPACT-neoadjuvant only: Histological confirmation (by fine needle aspiration) of nodal involvement is routine for most patients in most units, but although this is preferred it is not mandatory where lymph node disease is not in doubt clinically.
- InPACT-neoadjuvant only: HPV status for those centres where this is collected as part of their standard practice (does not have to be within 14 days prior to trial entry).
- Details of the post-operative risk classification are required for patients who are being entered into InPACT-pelvis (randomisation 2).

8.2. On-treatment assessments – InPACT-neoadjuvant

8.2.1. Arm A - standard surgery (ILND)

The total treatment duration is estimated to be 1 day for those patients allocated to arm A - standard surgery (ILND); inpatient stay of up to 14 days may then be required. Patients will be seen, treated and assessed according to the surgical guidance notes. Investigators should check that they are referencing the latest version of the surgical guidance notes (available from ICR-CTSU and on the trial web page). The following assessments should be conducted for the purposes of InPACT within 30 days (± 7 days) post-surgery unless otherwise specified:

- Toxicity assessment (Modified Clavien-Dindo classification (See Appendix 6) at 30 days (± 15 days) post-surgery.
- Cross-sectional imaging if the clinician suspects that the patient's disease has progressed.

8.2.2. Arm B – neoadjuvant chemotherapy + ILND

The total treatment time for neoadjuvant chemotherapy (Arm B) is estimated to be 12 weeks (4 cycles of chemotherapy). Patients will be seen and assessed prior to each cycle of treatment to assess fitness for chemotherapy, and at other time-points according to local practice.

Neoadjuvant chemotherapy: the following assessments should be conducted prior to each cycle of chemotherapy for the purposes of InPACT:

- Toxicity assessment CTC v4 (in order to assess toxicity from the previous cycle).
- GFR
- Cross-sectional imaging if the clinician suspects that the patient's disease has progressed (RECIST v1.1).
- Haematology and biochemistry
- GFR (using MDRD formula **AND** Cockcroft & Gault (see Appendix 3).

Restaging visit: the following assessments should be conducted at the restaging visit, a maximum of 4 weeks from day 1 of the final cycle of chemotherapy and prior to ILND:

- Cross-sectional imaging (RECIST v1.1)
- Haematology and biochemistry
- Toxicity assessment CTC v4

ILND /post-surgery: Patients will proceed to therapeutic inguinal lymph node dissection upon completion of chemotherapy. The date of ILND is to be no more than 12 weeks after day 1 of the last cycle of chemotherapy. The following assessments should be conducted within 30 days (± 7 days) post-surgery unless otherwise specified.

- Haematology and biochemistry
- GFR
- Toxicity assessment CTC v4
- Toxicity assessment (Modified Clavien-Dindo classification (See Appendix 6) at 30 days (± 15 days) post-surgery.

8.2.3. Arm C – neoadjuvant chemoradiotherapy + ILND

The total treatment duration of neoadjuvant chemoradiotherapy (arm C) is estimated to be 5 weeks. Patients will be seen and assessed at fixed time-points, according to local practice.

Neoadjuvant chemoradiotherapy: the following trial assessments must be conducted at 3 weeks timed from day 1 of trial treatment:

- Toxicity assessment CTC v4.
- Cross-sectional imaging is required only if the clinician suspects disease progression (this should be reported to RECIST v1.1 if found).
- Haematology and biochemistry
- GFR (using MDRD formula **AND** Cockcroft & Gault (see Appendix 3).

Restaging visit: the following assessments should be conducted at the restaging visit, a maximum of 4 weeks following the final fraction of radiotherapy) and prior to ILND:

- Cross-sectional imaging (RECIST v1.1)
- Haematology and biochemistry

ILND/post-surgery: Patients will proceed to therapeutic inguinal lymph node dissection upon completion of chemoradiotherapy. The date of ILND is to be no more than 12 weeks after the final fraction of radiotherapy. The following assessments should be conducted within 30 days (± 7 days) post-surgery unless otherwise specified.

- Haematology and biochemistry
- GFR
- Toxicity assessment CTC v4
- Toxicity assessment (Modified Clavien-Dindo classification (See Appendix 6) at 30 days (± 15 days) post-surgery.
- RTOG toxicity assessment (toxicity scoring by Radiation Therapy Oncology Group (RTOG) criteria).

8.3. On treatment assessments – InPACT-pelvis

Any patient whose ILND reveals either extra-nodal extension, bilateral nodal involvement, or 3 or more involved nodes in one groin should be considered at high risk of harbouring occult micrometastatic disease in the ipsilateral pelvic nodes. These patients are eligible for randomisation into InPACT-pelvis.

If a patient is considered at high risk of harbouring occult micrometastatic disease in the ipsilateral pelvic nodes, but does not proceed to randomisation into InPACT-pelvis, that patient should be considered for adjuvant chemoradiotherapy (please see section 8.4).

Patients entering InPACT-pelvis who have not received chemoradiotherapy in the neoadjuvant setting (i.e. prior to ILND) will be randomised to receive PLND followed by adjuvant chemoradiotherapy (arm P) OR to receive adjuvant chemoradiotherapy alone (arm Q). Those patients who have already undergone chemoradiotherapy in the neoadjuvant setting are randomised to PLND (arm P) or to surveillance (arm Q).

8.3.1. Arm P – prophylactic PLND

The total treatment duration is estimated to be 1 day for those patients allocated to arm P – prophylactic PLND; an inpatient stay of up to 14 days may then be required. Treatment duration will be an additional 5 weeks for those patients who are to receive adjuvant chemoradiotherapy. Patients will be seen and assessed prior to treatment and at other time-points according to local practice.

Following surgery:

- Toxicity assessment using Modified Clavien-Dindo classification (See Appendix 6) at 30 days (± 15 days) post-surgery.

The following assessments should be conducted at 6 weeks from day 1 of PLND for patients who do not receive adjuvant chemoradiotherapy and at week 3 of treatment for patients who do receive adjuvant chemoradiotherapy:

- Toxicity assessment CTC v4.
- Cross-sectional imaging if the clinician suspects that the patient's disease has recurred.
- Haematology and biochemistry
- GFR (using MDRD formula **AND** Cockcroft & Gault (see Appendix 3)).

8.3.2. Arm Q – no prophylactic PLND

The total treatment duration for those patients allocated to arm Q (no prophylactic PLND) who are to receive adjuvant chemoradiotherapy (i.e. those patients who did not receive neoadjuvant chemoradiotherapy) is 5 weeks. Patients will be seen and assessed at fixed time-points according to local practice.

The following assessments should be conducted at 6 weeks from the decision to start surveillance for those who do not require adjuvant chemoradiotherapy or 3 weeks from day 1 of start of chemoradiotherapy for patients who do receive adjuvant chemoradiotherapy:

- Cross-sectional imaging if the clinician suspects that the patient's disease has recurred.
- Toxicity assessment CTC v4.
- Haematology and biochemistry
- GFR (using MDRD formula **AND** Cockcroft & Gault (see Appendix 3).

8.4. Adjuvant chemoradiotherapy for patients not proceeding to InPACT-pelvis

Any patient whose ILND reveals either extra-nodal extension, bilateral involved nodes or 3 or more involved nodes in one groin should be considered at high risk of harbouring occult micrometastatic disease in the ipsilateral pelvic nodes. If for any reason, the patient does not proceed to randomisation into InPACT-pelvis, and has not undergone neoadjuvant chemoradiotherapy, they should be considered for adjuvant chemoradiotherapy.

The following assessments should be conducted at week 3 of chemoradiotherapy:

- Cross-sectional imaging if the clinician suspects that the patient's disease has recurred.

- Toxicity assessment CTC v4.
- Haematology and biochemistry
- GFR (using MDRD formula **AND** Cockcroft & Gault (see Appendix 3)).

8.5. Follow-up

Follow-up for the purposes of InPACT encompasses all on-treatment and post-treatment visits across both InPACT-neoadjuvant and InPACT-pelvis. A total of 5 years of data will be captured for each patient.

All patients will undergo clinical review in accordance with the guidelines of the European Association of Urology (EAU) [18]: every 3 months for years 1 and 2, then every 6 months for years 3, 4 and 5, timed from day 1 of study treatment.

Due to the different sequences of treatment that patients may receive in the trial, some patients will have a longer "on treatment" period and a shorter off-treatment "follow-up" period than others. For example in year 1 the different sequences of treatments mean that a patient seen at approximately 3 months could be attending for treatment, for restaging, or for a post-treatment follow-up visit depending on which arm of the trial they are in.

Once a patient has completed all study treatment, they will be deemed "off treatment" and post-treatment follow-up visits will commence. The maximum number of post-treatment follow-up visits is 14.

A patient participating only in InPACT-neoadjuvant shall be deemed to have completed all study treatment on the day that ILND was conducted. The only exceptions to this will be patients in arm A (surgery alone) or arm B (neoadjuvant chemotherapy followed by surgery) who are found to have high-risk disease in their dissection specimen, and who are therefore mandated to receive adjuvant chemoradiotherapy. These patients' study treatment is deemed completed on the final day of adjuvant chemoradiotherapy.

Any patient entered into InPACT–neoadjuvant and who proceeds to randomisation into InPACT-pelvis will be deemed to have completed study treatment either:-

- on the day of prophylactic pelvic lymph node dissection (arm P) for patients who received neoadjuvant chemoradiotherapy in InPACT-neoadjuvant or
- at the end of adjuvant chemoradiotherapy (arms P and Q), or
- on the day of ILND for patients who received neoadjuvant chemoradiotherapy in InPACT-neoadjuvant and who are allocated to surveillance (no PLND - arm Q).

The following assessments should be conducted, timed from the start of study treatment:

- Cross-sectional imaging (either CT scan of chest, abdomen and pelvis (preferred method) or MRI of pelvis and abdomen with CT of chest) – at months 6, 12, 18 & 24.
- Toxicity assessment CTC v4 – at months 3, 9, 15, 21, 30, 36, 42, 48, 54 & 60.
- RTOG to monitor radiotherapy late toxicity for those patients who have received chemoradiotherapy – at months 3, 9, 15, 21, 30, 36, 42, 48, 54 & 60.
- QL assessments at baseline (prior to randomisation), post-neoadjuvant treatment (arms B and C only), post-ILND, 12 months from the start of treatment, and then at 18, 24 and 36 months.

These assessments are the required minimum for trial purposes, but they do not exclude the use of other modalities (e.g. U/S of inguinal basins, or the use of PET-CT scans) that may be used as local standard of

care. In order not to introduce reporting bias, any additional imaging used as part of local standard care should be employed for all trial patients irrespective of their InPACT treatment allocation.

8.6. Procedure at disease progression/recurrence

Patients who progress during neoadjuvant treatment but prior to ILND, or who have disease recurrence after ILND, should be managed in accordance with local hospital policy. Patients should continue to be followed up for trial endpoints including overall survival.

8.7. Discontinuation from treatment or follow-up

Participants may discontinue trial treatment at any time at their own request, or they may be discontinued at the discretion of the Principal Investigator. Reasons for discontinuation may include:

- Disease progression/recurrence
- Unacceptable toxicity

Participants who discontinue certain treatments, may proceed to the next treatment in the sequence should they still be deemed fit to do so by the treating clinician. If a patient discontinues all trial treatments in the sequence, they should continue with trial-specific follow-up.

If a patient withdraws consent for further follow-up, it should be clarified whether they no longer wish to attend trial-specific follow-up visits, or whether they wish to stop contributing further data to the study. A trial deviation form should be completed for any patient who withdraws consent for information to be sent to the Data Centre or who declines to attend trial follow-up visits. If a patient requests that their data is removed from the study entirely, the implications of this should be discussed with the patient first to ensure that this is their intent and, if confirmed, the Data Centre should be notified in writing. If this request is received after results have been published the course of action will be agreed between the Sponsor and independent Trial Steering Committee.

Patients who receive any off-protocol chemotherapy before inguinal lymph node dissection will be followed-up according to the protocol schedule. Further treatment will be at the discretion of the clinician.

8.8. Pathology review

8.8.1. Primary tumour and lymph node review (prior to randomisation into InPACT) and response review

Procedures for primary tumour review and response review (ILND specimens) will be the same and review will occur at the same time. A random sample of non-responders will also be selected for central review.

All patients must have their primary tumour pathology and lymph node biopsy reviewed by at least one of the specialist InPACT review pathologists (see section 8.8.3). Tumours should be reported, staged and graded according to the 2017 Penile and Distal Urethral tumour RCPath Tumour reporting Guidelines which uses TNM8. All UK primary tumour pathology reports should be sent to the named UK pathologist, who will determine how many representative slides will need to be sent for review. The full text of the pathology report must be available to the InPACT review pathologist, including the macroscopic and microscopic description and detailed block key, together with any specimen photographs and diagrams. Country-specific procedures are further discussed in the group specific appendix.

Pathology review will follow the guidelines detailed in The Royal College of Pathologists Standards and datasets for reporting cancers: Dataset for penile and distal urethral cancer histopathology reports. This can be found at https://www.rcpath.org/profession/guidelines/cancer-datasets-and-tissue-pathways.html

Where the initial pathology has not been reported by one of the InPACT review pathologists, the following should be undertaken:

Following review of the original report(s) submitted by the site to the InPACT lead pathologist, all or a selection of slides (as decided by the InPACT lead pathologist) from both primary penile tumours and lymph node biopsies will be scanned at sufficient resolution to allow full histopathology review.

The review should take place on the primary tumour following definitive treatment (resection). If, however, there has been biopsy but no resection then the review will take place on the biopsy.

Details of where and to whom the slides and/or blocks (as dictated by the InPACT lead pathologist) from the primary tumours and lymph node biopsies should be sent are given in the pathology section of the Trial Guidance notes.

If there is a discrepancy between the initial local review and the central review then the case will be further reviewed by another central review pathologist to try and reach consensus. The second central review pathologist will be asked to review the case within a 7-day period to allow a maximum reporting turnaround of 28 days.

8.8.2. Post-operative risk classification

Post-operative (ILND) risk classification will be centrally reviewed by the InPACT lead pathologist to include review of the pathology report. These reviews will need to take place in real time as the post-operative risk classification is required for eligibility for randomisation into InPACT-pelvis. Where the post-operative risk classification has not been undertaken by one of the InPACT review pathologists, the process summarised in 8.8.1 should be followed.

8.8.3. Pathology quality assurance (QA)

The InPACT lead pathologist will approve individuals as InPACT review pathologists. All UK pathologists designated as InPACT review pathologists will be part of the UK National Penile Pathology subgroup (“The Hobnobs”) and each will need to be responsible for reporting and reviewing at least 25 new cases of penis cancer per year. They will also be required to participate in the UK Uropathology EQA scheme, which includes penile pathology cases.

8.9. Radiology review of staging scans

Each site will have to identify a radiologist who will be accredited to review InPACT staging scans. The accreditation process is more fully described within the Trial Guidance Notes. All patients will have initial staging scans reviewed by the participating centre’s accredited radiologist. Specific procedures for this review are discussed within the Trial Guidance notes.

All patients undergoing neoadjuvant therapy (of either modality) must have their scans reported to RECIST v1.1 (see Appendix 7) and details of response or progression recorded on the electronic case report forms:

Disease progression is defined as a significant increase in the size of pre-existing disease according to RECIST criteria (an increase of at least 20% in the longest diameter, see Appendix 7) or the development of new metastases.

Recurrence is defined as the development of new metastases post-ILND in a patient who was previously in complete clinical remission. Recurrence may be:

Local: at the primary site
Regional: from the primary site to the local lymph node basin
Distant: beyond the locoregional lymph nodes

Details of all recurrences must be included on the case report forms along with their subsequent clinical response.

8.10. Schedules of assessments

The schedules of assessments depict the schedule of patient visits throughout the study, based on initial arm allocation in InPACT-neoadjuvant and, where applicable, the subsequent arm allocation in InPACT-pelvis.

In InPACT-pelvis, patients may commence treatment at the earliest time-point of 6 weeks after ILND and no later than 12 weeks after ILND. Randomisation into InPACT-pelvis can take place at any time after confirmation of high-risk disease following post-operative risk classification and prior to the planned InPACT-pelvis treatment start date. The earliest time point for starting InPACT-pelvis treatment (6 weeks after ILND) is used to inform the schedules of assessment provided here.

Following entry into InPACT-neoadjuvant, patients should be seen and followed up according to the relevant schedule of assessment for InPACT-neoadjuvant arms A (table 4), B (table 7) and C (table 10).

Should patients subsequently go on to participate in InPACT-pelvis, patients should be seen and followed-up according to the relevant schedule of assessment for InPACT-pelvis arm P (table 5, 8 or 11) or Q (table 6, 9 or 12).

As detailed in section 8.5, depending on time on treatment, the schedule for follow-up might differ between patients based on treatment windows specified throughout the protocol, which may result in the patients first follow-up time point differing from that specified in the schedules below; therefore a patient specific schedule can be obtained from the UK data centre if needed.

In InPACT-pelvis, patients who received neo-adjuvant chemoradiotherapy will **not** go on to receive adjuvant chemoradiotherapy however these patients should undergo the same assessments as those receiving adjuvant chemoradiotherapy.

NOTE: The assessments detailed in the schedules are the minimum required as part of InPACT. Further assessments are dictated by local hospital policy.

The following tables are to be used in conjunction with the applicable areas of the protocol to establish all required interventions and timelines and should not be referred to in isolation.

Table 4 InPACT-neoadjuvant – Arm A (standard surgery (ILND)) only; low risk cohort and patients allocated Arm A in randomisation 1

		Trial Entry/ Randomisation InPACT-neoadjuvant	Baseline	ILND SURGERY	Follow-up 1	Follow-up 2	Follow-up 3	Follow-up 4	Follow-up 5	Follow-up 6	Follow-up 7	Follow-up 8	Follow-up 9	Follow-up 10	Follow-up 11	Follow-up 12	Follow-up 13	Follow-up 14
	**APPROXIMATE TIME ON TRIAL (from start of treatment[1])**			Start of treatment (4 weeks from trial entry)	(3 months)	(6 months)	(9 months)	(12 months)	(15 months)	(18 months)	(21 months)	(24 months)	(30 months)	(36 months)	(42 months)	(48 months)	(54 months)	(60 months)
Assessments	InPACT-neoadjuvant Trial Entry/Randomisation	X																
	Physical Examination		X															
	Haematology & Biochemistry		X															
	ECG		X															
	GFR[2]		X															
	Cross-sectional imaging[5]		X		X	X		X		X		X						
	Tumour Assessment RECISTv1.1		X															
	CTCAEv4.0 toxicity criteria[6]		X		X		X		X		X		X	X	X	X	X	X
	Quality of life questionnaire[7]		X	X				X		X		X		X				
	Inguinal Lymph Node Dissection (ILND)			X														
	ILND Histology review[4]			X														
	Modified Clavien-Dindo (Surgical Complications)[3]			X														
	Follow-up				X	X	X	X	X	X	X	X	X	X	X	X	X	X

[1]Patients will be seen approximately every 3 months for years 1 and 2 and then every 6 months for years 3-5 timed from the start of their treatment (in accordance with the guidelines of the European Association of Urology (EAU)). [2]GFR using MDRD formula and Cockcroft & Gault (see Appendix 3). [3]Modified Clavien-Dindo to be assessed 30(±15) days post-surgery. [4]ILND Histology review 6 weeks after ILND.

Patients will have the following assessments carried out:

- [5]Cross-sectional imaging (preferred method is CT scan of chest, abdomen & pelvis. Alternatively, MRI of pelvis and abdomen with CT of chest) at baseline, months 3, 6, 12, 18 & 24.
- [6]Toxicity assessment CTC v4 at baseline, months 3, 9, 15, 21, 30, 36, 42, 48, 54 & 60.
- [7]QL questionnaire: at baseline, post ILND treatment, 12, 18, 24 and 36 months.

Table 5 InPACT-neoadjuvant – Arm A (ILND) followed by InPACT-pelvis Arm P (Prophylactic PLND + Adjuvant Chemoradiotherapy)

		Trial Entry InPACT-neoadjuvant	Baseline	ILND SURGERY	Randomisation InPACT-pelvis	Baseline	Prophylactic PLND SURGERY	Adjuvant Chemoradiotherapy (treatment duration: 5 weeks)	Follow-up 1	Follow-up 2	Follow-up 3	Follow-up 4	Follow-up 5	Follow-up 6	Follow-up 7	Follow-up 8	Follow-up 9	Follow-up 10	Follow-up 11	Follow-up 12	Follow-up 13
	**APPROXIMATE TIME ON TRIAL (from start of treatment[1])**			Start of treatment (4 weeks from trial entry)			3 months	~4.5 months	(6 months)	(9 months)	(12 months)	(15 months)	(18 months)	(21 months)	(24 months)	(30 months)	(36 months)	(42 months)	(48 months)	(54 months)	(60 months)
Assessments	InPACT-neoadjuvant Trial Entry/Randomisation	X																			
	Physical Examination		X			X															
	Haematology & Biochemistry		X			X		X[5]	X												
	ECG		X																		
	GFR[2]		X			X		X[5]	X												
	Cross-sectional imaging[7]		X			X		X[5,6]	X		X		X		X						
	Tumour Assessment RECIST v1.1		X																		
	CTCAEv4.0 toxicity criteria[8]		X			X		X[5]		X		X		X		X	X	X	X	X	X
	Quality of life booklet[9]		X	X							X		X		X		X				
	Inguinal Lymph Node Dissection (ILND)			X																	
	ILND Histology review[4]			X																	
	Modified Clavien-Dindo (Surgical Complications)[3]			X			X														
	InPACT-pelvis Randomisation				X																
	Prophylactic Pelvic Lymph Node Dissection (PPLND)						X														
	Adjuvant Chemoradiotherapy							X													
	RTOG[8]									X		X		X		X	X	X	X	X	X
	Follow-up								X	X	X	X	X	X	X	X	X	X	X	X	X

[1]Patients will be seen approximately every 3 months for years 1 and 2 and then every 6 months for years 3-5 timed from the start of their treatment (in accordance with the guidelines of the European Association of Urology (EAU)). [2]GFR using MDRD formula and Cockcroft & Gault (see Appendix 3). [3]Modified Clavien-Dindo to be assessed 30(±15) days post-surgery. [4]ILND Histology review 6 weeks after ILND and prior to randomisation into InPACT-pelvis. [5] Assessments only at week 3 of chemoradiotherapy. [6]Repeated cross-sectional imaging if the clinician suspects that the patient's disease has progressed.

Patients will have the following assessments carried out:

- [7]Cross-sectional imaging (preferred method is CT scan of chest, abdomen & pelvis. Alternatively MRI of pelvis and abdomen with CT of chest) – at baseline, months 3, 6, 12, 18 & 24.
- [8]Toxicity assessment CTC v4 and RTOG at baseline (except RTOG), months 3, 9, 15, 21, 30, 36, 42, 48, 54 & 60
- [9]QL questionnaire: at baseline, post ILND treatment, 12, 18, 24 and 36 months.

Table 6 InPACT-neoadjuvant – Arm A (ILND) followed by InPACT-pelvis Arm Q (Surveillance + Adjuvant Chemoradiotherapy)

		Trial Entry InPACT-neoadjuvant	Baseline	ILND SURGERY	Randomisation InPACT-pelvis	Baseline	Adjuvant Chemoradiotherapy (treatment duration: 5 weeks)	Follow-up 1	Follow-up 2	Follow-up 3	Follow-up 4	Follow-up 5	Follow-up 6	Follow-up 7	Follow-up 8	Follow-up 9	Follow-up 10	Follow-up 11	Follow-up 12	Follow-up 13
	**APPROXIMATE TIME ON TRIAL (from start of treatment[1])**			Start of treatment (4 weeks from trial entry)			(3 months)	(6 months)	(9 months)	(12 months)	(15 months)	(18 months)	(21 months)	(24 months)	(30 months)	(36 months)	(42 months)	(48 months)	(54 months)	(60 months)
Assessments	InPACT-neoadjuvant Trial Entry/Randomisation	X																		
	Physical Examination		X			X														
	Haematology & Biochemistry		X			X	X[5]	X												
	ECG		X																	
	GFR[2]		X			X	X[5]	X												
	Cross-sectional imaging[7]		X			X	X[5,6]	X		X		X		X						
	Tumour Assessment RECIST v1.1		X																	
	CTCAEv4.0 toxicity criteria[8]		X			X	X[5]		X		X		X		X	X	X	X	X	X
	Quality of life booklet[9]		X	X						X		X		X		X				
	Inguinal Lymph Node Dissection (ILND)			X																
	ILND Histology review[4]			X																
	Modified Clavien-Dindo (Surgical Complications)[3]			X																
	InPACT-pelvis Randomisation				X															
	Adjuvant Chemoradiotherapy						X													
	RTOG[8]								X		X		X		X	X	X	X	X	X
	Follow-up							X	X	X	X	X	X	X	X	X	X	X	X	X

[1]Patients will be seen approximately every 3 months for years 1 and 2 and then every 6 months for years 3-5 timed from the start of their treatment (in accordance with the guidelines of the European Association of Urology (EAU)). [2]GFR using MDRD formula and Cockcroft & Gault (see Appendix 3). [3]Modified Clavien-Dindo to be assessed 30(±15) days post-surgery. [4]ILND Histology review 6 weeks after ILND and prior to randomisation into InPACT-pelvis. [5] Assessments only at week 3 of chemoradiotherapy. [6]Repeated cross-sectional imaging if the clinician suspects that the patient's disease has progressed.

Patients will have the following assessments carried out:

- [7]Cross-sectional imaging (preferred method is CT scan of chest, abdomen & pelvis. Alternatively MRI of pelvis and abdomen with CT of chest) at baseline, months 3, 6, 12, 18 & 24.
- [8]Toxicity assessment CTC v4 and RTOG at baseline (except RTOG), months 3, 9, 15, 21, 30, 36, 42, 48, 54 & 60
- [9]QL questionnaire: at baseline, post ILND treatment, 12, 18, 24 and 36 months.

Table 7 InPACT-neoadjuvant – Arm B (neoadjuvant chemotherapy + ILND) only

		Trial Entry InPACT-neoadjuvant	Baseline / Screening	Neoadjuvant Chemotherapy (Treatment duration: 4 weeks)	Restage	ILND SURGERY	Follow-up 1	Follow-up 2	Follow-up 3	Follow-up 4	Follow-up 5	Follow-up 6	Follow-up 7	Follow-up 8	Follow-up 9	Follow-up 10	Follow-up 11	Follow-up 12	Follow-up 13
	**APPROXIMATE TIME ON TRIAL (from start of treatment[1])**			Start of treatment (4 weeks from trial entry)	(3 months)	(18 weeks)	(6 months)	(9 months)	(12 months)	(15 months)	(18 months)	(21 months)	(24 months)	(30 months)	(36 months)	(42 months)	(48 months)	(54 months)	(60 months)
Assessments	InPACT-neoadjuvant Randomisation	X																	
	Physical Examination		X																
	Haematology & Biochemistry		X	X	X	X													
	ECG		X																
	GFR[2]		X	X		X													
	Cross-sectional imaging[8]		X	X[6]	X		X		X		X		X						
	Tumour Assessment RECISTv1.1		X		X														
	CTCAEv4.0 toxicity criteria[9]		X	X[5]	X	X[7]		X		X		X		X	X	X	X	X	X
	Quality of life booklet[10]		X		X	X			X		X		X		X				
	Neoadjuvant Chemotherapy			X															
	Inguinal Lymph Node Dissection (ILND)					X													
	ILND Histology review[4]					X													
	Modified Clavien-Dindo (Surgical Complications)[3]					X													
	Follow-up						X	X	X	X	X	X	X	X	X	X	X	X	X

[1]Patients will be seen approximately every 3 months for years 1 and 2 and then every 6 months for years 3-5 timed from the start of their treatment (in accordance with the guidelines of the European Association of Urology (EAU)). [2]GFR using MDRD formula and Cockcroft & Gault (see Appendix 3). [3]Modified Clavien-Dindo to be assessed 30(±15) days post-surgery. [4] ILND Histology review 6 weeks after ILND. [5] Toxicity assessment CTC v4 (in order to assess toxicity from the previous cycle) to be assessed prior to each cycle. [6] Repeated cross-sectional imaging if the clinician suspects that the patient's disease has progressed. [7] Toxicity assessment CTC v4 to be assessed prior to surgery.

Patients will have the following assessments carried out:

- [8]Cross-sectional imaging (preferred method is CT scan of chest, abdomen & pelvis. Alternatively MRI of pelvis and abdomen with CT of chest) at baseline, restage (3 months), months 6, 12, 18 & 24.
- [9]Toxicity assessment CTC v4 and RTOG at baseline (except RTOG), months 3, 9, 15, 21, 30, 36, 42, 48, 54 & 60
- [10]QL questionnaire: at baseline, post-neoadjuvant treatment, post ILND treatment, 12, 18, 24 and 36 months.

Table 8 InPACT-neoadjuvant – Arm B (neoadjuvant chemotherapy + ILND) followed by InPACT-pelvis Arm P (Prophylactic PLND + Adjuvant chemoradiotherapy)

		Trial Entry InPACT-neoadjuvant	Baseline / Screening	Neoadjuvant Chemotherapy (Treatment duration: 4 weeks)	Restage	ILND SURGERY	Randomisation InPACT-pelvis	Baseline	Prophylactic PLND SURGERY	Adjuvant Chemoradiotherapy (treatment duration: 5 weeks)	Follow-up 1	Follow-up 2	Follow-up 3	Follow-up 4	Follow-up 5	Follow-up 6	Follow-up 7	Follow-up 8	Follow-up 9	Follow-up 10	Follow-up 11	Follow-up 12
	**APPROXIMATE TIME ON TRIAL (from start of treatment[1])**			Start of treatment (4 weeks from trial entry)	(3 months)	(18 weeks)		(6 months)		(~34 weeks)	(9 months)	(12 months)	(15 months)	(18 months)	(21 months)	(24 months)	(30 months)	(36 months)	(42 months)	(48 months)	(54 months)	(60 months)
Assessments	InPACT-neoadjuvant Randomisation	X																				
	Physical Examination		X					X														
	Haematology & Biochemistry		X	X	X	X		X		X[8]	X											
	ECG		X																			
	GFR[2]		X	X		X		X		X[8]	X											
	Cross-sectional imaging[9]		x	X[6]	X			X		X[6,8]		X		X		X						
	Tumour Assessment RECISTv1.1		X		X																	
	CTCAEv4.0 toxicity criteria[10]		X	X[5]	X	X[7]		X		X[8]	X		X		X		X		X	X	X	X
	Quality of life booklet[11]		X		X	X						X		X		X		X				
	Neoadjuvant Chemotherapy			X																		
	Inguinal Lymph Node Dissection (ILND)					X																
	ILND Histology review[4]					X																
	Modified Clavien-Dindo (Surgical Complications)[3]					X			X													
	InPACT-pelvis Randomisation						X															
	Prophylactic Pelvic Lymph Node Dissection								X													
	Adjuvant Chemoradiotherapy									X												
	RTOG[10]										X		X		X		X		X	X	X	X
	Follow-up										X	X	X	X	X	X	X	X	X	X	X	X

[1]Patients will be seen approximately every 3 months for years 1 and 2 and then every 6 months for years 3-5 timed from the start of their treatment (in accordance with the guidelines of the European Association of Urology (EAU)). [2]GFR using MDRD formula and Cockcroft & Gault (see Appendix 3). [3]Modified Clavien-Dindo to be assessed 30(±15) days post-surgery. [4]ILND Histology review 6 weeks after ILND and prior to randomisation into InPACT-pelvis. [5] Toxicity assessment CTC v4 (in order to assess toxicity from the previous cycle) to be assessed prior to each cycle. [6] Repeated cross-sectional imaging if the clinician suspects that the patient's disease has progressed. [7]Toxicity assessment CTC v4 to be assessed prior to surgery. [8]Assessments only at week 3 of chemoradiotherapy.

Patients will have the following assessments carried out:

- [9]Cross-sectional imaging (preferred method is CT scan of chest, abdomen & pelvis. Alternatively MRI of pelvis and abdomen with CT of chest) at baseline, restage (3 months), months 6, 12, 18 & 24.
- [10]Toxicity assessment CTC v4 and RTOG at baseline (except RTOG), months 3 (except RTOG), 9, 15, 21, 30, 36, 42, 48, 54 & 60
- [11]QL questionnaire: at baseline, post-neoadjuvant treatment, post ILND treatment, 12, 18, 24 and 36 months.

Table 9 InPACT-neoadjuvant – Arm B (neoadjuvant chemotherapy + ILND) followed by InPACT-pelvis Arm Q (Surveillance + Adjuvant chemoradiotherapy)

		Trial Entry InPACT-neoadjuvant	Baseline / Screening	Neoadjuvant Chemotherapy (Treatment duration: 4 weeks)	Restage	ILND SURGERY	Randomisation InPACT-pelvis	Baseline	Adjuvant Chemoradiotherapy (treatment duration: 5 weeks)	Follow-up 1	Follow-up 2	Follow-up 3	Follow-up 4	Follow-up 5	Follow-up 6	Follow-up 7	Follow-up 8	Follow-up 9	Follow-up 10	Follow-up 11	Follow-up 12
	**APPROXIMATE TIME ON TRIAL (from start of treatment[1])**			Start of treatment (4 weeks from trial entry)	(3 months)	(18 weeks)			(6 months)	(9 months)	(12 months)	(15 months)	(18 months)	(21 months)	(24 months)	(30 months)	(36 months)	(42 months)	(48 months)	(54 months)	(60 months)
Assessments	InPACT-neoadjuvant Randomisation	X																			
	Physical Examination		X					X													
	Haematology & Biochemistry		X	X	X	X		X	X[8]	X											
	ECG		X																		
	GFR[2]		X	X		X		X	X[8]	X											
	Cross-sectional imaging[9]		X	X[6]	X			X	X[6,8]		X		X		X						
	Tumour Assessment RECISTv1.1		X		X																
	CTCAEv4.0 toxicity criteria[10]		X	X[5]	X	X[7]		X	X[8]	X		X		X		X		X	X	X	X
	Quality of life booklet[11]		X		X	X					X		X		X		X				
	Neoadjuvant Chemotherapy			X																	
	Inguinal Lymph Node Dissection (ILND)					X															
	ILND Histology review[4]					X															
	Modified Clavien-Dindo (Surgical Complications)[3]					X															
	InPACT-pelvis Randomisation						X														
	Adjuvant Chemoradiotherapy								X												
	RTOG[10]									X		X		X		X		X	X	X	X
	Follow-up									X	X	X	X	X	X	X	X	X	X	X	X

[1]Patients will be seen approximately every 3 months for years 1 and 2 and then every 6 months for years 3-5 timed from the start of their treatment (in accordance with the guidelines of the European Association of Urology (EAU)). [2]GFR using MDRD formula and Cockcroft & Gault (see Appendix 3). [3]Modified Clavien-Dindo to be assessed 30(±15) days post-surgery. [4]ILND Histology review 6 weeks after ILND and prior to randomisation into InPACT-pelvis. [5]Toxicity assessment CTC v4 (in order to assess toxicity from the previous cycle) to be assessed prior to each cycle. [6]Repeated cross-sectional imaging if the clinician suspects that the patient's disease has progressed. [7]Toxicity assessment CTC v4 to be assessed prior to surgery. [8]Assessments only at week 3 of chemoradiotherapy.

Patients will have the following assessments carried out:

- [9]Cross-sectional imaging (preferred method is CT scan of chest, abdomen & pelvis. Alternatively MRI of pelvis and abdomen with CT of chest) at baseline, restage (3 months), months 6, 12, 18 & 24.
- [10]Toxicity assessment CTC v4 and RTOG at baseline (except RTOG), months 3 (except RTOG), 9, 15, 21, 30, 36, 42, 48, 54 & 60
- [11]QL questionnaire: at baseline, post-neoadjuvant treatment, post ILND treatment, 12, 18, 24 and 36 months.

Table 10 InPACT-neoadjuvant – Arm C (neoadjuvant chemoradiotherapy + ILND) only

		Trial Entry InPACT-neoadjuvant	Baseline / Screening	Neoadjuvant Chemoradiotherapy (Treatment duration: 5 weeks)	Restage	ILND SURGERY	Follow-up 1	Follow-up 2	Follow-up 3	Follow-up 4	Follow-up 5	Follow-up 6	Follow-up 7	Follow-up 8	Follow-up 9	Follow-up 10	Follow-up 11	Follow-up 12	Follow-up 13
	**APPROXIMATE TIME ON TRIAL (from start of treatment[1])**			Start of treatment (4 weeks from trial entry)	(6 weeks)	(3 months)	(6 months)	(9 months)	(12 months)	(15 months)	(18 months)	(21 months)	(24 months)	(30 months)	(36 months)	(42 months)	(48 months)	(54 months)	(60 months)
Assessments	InPACT-neoadjuvant Randomisation	X																	
	Physical Examination		X																
	Haematology & Biochemistry		X	X[8]	X	X													
	ECG		X																
	GFR[2]		X	X[8]		X													
	Cross-sectional imaging[8]		X	X[6,8]	X		X		X		X		X						
	Tumour Assessment RECISTv1.1		X		X														
	CTCAEv4.0 toxicity criteria[9]		X	X[5,8]		X[7]		X		X		X		X	X	X	X	X	X
	Quality of life booklet[10]		X		X	X			X		X		X		X				
	Neoadjuvant Chemoradiotherapy			X															
	Inguinal Lymph Node Dissection (ILND)					X													
	ILND Histology review[4]					X													
	Modified Clavien-Dindo (Surgical Complications)[3]					X													
	RTOG[10]					X		X		X		X		X	X	X	X	X	X
	Follow-up						X	X	X	X	X	X	X	X	X	X	X	X	X

[1]Patients will be seen approximately every 3 months for years 1 and 2 and then every 6 months for years 3-5 timed from the start of their treatment (in accordance with the guidelines of the European Association of Urology (EAU)). [2]GFR using MDRD formula and Cockcroft & Gault (see Appendix 3). [3]Modified Clavien-Dindo to be assessed 30(±15) days post-surgery. [4]ILND Histology review 6 weeks after ILND. [5]Toxicity assessment CTC v4 (in order to assess toxicity from the previous weeks). [6]Repeated cross-sectional imaging if the clinician suspects that the patient's disease has progressed. [7]Toxicity assessment CTC v4 to be assessed prior to surgery. [8]Assessments only at week 3 of chemoradiotherapy.
Patients will have the following assessments carried out:

- [9]Cross-sectional imaging (preferred method is CT scan of chest, abdomen & pelvis. Alternatively MRI of pelvis and abdomen with CT of chest) at baseline, restage (3 months), months 6, 12, 18 & 24.
- [10]Toxicity assessment CTC v4 and RTOG at baseline (except RTOG), months 3 (except RTOG), 9, 15, 21, 30, 36, 42, 48, 54 & 60
- [11]QL questionnaire: at baseline, post-neoadjuvant treatment, post ILND treatment, 12, 18, 24 and 36 months.

Table 11 InPACT neoadjuvant – Arm C (neoadjuvant chemoradiotherapy + ILND) followed by InPACT-pelvis Arm P (Prophylactic PLND)

		Trial Entry InPACT-neoadjuvant	Baseline / Screening	Neoadjuvant Chemoradiotherapy (Treatment duration: 5 weeks)	Restage	ILND SURGERY	Randomisation InPACT-pelvis	Baseline	Prophylactic PLND SURGERY	InPACT-pelvis Week 6 assessments	Follow-up 1	Follow-up 2	Follow-up 3	Follow-up 4	Follow-up 5	Follow-up 6	Follow-up 7	Follow-up 8	Follow-up 9	Follow-up 10	Follow-up 11	Follow-up 12
	**APPROXIMATE TIME ON TRIAL (from start of treatment[1])**			Start of treatment (4 weeks from trial entry)	(6 weeks)	(3 months)				(6 months)	(9 months)	(12 months)	(15 months)	(18 months)	(21 months)	(24 months)	(30 months)	(36 months)	(42 months)	(48 months)	(54 months)	(60 months)
Assessments	InPACT-neoadjuvant Randomisation	X																				
	Physical Examination		X																			
	Haematology & Biochemistry		X	X[8]	X	X		X		X												
	ECG		X																			
	GFR[2]		X	X[8]		X		X		X												
	Cross-sectional imaging[9]		X	X[6,8]	X			X		X[6]		X		X		X						
	Tumour Assessment RECISTv1.1		X		X																	
	CTCAEv4.0 toxicity criteria[10]		X	X[5,8]		X[7]		X			X		X		X		X	X	X	X	X	X
	Quality of life booklet[11]		X		X	X						X		X		X		X				
	Neoadjuvant Chemoradiotherapy			X																		
	Inguinal Lymph Node Dissection (ILND)					X																
	ILND Histology review[4]					X																
	Modified Clavien-Dindo (Surgical Complications)[3]					X			X													
	RTOG[10]					X					X		X		X		X	X	X	X	X	X
	InPACT-pelvis Randomisation						X															
	Prophylactic Pelvic Lymph Node Dissection (PPLND)								X													
	Follow-up										X	X	X	X	X	X	X	X	X	X	X	X

[1]Patients will be seen approximately every 3 months for years 1 and 2 and then every 6 months for years 3-5 timed from the start of their treatment (in accordance with the guidelines of the European Association of Urology (EAU)). [2]GFR using MDRD formula and Cockcroft & Gault (see Appendix 3). [3]Modified Clavien-Dindo to be assessed 30(±15) days post-surgery. [4]ILND Histology review 6 weeks after ILND and prior to randomisation into InPACT-pelvis. [5]Toxicity assessment CTC v4 (in order to assess toxicity from the previous weeks). [6]Repeated cross-sectional imaging if the clinician suspects that the patient's disease has progressed. [7]Toxicity assessment CTC v4 to be assessed prior to surgery. [8]Assessments only at week 3 of chemoradiotherapy.

Patients will have the following assessments carried out:

- [9]Cross-sectional imaging (preferred method is CT scan of chest, abdomen & pelvis. Alternatively MRI of pelvis and abdomen with CT of chest) at baseline, restage (3 months), months 6, 12, 18 & 24.
- [10]Toxicity assessment CTC v4 and RTOG at baseline (except RTOG), months 3 (except RTOG), 9, 15, 21, 30, 36, 42, 48, 54 & 60
- [11]QL questionnaire: at baseline, post-neoadjuvant treatment, post ILND treatment, 12, 18, 24 and 36 months.

Table 12 InPACT-neoadjuvant – Arm C (neoadjuvant chemoradiotherapy + ILND) followed by InPACT-pelvis Arm Q (Surveillance)

		Trial Entry InPACT-neoadjuvant	Baseline / Screening	Neoadjuvant Chemoradiotherapy (Treatment duration: 5 weeks)	Restage	ILND SURGERY	Randomisation InPACT-pelvis	Baseline	InPACT-pelvis Week 6 assessments	Follow-up 1	Follow-up 2	Follow-up 3	Follow-up 4	Follow-up 5	Follow-up 6	Follow-up 7	Follow-up 8	Follow-up 9	Follow-up 10	Follow-up 11	Follow-up 12
	**APPROXIMATE TIME ON TRIAL (from start of treatment[1])**			**Start of treatment (4 weeks from trial entry)**	**(6 weeks)**	**(3 months)**			**(6 months)**	**(9 months)**	**(12 months)**	**(15 months)**	**(18 months)**	**(21 months)**	**(24 months)**	**(30 months)**	**(36 months)**	**(42 months)**	**(48 months)**	**(54 months)**	**(60 months)**
Assessments	InPACT-neoadjuvant Randomisation	X																			
	Physical Examination		X																		
	Haematology & Biochemistry		X	X[8]	X	X		X	X												
	ECG		X																		
	GFR[2]		X	X[8]		X		X	X												
	Cross-sectional imaging[9]		X	X[6,8]	X			X	X		X		X		X						
	Tumour Assessment RECISTv1.1		X		X																
	CTCAEv4.0 toxicity criteria[10]		X	X[5,8]		X[7]		X		X		X		X		X	X	X	X	X	X
	Quality of life booklet[11]		X		X	X					X		X		X		X				
	Neoadjuvant Chemoradiotherapy			X																	
	Inguinal Lymph Node Dissection (ILND)					X															
	ILND Histology review[4]					X															
	Modified Clavien-Dindo (Surgical Complications)[3]					X															
	RTOG[10]					X				X		X		X		X	X	X	X	X	X
	InPACT-pelvis Randomisation						X														
	Follow-up									X	X	X	X	X	X	X	X	X	X	X	X

[1]Patients will be seen approximately every 3 months for years 1 and 2 and then every 6 months for years 3-5 timed from the start of their treatment (in accordance with the guidelines of the European Association of Urology (EAU)). [2]GFR using MDRD formula and Cockcroft & Gault (see Appendix 3). [3]Modified Clavien-Dindo to be assessed 30(±15) days post-surgery. [4]ILND Histology review 6 weeks after ILND and prior to randomisation into InPACT-pelvis. [5] Toxicity assessment CTC v4 (in order to assess toxicity from the previous weeks). [6] Repeated cross-sectional imaging if the clinician suspects that the patient's disease has progressed. [7]Toxicity assessment CTC v4 to be assessed prior to surgery. [8]Assessments only at week 3 of chemoradiotherapy.

Patients will have the following assessments carried out:

- [9]Cross-sectional imaging (preferred method is CT scan of chest, abdomen & pelvis. Alternatively MRI of pelvis and abdomen with CT of chest) at baseline, restage (3 months), months 6, 12, 18 & 24.
- [10]Toxicity assessment CTC v4 and RTOG at baseline (except RTOG), months 3 (except RTOG), 9, 15, 21, 30, 36, 42, 48, 54 & 60

[11]QL questionnaire: at baseline, post-neoadjuvant treatment, post ILND treatment, 12, 18, 24 and 36 months.

9. STUDY TREATMENT

Treatment of the primary tumour will usually be performed prior to entry into the study and will be by local excision or by partial/total penectomy, depending on stage of disease.

The pathway for any individual patient can involve as many as four interventions (the sequence: neoadjuvant chemotherapy; ILND; prophylactic PLND; adjuvant chemoradiotherapy) or as few as one (ILND), and the total duration of treatment is, therefore variable. The maximum duration of time on treatment for a given patient should be approximately 9 months and should not exceed 10 months unless this cannot be avoided.

9.1. Therapeutic options

Patients may receive between one and four study interventions:

- inguinal lymph node dissection (ILND);
- neoadjuvant chemotherapy (followed by ILND);
- synchronous chemoradiotherapy (in either the neoadjuvant or adjuvant setting);
- prophylactic pelvic lymph node dissection.

The sequence(s) of potential interventions are detailed in the following figure:

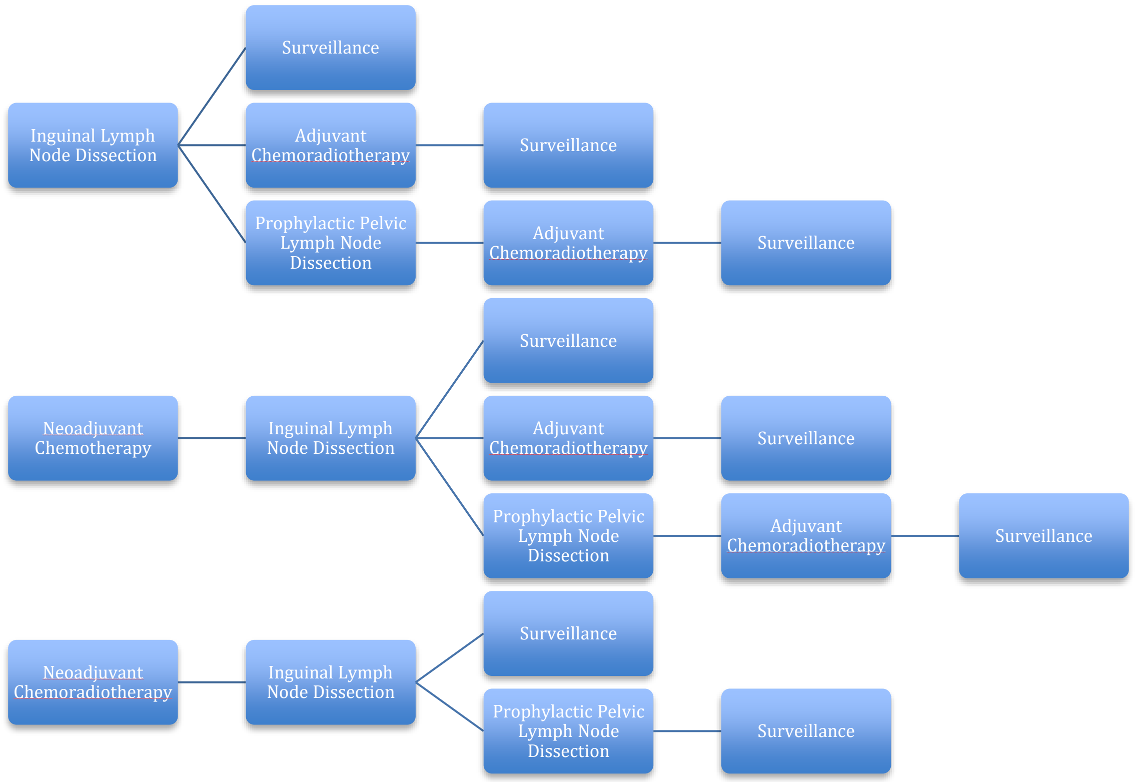

9.2. Inguinal lymph node dissection (ILND)

Inguinal procedures will address complete removal of all known disease by therapeutic inguinal lymph node dissection (ILND). ILND undertaken in the presence of macroscopic inguinal lymph node involvement is regarded as standard therapy, either alone or following neoadjuvant therapy.

Inguinal lymphadenectomy includes removal of the lymph nodes described by Daseler (see figure 1 in surgical section of trial guidance notes) between the area bordered medially by the midpoint of the adductor longus muscle, superomedially by the spermatic cord, reaching to 2-3cm above the inguinal ligament, laterally to the midpoint of the sartorious muscle with the inferior border being the apex of the femoral triangle. The floor of the dissection includes the skeletonized sartorious, pectineus, and adductor longus muscles and also includes the skeletonized femoral artery and vein (see figure 2 in surgical section of protocol guidance notes). Among low risk patients assigned to InPACT-neoadjuvant arm A, removal of the muscular fascia is not required and sparing the saphenous vein when feasible from an oncologic standpoint is encouraged.

ILND should be undertaken by a trained surgeon in accordance with the surgical section of the trial guidance notes (see also protocol section 12).

ILND should be undertaken as an open procedure utilizing skin incision of choice with the goal of minimizing morbidity. The surgical trial protocol subgroup will maintain a regular review of published data, and other approaches including laparoscopic/robotic approaches may be incorporated into a trial amendment at a later date.

If ipsilateral ILND is performed, simultaneous evaluation of the contralateral, clinically node-negative side via superficial inguinal lymphadenectomy (SILND) or dynamic sentinel node biopsy (DSNB) [19,20] is acceptable, based upon local site expertise. Options for surgical staging of the node-negative groin are discussed in the surgical section of the Surgical guidance notes.

9.3. Neoadjuvant chemotherapy

The investigational medicinal product (IMP) used for neoadjuvant chemotherapy is paclitaxel, ifosfamide and cisplatin (TIP). Normal hospital stock will be used for patients in the UK. For supply details of IMP for non-UK sites please see group specific appendix.

Patients randomised to arm B who have adequate GFR will receive systemic therapy as outlined below. Patients will receive up to 4 cycles of paclitaxel, ifosfamide, and cisplatin (TIP).

Chemotherapy will be administered in 21-day cycles. The first cycle should begin within 14 calendar days of randomisation. The planned duration of chemotherapy is 12 weeks.

Placement of an implantable infusion port, peripheral indwelling central catheter (PICC), or other central venous access device is permitted but not mandated.

Chemotherapy may be administered on an inpatient or an outpatient basis, to be determined by each treatment site. The treatment setting may vary between patients and between cycles for any given patient.

Patients will proceed to therapeutic inguinal lymph node dissection upon completion of chemotherapy. The date of ILND will be at least 6 weeks but not more than 12 weeks after day 1 of the last cycle of chemotherapy.

9.3.1. TIP chemotherapy regimen

Administration on an outpatient basis:

Day	Drug	Dose mg/m^2	Total Cycle Dose mg/m^2	Outpatient Regimen
**1**	paclitaxel	175 mg/m^2	175 mg/m^2	• Intravenous infusion over 3 hours. Paclitaxel infusion is preceded by premedication with steroid, anti-H1 anti-histamine and gastric acid suppression in accordance with local practice.
**2-5**	ifosfamide	900 mg/m^2	3600 mg/m^2	• Intravenous infusion over 1 hour with standard mesna uro-protection. The recommended regimen for mesna when given with the outpatient administration of TIP is mesna 400 mg/m^2 IV before ifosfamide, and 400 mg/m^2 PO at 2 and 6 hours after ifosfamide administration.
**1-5**	cisplatin	15 mg/m^2	75 mg/m^2	• Intravenous infusion over 1-2 hours as dictated by local policy. Cisplatin is given after paclitaxel (day 1) and before ifosfamide (days 2-5).

BSA should only be recalculated if body weight changes by greater than 10% compared to baseline. BSA should be calculated as per local practice.

Patients GFR should be 50ml/min or greater to receive TIP chemotherapy. GFR should be calculated using MDRD formula **AND** Cockcroft & Gault (see Appendix 3). Formal measurement of the GFR (by isotopic methods) is recommended where there is a discrepancy between these calculations that the investigator regards as clinically significant.

Steroid premedication, anti-emetic and hydration regimens are given in section 9.3.2. For information on disallowed concomitant medication please refer to the SmPC. All diluents/volumes used to give this chemotherapy regimen should be according to local practice.

Administration on an inpatient basis:

Day	Drug	Dose mg/m²	Total Cycle Dose mg/m²	Inpatient Regimen
**1**	paclitaxel	175 mg/m²	175 mg/m²	• Intravenous infusion over 3 hours. Paclitaxel infusion is preceded by premedication with steroid, anti-H1 antihistamine and gastric acid suppression in accordance with local practice.
**1-3**	ifosfamide	1200 mg/m²	3600 mg/m²	• Intravenous infusion over 2 hours with standard mesna uro-protection. The recommended regimen for mesna when given with the inpatient administration of TIP is mesna 1200 mg/m² IV on days 1 through 3, in three divided doses, 400 mg/m² IV before ifosfamide, and 400 mg/m² IV at 4 and 8 hours after ifosfamide administration.
**1-3**	cisplatin	25 mg/m²	75 mg/m²	• Intravenous infusion over 1-2 hours as dictated by local policy. Cisplatin is given after paclitaxel (day 1) and before ifosfamide (days 1-3).

BSA should only be recalculated if body weight changes by greater than 10% compared to baseline. BSA should be calculated as per local practice.

Patients' GFR should be 50ml/min or greater to receive TIP chemotherapy. GFR should be calculated using MDRD formula and Cockcroft & Gault (see Appendix 3). Formal measurement of the GFR is recommended where there is a discrepancy between these calculations that the investigator regards as clinically significant.

Steroid premedication, anti-emetic and hydration regimens are given in section 9.3.2. For information on disallowed concomitant medication please refer to the SmPC. All diluents/volumes used to give this chemotherapy regimen should be according to local practice.

9.3.2. Supportive medication

Growth Factor Support

Granulocyte Colony Stimulating Factor (G-CSF) may be used at the discretion of the clinician. Prophylactic G-CSF is encouraged in patients who have had episodes of febrile neutropenia. Use of G-CSF will be recorded in the medication record.

Anti-emetics

Anti-emetic medication for prophylaxis and treatment should be given according to the standard practice at each site.

Cisplatin hydration

Pre-hydration, infusion regimen and post-hydration should be according to local practice, but regimen details must be submitted to the relevant Data Centre as part of site initiation preparation. Pre- and post-hydration is a requirement for all patients receiving cisplatin, but the scheduling of hydration is subject to local practice. Examples of acceptable pre- and post-hydration schedules for this regimen are given in Appendix 1.

9.3.3. Dose banding and rounding

Chemotherapy doses may be dose-banded in line with the NHS England initiative, if it is routine local practice to do so (no other dose banding is permitted). The InPACT trial team must be informed of this during the site initiation process. Dose rounding to the nearest 1ml, to ease the draw up of doses in syringes in aseptic units is also acceptable.

9.3.4. Dose capping

There is no evidence of benefit for dose capping in patients with penile cancer. Those centres that routinely practice dose capping should not cap any dose at a BSA less than 2.25m^2 (i.e. any patient with a BSA of 2.25m^2 or less should be dosed at actual calculated BSA). BSA should be calculated according to local hospital policy. Those centres that do not routinely practice dose capping are encouraged not to adopt the practice for this trial.

9.3.5 Chemotherapy dose modifications/dose delays

A table of dose reductions is available in appendix 5 to the protocol. Appendix 5 lists the total dose to deliver over the whole cycle, ***not*** the daily dose. Please use the conversion table below to deliver the correct daily dose when referring to the dose reduction table. Please ensure the correct dose modification is used for each of the three agents; the table below is for conversion purposes only. Each chemotherapy agent should be delivered in line with either the outpatient or inpatient schedule as detailed in section 9.3.1.

**Drug**	**Full / modified dose**	**Total Cycle Dose (mg/m^2) as detailed in appendix 5**	**Daily Dose conversion (mg/m^2)**	
			**Outpatient**	**Inpatient**
paclitaxel	FULL	175 mg/m^2	175 mg/m^2 *(given day 1)*	175 mg/m^2 *(given day 1)*
	MODIFIED / REDUCED	140 mg/m^2	140 mg/m^2 *(given day 1)*	140 mg/m^2 (given day 1)
ifosfamide	FULL	3600 mg/m^2	900 mg/m^2 *(given days 2-5)*	1200 mg/m^2 *(given days 1-3)*
	MODIFIED / REDUCED	2880 mg/m^2	720 mg/m^2 *(given days 2-5)*	960 mg/m^2 *(given days 1-3)*
cisplatin	FULL	75 mg/m^2	15 mg/m^2 *(given days 1-5)*	25 mg/m^2 *(given days 1-3)*
	MODIFIED / REDUCED	60 mg/m^2	12 mg/m^2 *(given days 1-5)*	20 mg/m^2 *(given days 1-3)*

Neutropenia & Thrombocytopenia

Minimum haematological parameters to be met before commencing subsequent cycles are ANC> 1,500 mm^3 and platelets >75,000/mm^3. If these criteria are not met refer to dose modifications tables in Appendix 5. The general principle is that any delay of greater than 1 week will trigger a dose reduction in some or all of the chemotherapy drugs. The modifications listed should be regarded as the minimum safety requirements, and they do not invalidate the decisions of individual clinicians to further reduce doses or discontinue treatment.

Peripheral nerve toxicity

See section 9.3.6.

Nephrotoxicity

Treatment should be interrupted in the event of nephrotoxicity (defined as a fall in GFR to <50ml/min). Laboratory testing should be performed within 72 hours prior to the start of each chemotherapy cycle.

Calculated GFR ≥50 ml/min is required before starting each cycle.

Clinical judgement should supersede these parameters when nephrotoxicity is suspected e.g. in the event of creatinine elevation above the patient's baseline, but with other electrolyte disturbances compatible with deteriorating renal function.

Central Nervous System (CNS) toxicity / ifosfamide-induced encephalopathy

Patients exhibiting CNS symptoms (e.g., drowsiness, confusion, hallucinations) whilst receiving ifosfamide should be evaluated to exclude other possible causes. Ifosfamide infusion must be halted if ifosfamide-induced encephalopathy is suspected and supportive care given until symptoms have resolved. Treatment of ifosfamide-induced encephalopathy with methylene blue (50 mg as a single dose or every 4-8 hours until symptoms resolve) is permitted, but may not be necessary.

Patients may continue treatment on resolution of encephalopathy without dose reduction, at the discretion of the treating physician.

Hypersensitivity to paclitaxel

Patients who exhibit severe hypersensitivity reaction to paclitaxel (hypotension requiring treatment, dyspnoea requiring bronchodilator, angioedema, urticaria) will have the infusion stopped and will not be re-challenged.

Minor hypersensitivity reactions (flushing, tachycardia, mild hypotension, mild dyspnoea) are common and patients experiencing these may continue treatment without interruption.

9.3.6 Toxicity grading and criteria to discontinue chemotherapy

Treatment must be interrupted for non-hematologic toxicity of Grade 3 or worse. Treatment may resume when toxicity has resolved to ≤Grade 1. Chemotherapy doses on resumption will be as specified in Appendix 5. Where a Grade 3 non-haematological toxicity is not described in Appendix 5, all chemotherapy drugs will have their doses reduced by 20% on resumption.

Peripheral neuropathy

Peripheral neuropathy (PN) should be assessed according to its current extent at the time of the commencement of the next due cycle of chemotherapy.

Resolution to grade 1 should not be a cause for delay or dose reduction unless paraesthesia is having a negative impact upon the patient's quality of life.

Persistence of PN at grade 2 mandates a reduction in drug doses as described in Appendix 5; a delay in treatment is an acceptable alternative strategy, with resumption of treatment according to the above guidance if there is some resolution of symptoms.

The presence of PN grade 3 at the point of the next cycle of chemotherapy being due requires cessation of treatment until there has been resolution to grade 1 or better. Dose reductions as listed in Appendix 5 are mandatory.

Additional information on the safety and administration of these drugs can be found in their SmPC.

9.4. Chemoradiotherapy

Synchronous chemoradiotherapy with cisplatin may be delivered in the neoadjuvant and the adjuvant setting, subject to patient's treatment allocation, disease burden and GFR status.

Radiotherapy for InPACT patients is to be delivered with either a forward planned IMRT technique or inverse planned IMRT, performed using the local treatment planning system. Rotational arc therapies are permitted (Rapid Arc™, VMAT™ and Tomotherapy™).

Participation in the quality assurance programme provided by the National Radiotherapy Trials Quality Assurance group is mandatory for all UK sites. Please refer to section 11 for details of the QA programme.

Concurrent cisplatin 40mg/m^2 will be given weekly in accordance with local hospital protocols for delivery of synchronous chemotherapy and radiotherapy, subject to GFR≥45mls/min. The concurrent chemotherapy will be omitted if the GFR is less than this. Local hospital protocols should be followed for cisplatin dose modification following review of haematology/biochemistry and adverse events.

Patients in InPACT-neoadjuvant will proceed to therapeutic inguinal lymph node dissection upon completion of 5 weeks of chemoradiotherapy. The date of ILND is to be no more than 12 weeks after the last fraction of radiotherapy.

9.4.1. Target volume

Please refer to the current version of the Radiotherapy Planning, Delivery and Quality Assurance Guidelines for target volume delineation.

9.4.2. Dose

For neoadjuvant treatment, the radiotherapy dose is 45Gy in 25 fractions over 5 weeks using 6-10 MV photons to all regions.

For adjuvant treatment – full details on the technique is given in the planning guidance document, but note:

Groin: One or both groins may be boosted up to 54Gy in 25 fractions. An IMRT boost of up to 57Gy can be given to recurrent or residual macroscopic tumour

Pelvis: An IMRT boost of up to 54Gy in 25 fractions is applied to:

1. Any macroscopic tumour or pathological lymph nodes
2. Electively to external iliac nodes in patients with high disease burden

9.4.3. Duration of Treatment

Radiotherapy should commence within 4 weeks following randomisation for patients who need to commence neoadjuvant chemoradiotherapy and within 8 weeks post-operatively for adjuvant chemoradiotherapy (to allow sufficient time for planning). Chemoradiotherapy should be delivered in 25 fractions over 5 weeks.

Treatment scheduling and gaps: please see RT QA planning guidance document.

9.5. Pelvic lymph node dissection (PLND)

Pelvic procedures within this trial will primarily be performed on radiologically-uninvolved fields as a prophylactic procedure.

Where there are clinically-involved pelvic nodes, therapeutic PLND should be performed at the same time as ILND. Note: these patients are NOT eligible for InPACT-pelvis.

PLND should be undertaken by a trained surgeon in accordance with the surgical section of the trial guidance notes (see also protocol section 12).

If inguinal nodal disease is unilateral then ipsilateral PLND should be undertaken, whilst if inguinal nodal disease is bilateral, then bilateral PLND should be undertaken. Eligible patients who have radiologically-involved nodes should have all involved lymphatic tissue resected. In patients with unilateral clinically-involved pelvic node(s), performing a bilateral pelvic dissection (i.e. resecting the clinically negative side) is left to the discretion of the surgeon (see surgical guidance nodes).

The resection field should include all the ipsilateral lymphatic tissue medial to the genitofemoral nerve (between the genitofemoral nerve and the prostate, bladder, internal iliac artery, and the pelvic floor (3, 17), along the external iliac artery and vein (including the node of Cloquet), up to the bifurcation of the common iliac artery; within and below the obturator fossa / obturator nerve; and along the distal branches of the hypogastric vessels.

9.5.1. Prophylactic Pelvic lymph node dissection (prophylactic PLND)

Prophylactic PLND will be performed as a separate procedure after an inguinal lymphadenectomy in the majority of patients. It can be performed via an open (infra-umbilical) incision, by extraperitoneal dissection or by a minimally-invasive technique (robotic or laparoscopic). This decision is left to the discretion of the surgeon, based on clinical judgment and surgical experience.

9.5.2. Pelvic lymph node dissection (therapeutic PLND)

Resection of clinically-involved pelvic nodes should employ a standard open pelvic lymphadenectomy technique, as with the inguinal field. The surgical trial protocol subgroup will maintain a regular review of published data, and other approaches may be incorporated into a trial amendment at a later date.

10. CONCOMITANT THERAPY

All medication considered necessary for the participants' welfare and which is not expected to interfere with the evaluation of the study treatments may be given at the discretion of the investigator. All concomitant medications must be recorded in the patient's notes.

11. RADIOTHERAPY QUALITY ASSURANCE (RT QA)

The UK QA programme for the study will be co-ordinated by the National Radiotherapy Trials QA (RTTQA) Group.

The US QA programme for the study will be co-ordinated by the IROC Rhode Island QA Center.

UK centres must successfully complete the RTTQA group IMRT accreditation programme (or equivalent) in order to be approved to enter patients into the InPACT trial. An overview of the complete credentialing process, along with the associated data and documentation, is given on the RTTQA website (www.rttrialsqa.org.uk).

The IMRT accreditation programme for InPACT consists of 5 modules:

Pre-trial

- Facility questionnaire
- Outlining benchmark case
- Planning benchmark case
- Dosimetry audit

On-trial

- Prospective Case Reviews

All centres delivering radiotherapy for InPACT will need to submit the benchmark outlining and planning cases. The facility questionnaire will also need to be completed by all centres, although this document may be pre-filled according to the centre's participation in other pelvic trials.

The requirement for the dosimetry audit is determined on the centre's previous audit and trial participation. Centres may start entering and treating patients in the trial prior to the dosimetry audit (if applicable) being carried out, given successful completion of all other trial requirements. The dosimetry audit should be completed as soon as possible.

Centres are required to submit all cases for prospective case review. They must be approved by the QA team before patients can be treated.

Non-compliance with the study RT planning and delivery document will be reported to the CI and local PI and a decision regarding further site participation will be made.

12. SURGICAL QUALITY ASSURANCE

The requirements for surgical QA will be discussed within the relevant surgical trial guidance notes and group specific appendix.

UK patients are managed under the auspices of a supranetwork multidisciplinary team. These teams are expected to serve a population base of four million or more and expect to manage a minimum of 25 new patients each year. Discussion of all eligible patients by the relevant supranetwork MDT is mandatory.

13. PATIENT CARDS

Wallet-sized cards will be produced by ICR-CTSU for UK sites upon request by the participating site. Each card will state:

- the name of the participating site;
- that the patient is participating in the InPACT trial;
- that the patient is receiving paclitaxel, ifosfamide, cisplatin chemotherapy, OR that they are receiving cisplatin as part of their chemoradiotherapy;
- an emergency contact number.

14. PHARMACY RESPONSIBILITIES AND DRUG ACCOUNTABILITY

Non-UK sites should refer to group-specific appendix.

UK approvals for InPACT deem paclitaxel, ifosfamide and cisplatin (either alone or in combination) as Investigational Medicinal Products (IMPs). All IMPs should be prescribed by the investigator and dispensed from hospital pharmacy from hospital stock for the duration of the trial. All IMPs should be obtained from usual drug suppliers in accordance with local practice.

All dispensed IMPs are required to be labelled in accordance with the MHRA-approved InPACT label (see Trial Guidance notes) in addition to the local pharmacy label. Drug formulation, storage, accountability and destruction should be in accordance with local policy. ICR-CTSU should be provided with confirmation of the local pharmacy's clinical trial drug handling and destruction procedures. Further information on the IMPs in this study can be found in the Summary of Product Characteristics (SmPC) for each IMP.

15. PHARMACOVIGILANCE

15.1. Definitions

15.1.1. Adverse event (AE)

Any untoward medical occurrence in a patient or clinical trial subject administered a study treatment/investigational medicinal product (IMP); the event does not necessarily have a causal relationship with the treatment or usage.

15.1.2. Serious adverse event (SAE)

An SAE is any untoward medical occurrence that occurs after the commencement of study treatment and within 30 days of the last intervention and:

- results in death,
- is life-threatening
- requires hospitalisation or prolongation of existing inpatients´ hospitalisation
- results in persistent or significant disability or incapacity
- is a congenital anomaly or birth defect

Important adverse events that are not immediately life-threatening or do not result in death or hospitalisation but may jeopardise the subject or may require intervention to prevent one of the other outcomes listed in the definition above, may also be considered serious.

Progression of the indicated disease and death due to progression of the indicated disease are not considered SAEs.

15.1.3. Serious adverse reaction (SAR)

A serious adverse reaction is an SAE that is suspected as having a causal relationship to the study treatment/IMP, as assessed by the investigator responsible for the care of the patient. A suspected causal relationship is defined as possibly, probably or definitely related (see definitions of causality table).

Definitions of causality

Relationship	Description
Unrelated	There is no evidence of any causal relationship with the study treatment/IMP
Unlikely	There is little evidence to suggest there is a causal relationship (e.g. the event did not occur within a reasonable time after administration of the study treatment/IMP). There is another reasonable explanation for the event (e.g. the patient's clinical condition, other concomitant treatment)
Possible	There is some evidence to suggest a causal relationship (e.g. because the event occurs within a reasonable time after administration of the trial medication). However, the influence of other factors may have contributed to the event (e.g. the patient's clinical condition, other concomitant treatments)
Probable	There is evidence to suggest a causal relationship, and the influence of other factors is unlikely
Definitely	There is clear evidence to suggest a causal relationship, and other possible contributing factors can be ruled out
Not assessable	There is insufficient or incomplete evidence to make a clinical judgement of the causal relationship.

15.1.4. Suspected unexpected serious adverse reaction (SUSAR)

A serious adverse reaction following administration of an investigational medicinal product (cisplatin, ifosfamide or paclitaxel in this study), the nature or severity of which is not consistent with the approved Reference Safety Information (RSI) contained within the applicable Summary of Product Characteristics (SmPC), and is assessed as unexpected by the Chief Investigator or nominated representative.

15.1.5. Related Unexpected Serious Adverse Event

An adverse event, following the administration of non-investigational medicinal product treatment (radiotherapy only (without concurrent cisplatin) or surgery alone), that meets the definition of serious and is assessed by the CI or nominated representative as:

- "Related" – that is, it resulting from administration of any of the research procedures, and
- "Unexpected" – that is, the type of event is not listed in the protocol as an expected occurrence (see appendix 8).

15.2. UK reporting adverse events to ICR-CTSU

For non-UK reporting requirements please see relevant group specific appendix.

Any toxicity, sign or symptom that occurs after commencement of study treatment and within 30 days of the last administration of study treatment, which is not unequivocally due to progression of disease, should be considered an AE.

All AEs must be reported on the relevant CRF and forwarded to ICR-CTSU.

The severity of AEs should be graded according to the NCI-CTCAE criteria. For each toxicity/sign/symptom, the highest grade observed since the last visit should be reported.

Whenever one or more toxicity/sign/symptom corresponds to a disease or a well-defined syndrome only the main disease/syndrome should be reported.

15.3. UK reporting of serious adverse events to ICR-CTSU

Any SAE that occurs after the commencement of study treatment and up to 30 days following the last study treatment intervention must be reported.

All SAEs should be reported to ICR-CTSU within 24 hours of the Principal Investigator (or designated representative) becoming aware of the event, by completing the InPACT SAE form and sending to:

The ICR-CTSU safety desk
Email: **sae-icr@icr.ac.uk**
For the attention of the InPACT Trial team

As much information as possible, including the Principal Investigator's assessment of causality, must be reported to ICR-CTSU in the first instance. Additional follow up information should be reported as soon as it is available.

All SAE forms must be completed, signed and dated by the Principal Investigator or designated representative.

15.4. UK review of serious adverse events

The Chief Investigator (or designated representative) will assess all reported SAEs for causality and expectedness (NB. The Chief Investigator cannot downgrade the Principal Investigator's assessment of causality.)

SAEs assessed as having a causal relationship to the study treatment/IMP and as being unexpected (SUSARs) will undergo expedited reporting to the relevant authorities by ICR-CTSU (see figure 3 for SAE reporting).

Sites should respond as soon as possible to requests from the Chief Investigator or designated representative (via ICR-CTSU) for further information that may be required for final assessment of an SAE.

15.5. UK expedited reporting of SUSARs

If an SAE, following administration of an investigational medicinal product (cisplatin, ifosfamide or paclitaxel in this study), is identified as being a SUSAR by the Chief Investigator, and is fatal or life threatening, it will be reported by ICR-CTSU to the MHRA, the main REC, the Sponsor(s) within 7 days of being notified of the event.

If an SAE is identified as a SUSAR by the Chief Investigator, and is not fatal or life threatening, it will be reported by ICR-CTSU to the MHRA, the main REC, within 15 days of ICR-CTSU being notified of the event.
ICR-CTSU will report any additional relevant information to the MHRA, main REC, and as soon as possible, or within 8 days of the initial report of a fatal/life threatening SUSAR.

The Principal Investigators at all actively recruiting sites will be informed at regular intervals of any SUSARs occurring within the trial.

15.6. UK expedited reporting of Related Unexpected SAEs

If an SAE, following the administration of non-investigational medicinal product treatment (radiotherapy only (without concurrent cisplatin) or surgery alone), is identified as being related and unexpected by the Chief Investigator (or nominated representative) it will be reported by ICR-CTSU to the main REC, the Sponsor and all other interested parties within 15 days of notification.

The Principal Investigators at all actively recruiting sites will be informed at appropriate intervals of any related unexpected SAEs occurring within the trial.

15.7. UK follow up of serious adverse events

SAEs should be followed up until clinical recovery is complete or until disease has stabilised. SAE outcomes should be reported to ICR-CTSU using the relevant section of the SAE form as soon as the Principal Investigator becomes aware of the outcome.

15.8. UK annual reporting of serious adverse reactions

An annual report will be provided to the MHRA and the main REC by ICR-CTSU at the end of the reporting year.

15.9. Reporting pregnancies

If any trial participants’ partner becomes pregnant during or up to 6 months after that patient’s study treatment, this should be reported to ICR-CTSU using the pregnancy reporting form. Partner pregnancies should be followed to conclusion and all follow-up information should be reported to ICR-CTSU. If the outcome of the pregnancy meets the definition of serious (i.e. congenital abnormality) this should be reported to ICR-CTSU following the serious adverse event reporting procedures described above. Please refer to section 5.7 of the protocol for lifestyle guidelines and use of effective double-barrier contraception.

Figure 3: Flow diagram for UK SAE reporting, and action following report (for events following administration of an investigational medicinal product (cisplatin, ifosfamide or paclitaxel in this study)

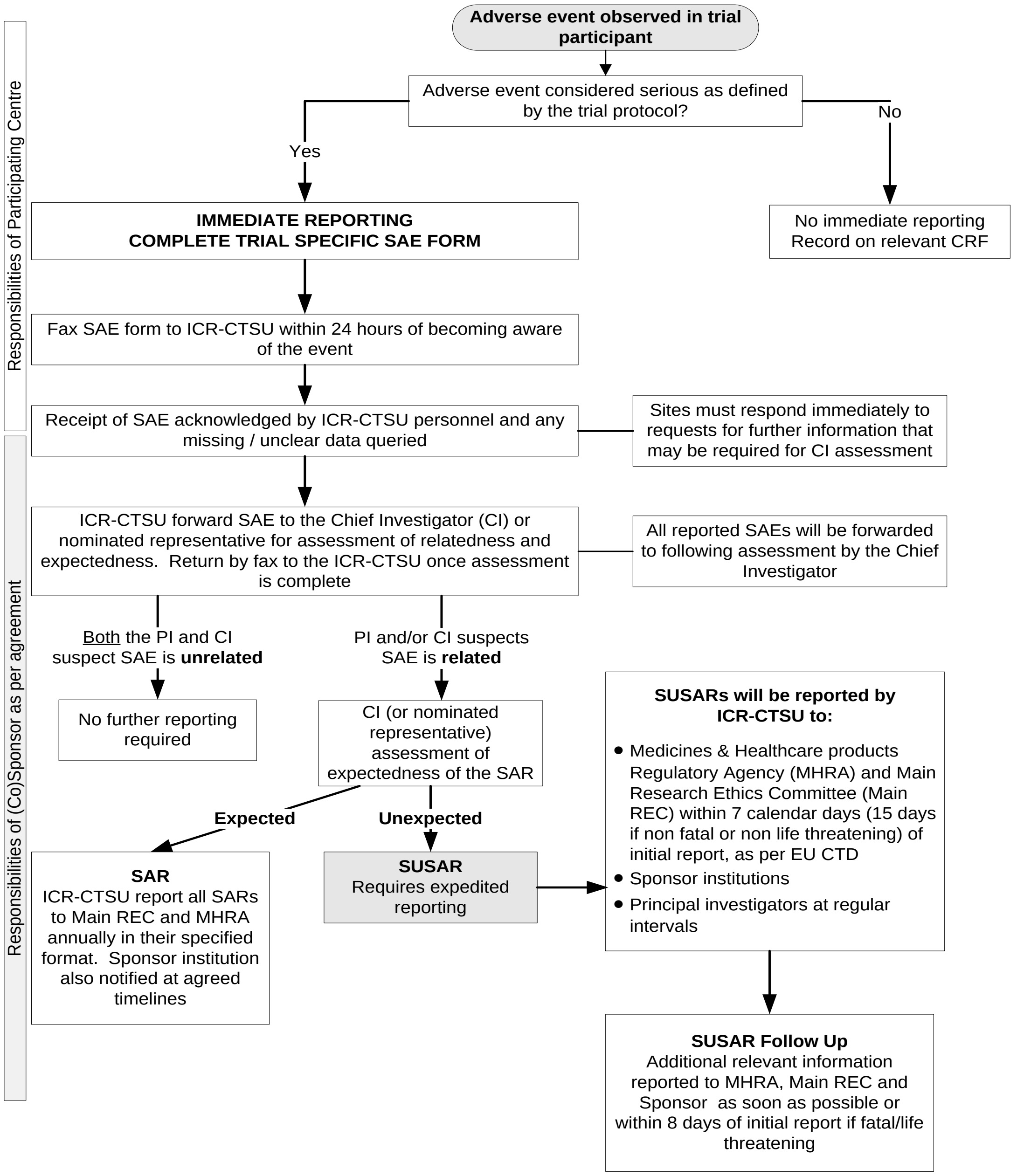

NB. All SAEs should continue to be followed up as specified above

Figure 4: Flow diagram for UK SAE reporting, and action following report (for events following the administration of non-investigational medicinal product treatment (radiotherapy only (without concurrent cisplatin) or surgery alone

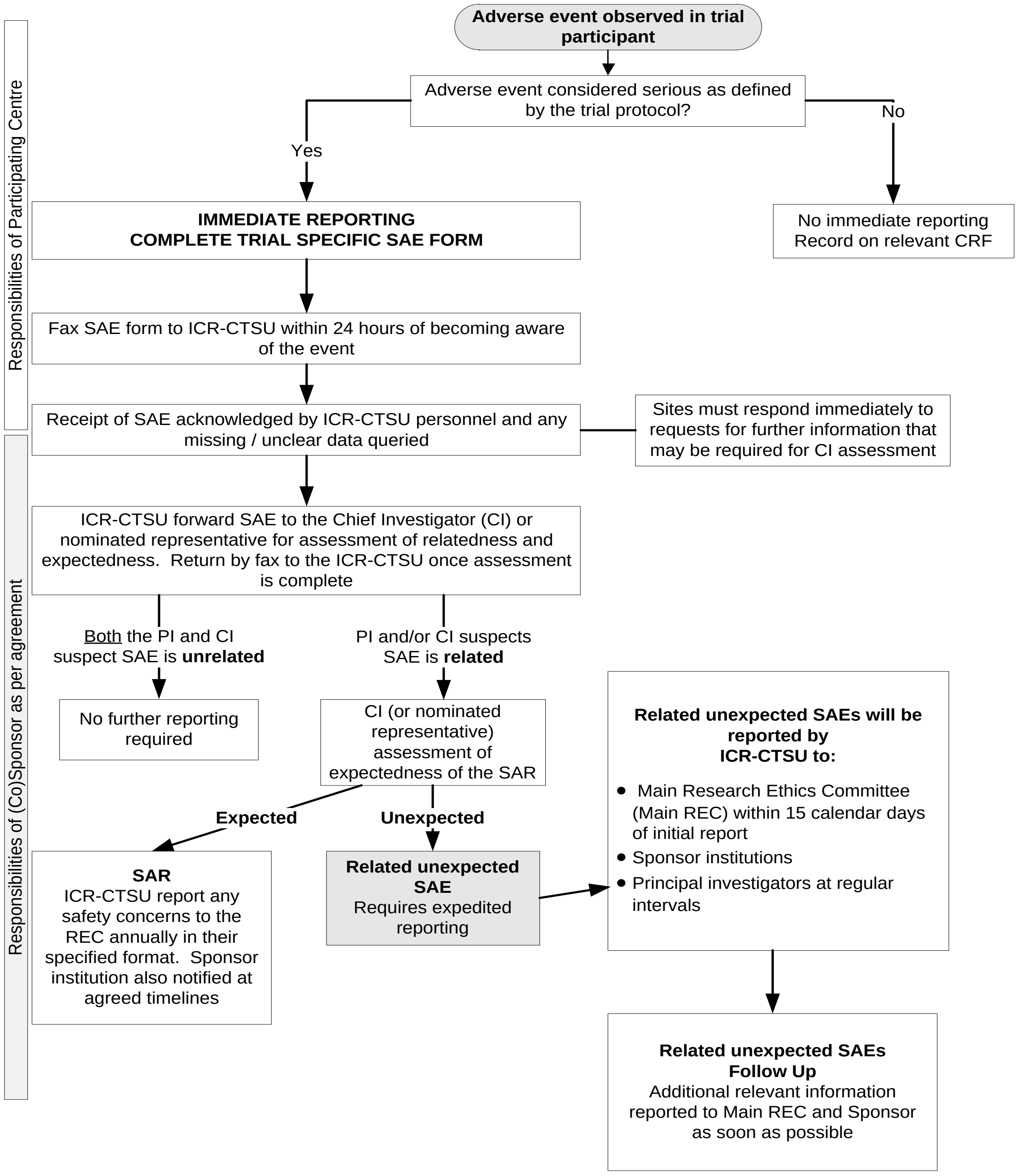

NB. All SAEs should continue to be followed up as specified above

16. STATISTICAL CONSIDERATIONS

16.1. Trial design

The trial design aims to evaluate the difference between treatments in terms of clinical outcomes at specific points along the patient pathway. The statistical analysis will primarily take a Bayesian approach based on a proposed philosophy for trials in rare diseases [21]. The aim is to collect prospective unbiased randomised trial data in as many patients as feasible and, with the focus on estimation rather than testing hypotheses, use these data to reduce the uncertainty about the size of treatment effects. The primary outcome measure for the trial is survival time and the log of the hazard ratio (HR) is the primary measure of treatment effect.

There were 558 new cases of penis cancer and 111 deaths in the UK in 2011 [1]. The annual UK patient pool for InPACT is estimated to be 400 patients and of these approximately 40% (160) are anticipated to be eligible [22,23]. Cautiously assuming that 50% of patients are approached and that 50% of these accept entry to the study suggests that 40 UK patients per annum would enter the study giving 200 UK patients over 5 years.

There were 1570 new cases of penis cancer and 310 deaths in the US in 2013 [2]. Assuming 5% of patients are approached (78) and 50% of those approached consent to participate (39), there would be 195 patients enrolled from US sites over 5 years.

The original recruitment target was 400 patients. Following a formal proposal to reduce the sample size due to low recruitment, the Trial Steering Committee reviewed the operating characteristics data in line with accrual and agreed that the protocol should be amended to increase the planned recruitment period to 7 years, keeping the same minimum follow-up period of 2 years and reduce the overall target sample size from 400 to 200 patients.

From these 200 patients, it is estimated that approximately:

- 67% of the total number of patients accrued will have high or intermediate disease burden and be included in InPACT-neoadjuvant (Randomisation 1)

There are three main assumptions that are used to predict the flow of the patients that are within the trial:

1. The stratification of patients into the low, intermediate and high burden of disease is expected to be in the ratio of 1:1:1
2. The stratification of patients into good and poor GFR (i.e. > or <55mls/min respectively) is expected to be in the ratio of 2:1
3. The post-surgery pathological high risk of relapse rates are related to the original disease burden at diagnosis with rates of 33%, 50% and 100% for low, intermediate and high disease burden respectively.

These assumptions can then be used to estimate the number of patients that will be entering the trial and the table below gives the estimated number of patients and number of events available for each comparison of interest (further details on the derivation of these numbers is given in the Statistical Analysis Plan). Estimation of the number of events [24] assumes 7 years recruitment and 2 years minimum follow-up and assumes 5-year survival rates of 50% and 15% respectively in patients with intermediate and high disease burden in Randomisation 1 and of 15% in high-risk patients in Randomisation 2.

Randomisation	Comparison	Estimated number of patients accrued	Estimated number of events observed
InPACT-neoadjuvant: Randomisation 1	Q1a surgery alone (arm A) vs neoadjuvant therapy (arms B&C)	88	49
InPACT-neoadjuvant: Randomisation 1	Q1b neoadjuvant chemotherapy (arm B) vs neoadjuvant chemoradiotherapy (arm C)	66	46
InPACT-pelvis: Randomisation 2	Q2 PLND (arm P) vs no PLND (arm Q)	120	97

Using a reverse philosophy to that for standard sample size calculations, the trial design is evaluated by considering the utility of the information that will be provided by the estimated number of events for each randomised comparison shown in the table above. Evaluation of the design is based on the proposed statistical analysis plan for the primary outcome as specified in Section 16.4 but uses a normal-normal conjugate analysis with non-informative, sceptical and enthusiastic off-the-shelf priors. Full details of the evaluation are given in the Statistical Analysis Plan with some examples of the utility given here. The number of events will provide sufficient data for the posterior distributions to show on average that the treatment has a beneficial effect on survival (i.e. hazard ratio (HR) < 1) assuming a modest treatment effect is observed (i.e. HR=0.8). Given a decision criteria that a treatment would be accepted if the posterior distribution shows at least a 60% chance that the true HR<1, there is a high probability (70% for Q1a, 69% for Q1b and 80% for Q2) of selecting the right treatment using this Bayesian decision rule if a treatment has a true modest clinically relevant effect (i.e. HR=0.8) and a low probability (14% for Q1a, 15% for Q1b and 8% for Q2) of incorrectly accepting a treatment when there is a true negative effect (i.e. HR=1.25). There is a moderately high chance (40% for all 3 questions) of incorrectly selecting the experimental treatment for future use if there is no true effect (HR=1); this is considered an acceptable trade-off.

For the purposes of estimating the number of patients recruited and the number of events observed, it is assumed that all patients entering the study do so in accordance with the preferred randomisation. As described in table 2, it is anticipated that a small number of patients might “elect” surgery (A) or neoadjuvant chemoradiotherapy (C) in a non-randomised fashion. If it transpires that a significant proportion of patients with intermediate or high burden of disease fall into the non-randomised cohort the TMG will consider the impact on the sample size considerations and seek independent advice from the IDMC and/or TSC on the target sample size.

16.2. Treatment Allocation

16.2.1. InPACT-neoadjuvant – Randomisation 1

Patients with an intermediate or high disease burden (based on radiological criteria defined earlier) are eligible to be randomised to neoadjuvant therapy. Those with two radiologically-involved nodes (see table in section 5.5) will be randomised between surgery (arm A) and neoadjuvant therapy (arm B or arm C) in a 2:1:1 ratio; whereas the preferred randomisation for those with radiological high-burden disease (3 or more involved nodes in an inguinal basin, or extra-nodal extension, or pelvic node involvement) will be between neoadjuvant chemotherapy (arm B) and neoadjuvant chemoradiotherapy (arm C). Randomisation is carried out independently by the co-ordinating trials units (ICR-CTSU and ECOG-ACRIN). Treatment allocation is by minimisation with a random element;

balancing factors will be country (e.g. UK, other EU) and laterality of disease (for the high risk disease burden group only).

16.2.2. InPACT-pelvis – Randomisation 2

Any patient whose groin dissection reveals either extra-nodal extension or 3 or more involved nodes should be considered at high risk of harbouring occult micrometastatic disease in the ipsilateral pelvic nodes. These patients are eligible for randomisation 2. InPACT-pelvis patients who have not received chemoradiotherapy in the neoadjuvant setting will receive PLND followed by adjuvant chemoradiotherapy (arm P) OR adjuvant chemoradiotherapy alone (arm Q). Those patients who have already undergone chemoradiotherapy in the neoadjuvant setting will be randomised to PLND (arm P) or surveillance (arm Q).

Randomisation is carried out independently by the co-ordinating trials units (ICR-CTSU and ECOG-ACRIN). Treatment allocation is by minimisation with a random element; balancing factors will be country (e.g. UK, other EU), extracapsular spread, neoadjuvant treatment and laterality of disease (for the high risk disease burden group only).

16.3. Outcome measure definitions

16.3.1. Primary outcome measure

The primary outcome measure for all patients is *survival time*. This is defined as the whole days that have elapsed from the date of randomisation to the date of death from any cause. Survival time will be censored at the date of last follow-up for those who have not been reported as dead at the time of analysis.

16.3.2. Secondary outcome measures

Secondary outcome measures that will be measured for all patients are:

- *Disease-specific survival time,* which is defined as whole days that have elapsed from the date of randomisation to the date of death specifically from penis cancer. Survival time will be censored at the date of last follow-up for those who have not been reported as dead at the time of analysis. Where death is not reported as being due to penis cancer, the survival time will be censored at date of death.
- *Disease-free survival time (DFS),* which is defined as whole days that have elapsed from date of randomisation to the date of either locoregional recurrence, distant metastasis or death from penis cancer, whichever occurs first. DFS time will be censored at the date last known to be alive and free of disease, or at the date of death from other causes, for those who have not been reported as experiencing any of these events. A supplementary exploratory outcome measure will also be calculated taking date of primary penile surgery as the origin rather than date of randomisation.
    - A subsidiary outcome measure will be *locoregional recurrence free survival time (LRFST),* which is defined as whole days that have elapsed from date of randomisation to the date of locoregional recurrence. LRFST will be censored at the date last known to be alive and free of disease for those who have not been reported with this event.
    - A subsidiary outcome measure will be *distant metastases free survival time (DMFST),* which is defined as whole days that have elapsed from date of randomisation to the date of distant metastasis or death from disease, whichever occurs first. DMFST will be censored at the date

last known to be alive and free of distant metastasis or date of death from other causes for those who have not been reported as experiencing either of these events.
  - A supplementary exploratory outcome measure will also be calculated taking date of primary penile surgery as the origin for all these outcome measures rather than date of randomisation.
- *Toxicity* where all events experienced by patients are recorded and graded using CTCAE Version 4 criteria and specifically the occurrence of at least one grade 3 or 4 event
- *Occurrence of surgical complication,* which is recorded as whether or not a surgical complication was experienced according to the Modified Clavien-Dindo Classification criteria.
- *Feasibility of pathological nodal assessment after chemotherapy,* which is recorded as whether or not it was possible to achieve a pathological nodal assessment after chemotherapy.
- *Quality of life* (in participating patients) which is measured using the EORTC-QLQC30 and Lymphodema-QL on 8 occasions - prior to randomisation, 3-monthly during year 1, 6-monthly during year 2 and at the end of year 3 (see Appendix 4 for further detail).

Secondary outcome measures that will be measured for all trial patients in InPACT-neoadjuvant are:
- *Occurrence of pathological complete remission,* which is defined as complete absence of disease on histological examination in accordance with the guidelines of the Royal College of Pathologists.
- *Operability,* which will be recorded as whether or not the planned inguinal node dissection was undertaken and the reasons if it did not.
- Feasibility of on-schedule delivery of neoadjuvant therapy.

Secondary outcome measures for all trial patients in InPACT-pelvis are:
- Occurrence of Lower limb/scrotal oedema, which is recorded according to CTCAE Version 4 criteria.

In addition to the outcome measures, the acceptability of both randomisations will be monitored based on the proportion of eligible patients approached for randomisation, the proportion of approached patients consenting to randomisation and the proportion of randomised patients receiving their allocated treatment. The proportion of patients entered into InPACT-neoadjuvant with intermediate or high burden of disease who do not undergo the preferred randomisation/treatment allocation as defined in table 2 will also be monitored. This will provide ongoing information regarding the feasibility of the trial successfully completing to target with a planned milestone review of the feasibility of recruitment after 2.5 years.

16.4. Statistical analysis plan

Data from all collaborative groups will be pooled for central analysis to be undertaken at ICR-CTSU. The Statistical Analysis Plan will include a section to detail how data impacted by strategies introduced to mitigate risks relating to the COVID19 pandemic will be handled.

The primary aim of the statistical analysis is to compare, for each of the three questions, the ‘experimental’ treatment to the ‘control’ treatment in terms of the effect on survival time.

Patients randomised to options where surgery is a comparator (i.e. randomised to A v B v C or randomised to A v C) will be included in comparisons between surgery and neoadjuvant therapy (Q1a). Analyses will be stratified by randomisation option/disease burden.

Only patients randomly allocated to one of the two neoadjuvant treatment strategies (i.e. randomised to A v B v C or randomised to B v C) will contribute to the randomised comparison of neoadjuvant chemotherapy and neoadjuvant chemoradiotherapy (Q1b). Analyses will be stratified by randomisation option/disease burden.

For those that enter InPACT-pelvis, comparisons will be made between those randomised to receive PLND versus those randomised to not receive PLND (Q2). Analyses will be stratified by presence of extracapsular spread and whether the patient received neoadjuvant chemoradiotherapy.

The primary comparative analysis of survival time and other time-to-event endpoints will be based on the intention-to-treat principle including all patients randomised. The hazard ratio (HR) is chosen as the measure to reflect the treatment effect. It is more appropriate to focus the analysis on estimation rather than hypothesis testing when investigating rarer cancers, and a Bayesian approach to estimation will be used as recommended for rare diseases.

Conjugate analysis will be used to calculate the posterior distribution for the HR, and the primary analysis will be based on non-informative priors to give a likelihood-based Bayesian analysis and to enable the results to wholly reflect the unbiased data collected in the trial. Evidence-based priors will be generated and used in a supplementary analysis. Posterior probability distributions will be estimated for the treatment effects in terms of the primary and secondary outcomes. Means and standard deviations from these distributions will give estimates of the true treatments effect and the standard errors. The posterior distributions will give probabilities that the treatment effect is at least a certain pre-specified clinically relevant size, in particular hazard ratios of 1 and 0.8 for the primary outcome of survival time.

Descriptive analysis will include Kaplan-Meier estimates of event rates curves for all time-to-event data. The Cox proportional hazard model will be used to adjust for stratification factors and HPV status in the estimation of treatment effects. Methods to account for non-proportionality will be used if appropriate. The principle time-point of interest for LRFST in InPACT-pelvic (randomisation 2) is 2 years.

Analysis of pathological complete remission will include any patient who completes at least 2 cycles of TIP.

Analysis of safety by treatment received will be conducted on the safety population, defined as all patients who commence trial treatment. Toxicity will be summarised by the proportions experiencing grade ≥3 side effects. In addition, methods for ordinal data may be used. Standard algorithms will be used to derive scores from (and handle data missing from) QL questionnaires. Treatment groups will be compared at individual time-points, and analyses to account for the longitudinal nature of the data (e.g. generalised estimating equations or mixed effects models) may be used.

It is likely that data on some of the secondary endpoints (e.g. response rate to neoadjuvant therapy, toxicity) will mature ahead of data on overall survival. The Independent Data Monitoring and Trial Steering Committees will be required to give permission for results relating to secondary endpoints to be published prior to results on overall survival being available.

Feasibility of recruitment will be assessed against recruitment milestones using descriptive methods and will be monitored by the InPACT Executive Committee (IEC), Trial Steering Committee (TSC) and Independent Data Monitoring Committee (IDMC) throughout the duration of the trial (see section 16.5 below). Compliance with neoadjuvant treatment will be summarised by the number of cycles received. Dose delays and dose reductions will also be reported.

Exploratory subgroup analysis is planned to investigate any potential differential treatment effects linked to baseline characteristics, including HPV status, and to prior treatments received. It is acknowledged that the sample size may be small for some of these subsets, and thus cautious interpretation will be required

Sensitivity to assumptions and analysis population restrictions will be explored. In InPACT-neoadjuvant, data from non-randomised low disease burden patients and any intermediate/high disease burden patients who elect treatment (rather than undergo randomisation) will not contribute to the primary analysis of the randomised comparisons. Data from these non-randomised cohorts will provide useful supportive descriptive evidence which may be incorporated in secondary modelling.

Further details of analysis methods and analysis populations will be specified in a Statistical Analysis Plan in accordance with ICR-CTSU Standard Operating Procedures.

16.5. Interim analyses and stopping rules

There are no formal stopping rules for efficacy. An independent data monitoring committee (IDMC) (see section 17.4) will review the accumulating data at regular intervals with an initial review planned after 40 patients or 1 year of accrual (whichever is sooner).

Futility: Each randomised comparison will be monitored for futility using a form of the LIB20 rule proposed by Freidlin et al[28]. Specifically, starting when half the expected deaths for a given comparison have occurred and continuing annually thereafter (or after a 10% increment in the information proportion, whichever comes last), if the estimated treatment effect shows that the experimental treatment is worse than the standard treatment (Hazard ratio > 1), the Independent Data Monitoring Committee will consider recommending that the randomised comparison be stopped and remaining patients treated with the standard treatment. If the question 1A comparison of neoadjuvant treatment vs. none for intermediate risk patients stops for futility, power for question 1B may need to be reassessed, but could potentially continue for high risk patients only.

Safety: Serious adverse events will be subject to real-time safety review by the TMG. A safety “threshold” will be set and if serious adverse event rates exceed this threshold or there are any other concerns these will be escalated to the IDMC who would make recommendations about the continuation or modification to the trial design in light of safety and emerging efficacy (including pCR rate) data.

Safety: Surgical quality – If, after 50 patients have had ILND on either continent are enrolled, the rate of visibly incomplete dissection for that continent exceeds 10%, the IDMC may temporarily suspend recruitment to the study on that continent pending a safety review.

Feasibility of recruitment: Recruitment will be monitored in accordance with NCI slow accrual rules. A specific recruitment milestone review will take place by the 5th quarter following activation of the study. If by the 5th quarter the study is not accruing at 20% of the expected rate, or by the 8th quarter is not accruing at 50% of the expected rate, the IDMC may recommend redesign or closure in discussion with the independent Trial Steering Committee. Any decision to close the study (or a component of the study) on the basis of poor recruitment will give due consideration to the additional value that further recruitment (for up to a maximum total recruitment period of 5 years) would have in terms of contributing further randomised data to the evidence base in this rare cancer setting under a Bayesian analysis framework. Consideration will be given to each trial question (Q1a, Q1b, Q2) in turn. If there is limited or slow recruitment to once randomisation option, consideration will be given to amending the study design to drop that particular randomisation option. Conversely, if there is a need to increase recruitment to InPACT-pelvis, direct entry to this component of the study (Q2) may also be considered by amendment if this is supported by the independent Trial Steering Committee.

The InPACT Executive Committee recognised that as of April 2022 recruitment was behind target. The majority of the patients had been allocated to the comparison of neoadjuvant chemotherapy with neoadjuvant chemoradiotherapy (Arm B vs Arm C; question 1b). The oversight committees agreed that recruitment to all arms should continue until a target of at least 66 patients (with number of required events) for the B vs C randomisation (question 1b) is met, or for a further 2 years (whichever is longer). Recruitment will continue to all arms to gather as much information as possible to add to the overall body of evidence. It is recognised that the trial may not have sufficient power to answer the other research questions posed at the outset of the trial, due to the reduced number of patients. The IEC, IDMC and TSC will review recruitment and any emerging data and advise on continued recruitment.

17. TRIAL OVERSIGHT

17.1. Executive committee

An InPACT executive committee (IEC) will be established and will be chaired by the Study Chair (SN). Members will include the US co-chair (CP), Deputy Director of ICR-CTSU (EH), a senior representative and/or project manager from ICR-CTSU and ECOG-ACRIN and the chemotherapy, surgical, chemoradiotherapy, imaging, pathology and statistical subgroup chairs. A lay/consumer member will be sought. A representative from the ICRI partnership, not otherwise involved in the day to day running of the trial may be invited to attend meetings of the executive committee.

The executive committee will meet at regular intervals, and at least annually. Notwithstanding the legal obligations of the Sponsors and Chief Investigator, the executive committee have responsibility for the conduct of the trial.

17.2. Trial Management Group (TMG)

Country/collaborative group specific Trial Management Groups (TMGs) may be set up to be chaired by the Study Chair/US Co-chair or country specific clinical lead. These TMGs will have day to day responsibility for the smooth running of the trial within their country/collaborative group.

In the UK, the TMG will be chaired by the Study Chair (SN) and will include grant co-investigators and identified collaborators, the trial statistician and trial manager. Principal Investigators from participating sites and key study personnel will be invited to join the TMG as appropriate to ensure representation from a range of sites and professional groups. The TMG will meet at regular intervals, and at least annually. Notwithstanding the legal obligations of the Sponsors and UK Chief

Investigator, the UK TMG will have operational responsibility for the conduct of the trial in the UK. The UK TMG's terms of reference, roles and responsibilities will be defined in a charter issued by ICR-CTSU.

17.3. Trial Steering Committee (TSC)

An international Trial Steering Committee (TSC) will be set up and will include an independent Chairman (not involved directly in the trial other than as a member of the TSC) and not less than two other independent members. The TSC will meet at least annually. The TSC will provide expert independent oversight of the trial on behalf of Sponsor and funders. The Committee's terms of reference, roles and responsibilities will be defined in charter issued by ICR-CTSU. Selected members of the Executive Committee will be invited to attend meetings of the TSC.

17.4. Independent Data Monitoring Committee (IDMC)

An international IDMC will be instigated to monitor the progress of the trial. Membership of the IDMC will be proposed by the Executive Committee and approved by the international TSC. The Committee's terms of reference, roles and responsibilities will be defined in a charter issued by ICR-CTSU.

The IDMC will meet in confidence at regular intervals, and at least annually. A summary of findings and any recommendations will be produced following each meeting. This summary will be submitted to the Executive Committee and TSC, and REC/IRB and regulatory bodies (e.g. MHRA) as required.

The IDMC reserve the right to release any data on outcome or side effects through the TSC to the Executive Committee and TMG(s) (and if appropriate to investigators or participants) if it determines at any stage that the combined evidence from this and other studies justifies it.

18. RESEARCH GOVERNANCE

18.1. Sponsor responsibilities

The sponsor of this clinical trial in the UK is the Institute of Cancer Research (ICR). In the US, South America and Canada the Sponsor is the NCI.

18.2. Participating site responsibilities

Responsibilities delegated to participating sites are defined in an agreement between the sponsor and the individual site.

19. TRIAL ADMINISTRATION AND LOGISTICS

19.1. Site activation

Before activating the trial, participating sites are required to sign an agreement accepting responsibility for all trial activity which takes place within their site.

Sites may commence recruitment once the site agreement has been signed by all required signatories, the required trial documentation is in place (as specified by the Data Centre) and a site initiation (visit or teleconference) has taken place. Site initiation visits will be conducted at sites where the Principal Investigator has requested one or where the Data Centre deems it is appropriate.

19.2. Data acquisition

Electronic (e) Case Report Forms (CRF) will be used for the collection of trial data. The Data Centre will provide guidance to sites to aid the completion of the eCRFs. The Trial Management Group reserves the right to amend or add to the eCRF template as appropriate. Such changes do not constitute a protocol amendment, and revised or additional forms should be used by sites in accordance with the guidelines provided by the Data Centre.

UK-led sites will enter data using MACRO™ and US-led sites will enter data using Medidata Rave®.

Data collected by collaborative groups, including that collected in the parallel protocol in the US (EA8134), will be pooled for analysis at the ICR-CTSU.

19.3. Central data monitoring

Once data has been entered on the eCRF by the site personnel, the Data Centre will review it for compliance with the protocol, and for inconsistent or missing data. Should any missing data or data anomalies be found, queries will be raised for resolution by the site.

Any systematic inconsistencies identified through central data monitoring may trigger an on-site monitoring visit.

19.4. On-site monitoring

If a monitoring visit is required, the Data Centre will contact the site to arrange the visit. Once a date has been confirmed, the site should ensure that full patient notes of participants selected for source data verification are available for monitoring.

Data Centre staff conducting on-site monitoring will review essential documentation and carry out source data verification to confirm compliance with the clinical trial agreement and trial protocol. If any problems are detected during the course of the monitoring visit, the Data Centre will work with the Principal Investigator or delegated individual to resolve issues and determine appropriate action.

19.5. Completion of the study and definition of study end date

The study end date is deemed to be the date of last data capture.

19.6. Archiving

Essential trial documents should be retained according to local policy and for a sufficient period for possible inspection by the regulatory authorities (at least 5 years after the date of last data capture). Documents should be securely stored and access restricted to authorised personnel.

20. PATIENT PROTECTION AND ETHICAL CONSIDERATIONS

This section applies to UK sites only for non–UK sites please refer to group specific appendix.

20.1. Trial approvals

This trial has been formally assessed for risk by ICR-CTSU.

The trial has received ethical approval from a research ethics committee for multi-centre trials, regulatory approval from the MHRA and global R&D approval via the NIHR Coordinated System for gaining NHS Permission. Before entering patients, the Principal Investigator at each site is responsible for obtaining local approvals.

20.2. Trial conduct

This trial will be conducted according to the approved protocol and its amendments, supplementary guidance and manuals supplied by the Sponsor and in accordance with The Medicines for Human Use (Clinical Trials) Regulations 2004 as amended, the Research Governance Framework for Health and Social Care and the principles of GCP.

20.3. Informed consent

Patients should be asked to sign the current main REC approved InPACT consent form at trial entry after receiving both verbal and written information about the trial, having been given sufficient time to consider this information. All consent forms must be countersigned by the Principal Investigator or a designated individual. A signature log of delegated responsibilities, listing the designated individuals and the circumstances under which they may countersign consent forms, must be maintained at the participating site. This log, together with original copies of all signed patient consent forms, should be retained in the Site Investigator File and must be available for inspection. The current main REC approved InPACT patient information sheets should be provided in addition to any standard patient information sheets that are provided by the site and used in routine practice.

20.4. Patient confidentiality

Patients will be asked to consent to their full name being collected at registration in addition to their date of birth, hospital number, postcode and NHS number or equivalent to allow linkage with routinely collected NHS data and ensure accuracy in handling biological samples.

Each investigator should keep a separate log of all participants' unique Trial Identification IDs, names, addresses and hospital numbers. The investigator must retain trial documents (e.g. participants' written consent forms) in strict confidence. The investigator must ensure the participants' confidentiality is maintained at all times.

Representatives of ICR-CTSU and the regulatory authorities will require access to participants' hospital notes for quality assurance purposes. ICR-CTSU will maintain the confidentiality of participants at all times and will not reproduce or disclose any information by which participants could be identified.

20.5. Data Protection

All investigators and trial staff must comply with applicable data protection laws at all times.

20.6. Liability

In the UK, indemnity to meet the potential legal liability of investigators participating in this trial is provided by the usual NHS indemnity arrangements.

21. FINANCIAL MATTERS

This section applies to UK funding, please refer to relevant group specific appendix for non-UK wording.

This trial is investigator designed and led and has been approved by the Clinical Trials Advisory & Awards Committee (CTAAC) of Cancer Research UK.

ICR has received funding from Cancer Research UK for the central coordination of the trial. In the UK, the trial meets the criteria for R&D support as outlined in the Statement of Partnership on Non-Commercial R&D in the NHS in England. The trial is part of the National Institute for Health Research

Clinical Research Network (NCRN) portfolio by virtue of its approval by CTAAC. NCRN resources should therefore be made available for the trial to cover UK specific research costs.

22. PUBLICATION POLICY

Trial results will be published in peer-reviewed journals on behalf of all collaborators. Manuscripts will be prepared by a writing group, consisting of members of the Executive Committee and selected TMG members. The final constitution of any writing group (including designation of first and senior authors) will be on the advice of the Executive Committee and at the discretion of the study chairs.

Participating clinicians will be acknowledged in any publication. The guidelines of the International Committee of Medical Journal Editors will be followed throughout. Recruitment of patients into the trial will always be acknowledged but is not sufficient for co-author status. The involvement of all collaborative groups will be acknowledged but these groups will not have editorial input into any manuscript, except at the discretion of the study chairs. Funding bodies will be acknowledged in all manuscripts, publications and presentations. Any presentations and publications relating to the trial must be authorised by the Executive Committee.

Authorship of any secondary publications e.g. those relating to sub-studies, will reflect the intellectual and time input into these studies. The ICMJE guidelines will pertain. No investigator may present or attempt to publish data relating to the InPACT trial without prior permission from the Executive Committee.

23. ASSOCIATED STUDIES

23.1. Translational study

An extensive translational programme (optional by country) has been proposed, funding for which will require a separate grant submission. Initial proposed areas of research are:

1. PREVALENCE AND PROGNOSTIC IMPORTANCE OF HPV INFECTION.
HPV infection in oropharyngeal cancer is associated with improved outcomes. Data from the Netherlands suggests that HPV may be associated with better prognosis in node-negative penis cancer. The aim will be for collection of all tumour samples, with centralised testing for HPV DNA and for p16 protein expression. (Prof D Berney)

2. CHANGES IN SIGNALLING PATHWAYS AND THEIR RELATIONSHIP TO HPV
Constitutive activation of signalling pathways will be examined with reference to the HER family and the PTEN/akt/mTOR pathway. (Prof D Berney)

Prospective consent will be sought for access to formalin fixed paraffin embedded (FFPE) tissue blocks routinely obtained at diagnosis. FFPE blocks will be requested retrospectively from sites for analysis at a later date once appropriate funding has been secured.

For UK sites, these blocks will be held in an HTA approved facility by Prof DM Berney while translational hypotheses are developed, and tissue taken for tissue micro array and molecular tests.

For UK sites, blocks should be sent to:
Prof Dan Berney
Orchid Research Tissue Bank,
Centre for Molecular Oncology,
Barts Cancer Institute,

John Vane Science centre,
Charterhouse square,
London,
EC1M 6BQ

Eight 1mm cores of tissue will be taken from each case to ensure sufficient material for future translational studies. The information will be held in the HTA accredited facilities at St Bartholomew's hospital, in locked cabinets, and on password protected computers. Tissue will also be held in locked appropriate facilities. Cores will be taken from 'waste' tissue after pathological diagnosis. Accompanying documentation should include the pathology report and consent documentation.

The custodian of these samples will be The Institute of Cancer Research. Access requests should be directed to inpact-icrctsu@icr.ac.uk.

23.2. Quality of Life (QL) study

This is a complex trial and is likely to have long-term functional effects that will have an impact on quality of life (QL). A literature review of QOL research shows that penile cancer has negative effects on wellbeing in up to 40% of patients, with approximately 50% reporting psychological dysfunction [25]. Given the potential functional impact of the different arms of the trial, QL will be an important factor in deciding the best treatment. QL will be measured using psychometric measures. The QL component of this trial will benefit from the experience (Branney) of Patients' Experience of Penile Cancer (PEPC), a Research for Patient Benefit study that interviewed 27 patients across the UK to create a patient information resource https://healthtalk.org/.

Taking a holistic common assessment approach, the *core* Quality of Life (QL) components assessed will be general and symptom-specific.

1) General QL will be assessed using the European Organisation for Research and Treatment of Cancer (EORTC) Quality of Life QLQ-C30 [26], a self-administered multi-dimensional measure with 30 items.

2) The symptom-specific symptom is lymphodema. Lymphodema-QL will be measured using a 20 item scale [27] with domains covering heaviness, swelling-general, swelling-limb, infection, aching, numbness, physical functioning and a total symptom score.

All participants in the trial will be offered participation in the QL study. Questionnaires would be completed in the clinic at randomisation, post-neoadjuvant treatment (where applicable), post-ILND and at 12, 18, 24 and 36 months from the start of treatment.

24. REFERENCES

1. Cancer Research UK, UK Cancer Incidence 2011 and Mortality 2012 Summary - Rates, September 2014.
2. American Cancer Society: Cancer Facts and Figures 2014. Atlanta, Ga: American Cancer Society, 2014. Available online. Last accessed 29th September 2014.
3. Ravi R. Correlation between the extent of nodal involvement and survival following groin dissection for carcinoma of the penis. British journal of urology. 1993 Nov;72(5 Pt 2):817-9. PubMed PMID: 8281416.
4. Srinivas, V, Morse MJ, Herr HW, Sogani PC, Whitmore WF Jr. Penile cancer: relation of extent of nodal metastasis to survival. Journal of Urology, 1987. 137(5): p. 880-2. PMID:3573181.
5. Leijte JA, Kerst JM, Bais E, Antonini N, Horenblas S. Neoadjuvant chemotherapy in advanced penile carcinoma. European urology. 2007 Aug;52(2):488-94. PubMed PMID: 17316964.
6. Pagliaro LC, Williams DL, Daliani D, Williams MB, Osai W, Kincaid M, Wen S, Thall PF, Pettaway CA. Neoadjuvant paclitaxel, ifosfamide, and cisplatin chemotherapy for metastatic penile cancer: a phase II study. Journal of clinical oncology. 2010 Aug 20;28(24):3851-7. PubMed PMID: 20625118. Pubmed Central PMCID: 2940402.
7. Lont AP, Kroon BK, Gallee MP, van Tinteren H, Moonen LM, Horenblas S. Pelvic lymph node dissection for penile carcinoma: extent of inguinal lymph node involvement as an indicator for pelvic lymph node involvement and survival. The Journal of urology. 2007 Mar;177(3):947-52; discussion 52. PubMed PMID: 17296384.
8. Franks KN, Kancherla K, Sethugavalar B, Whelan P, Eardley I, Kiltie AE. Radiotherapy for node positive penile cancer: experience of the Leeds teaching hospitals. The Journal of urology. 2011 Aug;186(2):524-9. PubMed PMID: 21700296.
9. Bahl A, Nicholson S, Harland S, Chester J, Barber J, Pickering L, Cruickshank C, Burnett S, Waters R, Hall E, Grp TTM. A Phase II Trial of Docetaxel, Cisplatin, 5-Fluorouracil (TPF) in Locally Advanced and Metastatic Carcinoma of the Penis (CRUK/09/001). Eur J Cancer. 2011 Sep;47:S507-S. PubMed PMID: WOS:000295752801751.
10. Stankiewicz E, Prowse DM, Ktori E, Cuzick J, Ambroisine L, Zhang XX, Kudahetti S, Watkin N, Corbishley C, Berney DM. The retinoblastoma protein/p16INK4A pathway but not p53 is disrupted by human papillomavirus in penile squamous cell carcinoma. Histopathology. 2011 Feb;58(3):433-9. PubMed PMID: WOS:000287813200011
11. Tornesello ML, Duraturo ML, Losito S, Botti G, Pilotti S, Stefanon B, De Palo G, Gallo A, Buonaguro L, Buonaguro FM. Human papillomavirus genotypes and HPV16 variants in penile carcinoma. Int J Cancer. 2008 Jan 1;122(1):132-7. PubMed PMID: WOS:000251193700017
12. Stankiewicz E, Prowse DM, Ng M, Cuzick J, Mesher D, Hiscock F, Lu YJ, Watkin N, Corbishley C, Lam W, Berney DM. Alternative HER/PTEN/Akt Pathway Activation in HPV Positive and Negative Penile Carcinomas. Plos One. 2011 Mar 2;6(3). PubMed PMID: WOS:000287933300031.
13. Graafland NM, Teertstra HJ, Besnard APE, van Boven HH and Horenblas S. Identification of High Risk Pathological Node Positive Penile Carcinoma: Value of Preoperative Computerized Tomography Imaging. The Journal of urology. 2011 Mar;185(3):881-887. PubMed PMID: 21239014.
14. Rosevear HM, Williams H, Collins M, Lightfoot AJ, Coleman T and Brown JA, Utility of 8F-FDG PET/CT in identifying penile squamous cell carcinoma metastatic lymph nodes. Urol 2012; 30: 723-6
15. Sadeghi R, Gholami H, Zakavi SR, Kakhki VRD and Horenblas S, Accuracy of 18F-FDG PET/CT for diagnosing inguinal lymph node involvement in penile squamous cell carcinoma: systematic review and meta-analysis of the literature. Clin Nucl Med 2012; 37: 436-41
16. Jakobsen JK, Frahm Nielsen T, Ipsen P, et al., DaPeCa-7: comparative assessment of fluorodeoxyglucose positron emission tomography/computed tomography (CT) and conventional diagnostic CT in diagnosis of lymph node metastases, distant metastases and incidental findings in patients with invasive penile cancer. BJU International
17. Bandini M, Albersen M, Chipollini J, et al., Optimising the selection of candidates for neoadjuvant chemotherapy amongst patients with node-positive penile squamous cell carcinoma. BJU International 2020; 125: 867-75
18. Pizzocaro G, Algaba F, Horenblas S, Solsona E, Tana S, Van Der Poel H, Watkin NA, European Association of Urology Guidelines Group on Penile C. EAU penile cancer guidelines 2009. European urology. 2010 Jun;57(6):1002-12. PubMed PMID: 20163910.
19. Bevan-Thomas R, Slaton JW, Pettaway CA. Contemporary morbidity from lymphadenectomy for penile squamous cell carcinoma: The M. D. Anderson Cancer Center experience. J Urol 2002;167:1638-1642.
20. Leijte JAP, Hughes B, Graafland NM et al: Two-center evaluation of dynamic sentinel node biopsy for squamous cell carcinoma of the penis. J Clin Oncol 2009; 27: 3325.
21. Lilford RJ, Thornton JG, Braunholtz D. Clinical trials and rare diseases: a way out of a conundrum. BMJ 1995 Dec 16;311(7020):1621-5. PubMed PMID: 8555809. Pubmed Central PMCID: 2551510.
22. Naumann CM, Alkatout I, Al-Najar A, Korda JB, Hegele A, Bolenz C, Ziegler H, Klöppel G, Juenemann KP, van der Horst C. Lymph-node metastases in intermediate-risk squamous cell carcinoma of the penis. BJU Int. 2008 Nov;102(9):1102-6. PMID: 18489528.
23. Lont AP, Horenblas S, Tanis PJ, Gallee MP, van Tinteren H, Nieweg OE. Management of clinically node negative penile carcinoma: improved survival after the introduction of dynamic sentinel node biopsy. J Urol. 2003 Sep;170(3):783-6). PMID: 12913697.
24. Collett D. Modelling Survival Data in Medical Research, 2nd Edition, Chapman & Hall/CRC

25. Maddineni SB, Lau MM, Sangar VK. Identifying the needs of penile cancer sufferers: a systematic review of the quality of life, psychosexual and psychosocial literature in penile cancer. BMC urology. 2009;9:8.
26. Fayers P, Bottomley A. Quality of life research within the EORTC - the EORTC QLQ-C30. Eur J Cancer. 2002;38, Supplement 4(0):125-33.
27. Keeley V, Crooks S, Locke J, Veigas D, Riches K, Hilliam R. A quality of life measure for limb lymphoedema (LYMQOL). Journal of Lymphoedema. 2012;5(1)
28. Friedlin B, Korn E L and Gray R. A general inefficacy interim monitoring rule for randomized clinical trials. Clinical Trials 2010; 7: 197-208

APPENDIX 1 - PRE HYDRATION AND POST HYDRATION EXAMPLES FOR TIP REGIMEN.

Pre-hydration of 1 litre sodium chloride 0.9% before each dose of cisplatin is recommended. Details of pre-hydration may be according to local practice, an example is given below:

e.g. on day 1, Paclitaxel 175 mg/m^2 in 500 ml sodium chloride 0.9% over 3 hours may be given concurrent with sodium chloride 0.9% 500 ml over 3 hours, to equal 1 litre. Addition of co-medications (mannitol, magnesium sulfate, potassium chloride) to pre-hydration IV fluid will be at the discretion of the clinician. The recommended duration of pre-hydration on days 2-5 is 2 hours.

Post-hydration may be according to local practice, an example is given below:

e.g. Addition of co-medications (mannitol, magnesium sulfate, potassium chloride) will be at the discretion of the clinician. The recommended duration of post-hydration on day 1 is one hour, and on days 2-5 it will run concurrent with ifosfamide.

APPENDIX 2 - ECOG PERFORMANCE STATUS

ECOG PERFORMANCE STATUS*	
**Grade**	**ECOG**
0	Fully active, able to carry on all pre-disease performance without restriction
1	Restricted in physically strenuous activity but ambulatory and able to carry out work of a light or sedentary nature, e.g., light house work, office work
2	Ambulatory and capable of all selfcare but unable to carry out any work activities. Up and about more than 50% of waking hours
3	Capable of only limited selfcare, confined to bed or chair more than 50% of waking hours
4	Completely disabled. Cannot carry on any selfcare. Totally confined to bed or chair
5	Dead

* As published in Am. J. Clin. Oncol.:
Oken, M.M., Creech, R.H., Tormey, D.C., Horton, J., Davis, T.E., McFadden, E.T., Carbone, P.P.: Toxicity And Response Criteria Of The Eastern Cooperative Oncology Group. Am J Clin Oncol 5:649-655, 1982.

Eastern Cooperative Oncology Group, Robert Comis M.D., Group Chair.

APPENDIX 3 - CREATININE CLEARANCE CALCULATION

Cockcroft & Gault calculation
Men:

$$\text{Creatinine Clearance } /\text{ml}/\text{min} = \frac{(140\text{-age}) \times \text{mass(kg)} \times 1.23}{\text{Serum Creatinine } (\mu\text{mol}/\text{L})}$$

Modification of Diet in Renal Disease (MDRD) formula
Where local chemistry laboratories are not providing MDRD calculation as a routine result, an online eGFR calculator should be used, examples to be found at the following websites:

- www.nephron.com/MDRD_GFR.cgi
- www.kidney.org/professionals/KDOQI/gfr_calculator.cfm
- www.hdcn.com/calc.htm

APPENDIX 4 - QUALITY OF LIFE STUDY

Background

This is a complex trial and as participants will have a locally advanced tumour treatment is likely to have long-term functional effects that will impact quality of life (QL). A literature review of QL research shows that penile cancer has negative effects on well-being in up to 40% of patients, with approximately 50% reporting significant psychological dysfunction (1). Given the potential functional impact of the different arms of the trial, QL will be an important factor in deciding the best treatment. QL will be measured using psychometric measures. The QL component of this trial will benefit from the experience (Branney) of Patients' Experience of Penile Cancer (PEPC), a Research for Patient Benefit study that interviewed 27 patients across the UK to create a patient information resource https://healthtalk.org/.

Hypothesis

There will be a significant difference in quality of life between the treatment arms in InPACT.

Quality of life measures

Taking a holistic common assessment approach(2), the *core* Quality of Life (QL) components assessed will general, condition- and symptom-specific.

General QL will be assessed using the European Organisation for Research and Treatment of Cancer (EORTC) Quality of Life QLQ-C30), a self-administered multi-dimensional measure. A multi-country initiative, the QLQ-C30 was developed and validated across a wide range of clinical trials of cancer treatments to provide a simple descriptive QL profile. Answered using a Likert scale, the QLQ-C30 has 28 items about frequency (from 'not at all' to 'very much') and 2 general items about health and quality of life ('very poor' to 'excellent') covering five functional (physical, role, emotional, cognitive and social), three symptom (fatigue, nausea and vomiting and pain) and global health domains. There are also single items covering dyspnoea, insomnia, appetite loss, constipation, diarrhoea and financial difficulties.

The symptom-specific symptom is lymphodema. Lymphodema-QL will be measured by adapting the Gynaecologic Cancer Lymphedema Questionnaire (GCLQ) (5). The GLCQ is a 20 item scale with domains covering heaviness, swelling-general, swelling-limb, infection, aching, numbness, physical functioning and a total symptom score. While the GLCQ was developed with gynaecologic cancer patients only one of the 20 items is sex-specific. The GLCQ could therefore be easily adapted for penile cancer patients while providing data comparable to other patients with lymphedema in the groin.

Study design

Patients are eligible for the QL assessment in this study if they fulfil the eligibility criteria and complete the baseline QL questionnaires before randomisation. Participants will be informed in the patient information sheet that they will have their QL assessment regularly while involved in this trial. QL will be an exploratory endpoint in the main trial.

Patients will be asked to complete QL questionnaires within 7 days prior to randomisation. Patients will be asked to fill out the questionnaires as completely and accurately as possible. The average time to complete the entire questionnaire is 5 minutes. All QL booklets will be administered by the centre at time points relative from the start of treatment: post-neoadjuvant treatment (where applicable), post-ILND and at 12, 18, 24 and 36 months from the start of treatment. This will total up to 7 QL assessments per participant. The target timeframe for completion of follow up questionnaires will be +/- two weeks of the scheduled follow-up assessment.

Compliance
Missing data may hamper assessment of QL in clinical trials. This may be because centres do not collect the questionnaires at the appropriate time (unit non-response), or because patients may miss questions within the questionnaires (item non-response). The latter problem occurs less than 2% on average with the QLQ-C30 instrument and should not be a problem. The former problem is particularly important if patients have advanced cancer and low performance scores. It may be minimised by ensuring that participating centres are properly informed and motivated about QL assessment. During the study, compliance with completing QL questionnaires will be monitored.

Further QL related research is planned, subject to successful funding applications. This will be optional for sites and patients. Three projects are in development:

A) Inclusion of additional questionnaires complementing the "core" QL assessments described above in a 'holistic common assessments' approach through psychometric assessment of two further QL domains:*(2)*.
Social & Occupational Well-Being: The Social Difficulties Inventory (6) is a 21-item instrument developed for use in cancer care. It provides an overall score with clinical cut-offs and includes four subscales covering everyday living, money matters, self and others, and an undefined scale including sexual matters.
Spiritual Well-Being: The Spirituality Well-Being Single Analogue Self Assessment (SWB-LASA)(7) is a single-item instrument developed in cancer care that provides valuable information and is strongly associated with multiple item measures (8).

B) Interviews: While psychometric measures provide a means of measuring experience across participants to compare treatments, they lack information to explain the scores. Indeed, a review of QOL research on penile cancer argues that we should be using qualitative methods in study trials so that we can better understand patients' experiences of treatment (9). Semi-structured audio or video recorded interviews (depending on the participant's preference) will be conducted by an experienced qualitative researcher with a sub-sample of participants. These will explore participants' quality of life during their recovery to help explain the specific issues that concern them and how they adapt. The interview schedule will map onto the QL domains covered in the psychometric measures, to explore their social and occupational, psychological and spiritual well-being and relate it to the participant's background and physical health.

C) Development of a condition-specific QL measure: The psychometric measures used in part 1 of the QL measure issues 'generic' to cancer patients, which means that we can make comparisons across conditions. Nevertheless, condition-specific measures are necessary to be able to identify those aspects unique to the tumour site. As far as we are aware, no such measure exists for penile cancer. A rare condition is unlikely to receive the support necessary to develop and pilot a condition-specific QL measure. A penile-cancer QL measure would be extremely useful for use in clinical trials when treatments are potentially so detrimental to urological functioning. Additionally, a QL measure could be used as a screening tool to help MDTs identify if and when patients need referring for social and psychological support. In this trial, we would identify and pilot a penile-cancer QL measure.

References:

1. Maddineni S, Lau M, Sangar V. Identifying the needs of penile cancer sufferers: A systematic review of the quality of life, psychosexual and psychosocial literature in penile cancer. BMC Urology. 2009;9(1):8. PubMed PMID: doi:10.1186/1471-2490-9-8.
2. Richardson A, Tebbit P, Sitzia J, Brown V. Holistic common assessment of supportive and palliative care needs for adults with cancer. London: Department of Health; 2007.
3. Rabin R, Oemar M, Oppe M, Janssen B, MHerdman M, EuroQol Group Executive Office. EQ-5D-5L user guide. Rotterdam, The Netherlands: EuroQol Group; 2011.
4. Rosen RC, Riley A, Wagner G, Osterloh IH, Kirkpatrick J, Mishra A. The international index of erectile function (IIEF): a multidimensional scale for assessment of erectile dysfunction. Urology. 1997;49(6):822-30.
5. Carter J, Raviv L, Appollo K, Baser RE, Iasonos A, Barakat RR. A pilot study using the Gynecologic Cancer Lymphedema Questionnaire (GCLQ) as a clinical care tool to identify lower extremity lymphedema in gynecologic cancer survivors. Gynecologic Oncology. 2010 5//;117(2):317-23.
6. Wright P, Smith AB, Keding A, Velikova G. The Social Difficulties Inventory (SDI): development of subscales and scoring guidance for staff. Psycho-Oncology. 2011;20(1):36-43.
7. Locke DEC, Decker PA, Sloan JA, Brown PD, Malec JF, Clark MM, et al. Validation of Single-Item Linear Analog Scale Assessment of Quality of Life in Neuro-Oncology Patients. Journal of Pain and Symptom Management. 2007;34(6):628-38.
8. Johnson ME, Piderman KM, Sloan JA, Huschka M, Atherton PJ, Hanson JM, et al. Measuring spiritual quality of life in patients with cancer. Journal of Supportive Oncology. 2007;5(9):437-42. PubMed PMID: 2009709845.
9. Branney PE, Raine G, Witty K, Braybrook D, Bullen K, White AK, et al. Quality of life after treatment for penile cancer: a systematic research review of qualitative research of patients' experiences. Journal of Men's Health. [in press - to update]

APPENDIX 5 – Chemotherapy dose levels and use of GCSF

The following table details the chemotherapy dose levels with and without the use of G-CSF and dose modifications / reductions in the presence of toxicity. Please refer to the conversion table to ensure delivery of the correct daily dose and refer to section 9.3.1 for the correct regimen. Table 1 below should be used in conjunction with the dose modification table (table 2) of this appendix.

Table 1.

Drug	Full / modified dose	Total Cycle Dose (mg/m^2) as detailed in appendix 5	Daily Dose conversion (mg/m^2)	
			**Outpatient**	**Inpatient**
paclitaxel	FULL	175 mg/m^2	175 mg/m^2<br>*(given day 1)*	175 mg/m^2<br>*(given day 1)*
	MODIFIED / REDUCED	140 mg/m^2	140 mg/m^2<br>*(given day 1)*	140 mg/m^2<br>(given day 1)
Ifosfamide	FULL	3600 mg/m^2	900 mg/m^2<br>*(given days 2-5)*	1200 mg/m^2<br>*(given days 1-3)*
	MODIFIED / REDUCED	2880 mg/m^2	720 mg/m^2<br>*(given days 2-5)*	960 mg/m^2<br>*(given days 1-3)*
Cisplatin	FULL	75 mg/m^2	15 mg/m^2<br>*(given days 1-5)*	25 mg/m^2<br>*(given days 1-3)*
	MODIFIED / REDUCED	60 mg/m^2	12 mg/m^2<br>*(given days 1-5)*	20 mg/m^2<br>*(given days 1-3)*

All patients should receive the full dose of the TIP regimen at cycle 1. This table details the dose modifications required from cycle 2 onwards based on the total dose mg/m^2 given at the previous cycle +/- G=CSF.

Table 2.

		**Previous cycle chemotherapy dose levels and use of G-CSF**			
Type of Toxicity	Grade	Paclitaxel 175mg/m^2<br>Ifosfamide 3,600mg/m^2<br>Cisplatin 75mg/m^2	Paclitaxel 175mg/m^2<br>Ifosfamide 3,600mg/m^2<br>Cisplatin 75mg/m^2<br>given with G-CSF	Paclitaxel 140mg/m^2<br>Ifosfamide 2,880mg/m^2<br>Cisplatin 75mg/m^2	Paclitaxel 140mg/m^2<br>Ifosfamide 2,880mg/m^2<br>Cisplatin 75mg/m^2<br>given with G-CSF
	CTC v4.0	**Dose modification and management for next cycle of chemotherapy**			
Haematological					
Neutropenia	**1**<br><LLN - 1500/m^3;<br><LLN - 1.5 x 10^9/L	Full doses	Full doses<br>give prophylactic G-CSF	Paclitaxel 140mg/m^2<br>Ifosfamide 2,880mg/m^2<br>Cisplatin 75mg/m^2	Paclitaxel 140mg/m^2<br>Ifosfamide 2,880mg/m^2<br>Cisplatin 75mg/m^2<br>give prophylactic G-CSF
	**2**<br><1500 - 1000/m^3;<br><1.5 - 1.0 x 10^9/L	Full doses<br>delay is not required but is at discretion of treating clinician	Full doses<br>give prophylactic G-CSF<br>delay is not required but is at discretion of treating clinician	Paclitaxel 140mg/m^2<br>Ifosfamide 2,880mg/m^2<br>Cisplatin 75mg/m^2<br>delay is not required but is at discretion of treating clinician	Paclitaxel 140mg/m^2<br>Ifosfamide 2,880mg/m^2<br>Cisplatin 75mg/m^2<br>give prophylactic G-CSF<br>delay is not required but is at discretion of treating clinician
	**3**<br><1000 - 500/mm^3;<br><1.0 - 0.5 x 10^9/L	Delay until ≥ 1.0 x 10^9/L<br>give prophylactic G-CSF<br>Full doses	Delay until ≥ 1.0 x 10^9/L<br>give prophylactic G-CSF<br>Paclitaxel 140mg/m^2<br>Ifosfamide 2,880mg/m^2<br>Cisplatin 75mg/m^2	Delay until ≥ 1.0 x 10^9/L<br>give prophylactic G-CSF<br>Paclitaxel 140mg/m^2<br>Ifosfamide 2,880mg/m^2<br>Cisplatin 75mg/m^2	Stop
	**4**<br><500/mm^3;<br><0.5 x 10^9/L	Delay until ≥ 1.0 x 10^9/L<br>give prophylactic G-CSF<br>Full doses	Delay until ≥ 1.0 x 10^9/L<br>give prophylactic G-CSF<br>Paclitaxel 140mg/m^2<br>Ifosfamide 2,880mg/m^2<br>Cisplatin 75mg/m^2	Delay until ≥ 1.0 x 10^9/L<br>give prophylactic G-CSF<br>Paclitaxel 140mg/m^2<br>Ifosfamide 2,880mg/m^2<br>Cisplatin 75mg/m^2	Stop
Thrombocytopenia	**1**<br><LLN - 75,000/mm^3;<br><LLN - 75.0 x 10^9/L	delay until ≥75 x 10^9/L<br>Full doses	delay until ≥75 x 10^9/L<br>give G-CSF as per previous<br>Full doses	delay until ≥75 x 10^9/L<br>Paclitaxel 140mg/m^2<br>Ifosfamide 2,880mg/m^2<br>Cisplatin 75mg/m^2	delay until ≥ 75 x 10^9/L<br>give G-CSF as per previous<br>Paclitaxel 140mg/m^2<br>Ifosfamide 2,880mg/m^2<br>Cisplatin 75mg/m^2
	**2**<br><75,000 - 50,000/mm^3;<br><75.0 - 50.0 x 10^9/L	delay until ≥75 x 10^9/L<br>Full doses	delay until ≥75 x 10^9/L<br>give G-CSF as per previous<br>Full doses	delay until ≥75 x 10^9/L<br>Full doses	delay until ≥75 x 10^9/L<br>give G-CSF as per previous<br>Paclitaxel 140mg/m^2<br>Ifosfamide 2,880mg/m^2<br>Cisplatin 75mg/m^2

	**3**<br><br><50,000 - 25,000/mm³;<br><50.0 - 25.0 x 10^9/L	delay until ≥75 x 10^9/L<br>Paclitaxel 140mg/m²<br>Ifosfamide 2,880mg/m²<br>Cisplatin 75mg/m²	delay until ≥75 x 10^9/L<br>Paclitaxel 140mg/m²<br>Ifosfamide 2,880mg/m²<br>Cisplatin 75mg/m²	delay until ≥75 x 10^9/L<br>Paclitaxel 140mg/m²<br>Ifosfamide 2,880mg/m²<br>Cisplatin 75mg/m²	delay until ≥75 x 10^9/L<br>give G-CSF as per previous<br>*or*<br>Stop<br>Paclitaxel 140mg/m²<br>Ifosfamide 2,880mg/m²<br>Cisplatin 75mg/m²
	**4**<br><br><25,000/mm³;<br><25.0 x 10^9/L	delay until platelets >75 x 10^9/L<br>Paclitaxel 140mg/m²<br>Ifosfamide 2,880mg/m²<br>Cisplatin 75mg/m²	delay until platelets >75 x 10^9/L<br>Paclitaxel 140mg/m²<br>Ifosfamide 2,880mg/m²<br>Cisplatin 75mg/m²	delay until platelets >75 x 10^9/L<br>Paclitaxel 140mg/m²<br>Ifosfamide 2,880mg/m²<br>Cisplatin 75mg/m²	Stop
Febrile Neutropenia	**3**<br><br>ANC <1000/mm³ with a single temperature of >38.3 degrees C (101 degrees F) or a sustained temperature of >=38 degrees C (100.4 degrees F) for more than one hour.	Delay until ≥1 x 10^9/L<br>give prophylactic G-CSF<br>Full doses	Delay until ≥1 x 10^9/L<br>give prophylactic G-CSF<br>Paclitaxel 140mg/m²<br>Ifosfamide 2,880mg/m²<br>Cisplatin 75mg/m²	Delay until ≥1 x 10^9/L<br>give prophylactic G-CSF<br>Paclitaxel 140mg/m²<br>Ifosfamide 2,880mg/m²<br>Cisplatin 75mg/m²	**Stop**
	**4**<br><br>Life-threatening consequences; urgent intervention indicated.	**Stop**<br>*Or*<br>Delay until ≥1 x 10^9/L<br>give prophylactic G-CSF<br>Paclitaxel 140mg/m²<br>Ifosfamide 2,880mg/m²<br>Cisplatin 75mg/m²	**Stop**<br>*Or*<br>Delay until ≥1 x 10^9/L<br>give prophylactic G-CSF<br>Paclitaxel 140mg/m²<br>Ifosfamide 2,880mg/m²<br>Cisplatin 75mg/m²	**Stop**	**Stop**
Non-haematological					
Cutaneous	**1**<br><br>Urticarial lesions covering <10% BSA; topical intervention indicated	Full doses		Paclitaxel 140mg/m²<br>Ifosfamide 2,880mg/m²<br>Cisplatin 75mg/m²	
	**2**<br><br>Urticarial lesions covering 10 - 30% BSA; oral intervention indicated				
	**3**<br><br>Urticarial lesions covering >30% BSA; IV intervention Indicated	Paclitaxel 140mg/m²<br>Ifosfamide 2,880mg/m²<br>Cisplatin 75mg/m²		Stop	
Peripheral Neuropathy	**1**<br><br>Asymptomatic; loss of deep	Full doses		Paclitaxel 140mg/m²<br>Ifosfamide 2,880mg/m²<br>Cisplatin 75mg/m²	

(as assessed prior to next scheduled cycle of chemotherapy)	tendon reflexes or paresthesia		
	**2**<br>Moderate symptoms; limiting instrumental ADL	Delay<br>OR<br>Paclitaxel 140mg/m^2<br>Ifosfamide 3,600mg/m^2<br>Cisplatin 60mg/m^2	Delay<br>OR<br>Paclitaxel 140mg/m^2<br>Ifosfamide 2,880mg/m^2<br>Cisplatin 60mg/m^2
	**3**<br>Severe symptoms; limiting self care ADL	Withhold until resolution to grade 1 or better;<br>Paclitaxel 140mg/m2<br>Ifosfamide 3,600mg/m2<br>Cisplatin 60mg/m2	Withhold until resolution to grade 1 or better
	**4**<br>Life-threatening consequences; urgent intervention indicated	Stop	Stop
Liver enzymes	1	Full doses	Paclitaxel 140mg/m^2<br>Ifosfamide 2,880mg/m^2<br>Cisplatin 75mg/m^2<br>Delay for maximum of 2 weeks
	2	Delay for maximum of 2 weeks<br>Paclitaxel 140mg/m^2<br>Ifosfamide 3,600mg/m^2<br>Cisplatin 75mg/m^2	Delay for maximum of 2 weeks<br>Paclitaxel 140mg/m^2<br>Ifosfamide 2,880mg/m^2<br>Cisplatin 75mg/m^2
	3	**Stop**	**Stop**
	4	**Stop**	**Stop**
GFR	see text	Delay for maximum of 2 weeks if GFR < 50ml/min – one dose reduction is permitted for renal toxicity:<br>Paclitaxel 175mg/m^2<br>Ifosfamide 2,880mg/m^2<br>Cisplatin 60mg/m^2	
	3	**Stop**	**Stop**
	4	**Stop**	**Stop**

APPENDIX 6 - Modifed Clavien- Dindo Classification*

Dindo et al. Classification of surgical complications: a new proposal with evaluation in a cohort of 6336 patients and results of a survey. Ann Surg (2004) vol. 240 (2) pp. 205-13

TABLE 1. Classification of Surgical Complications

Grade	Definition
Grade I	Any deviation from the normal postoperative course without the need for pharmacological treatment or surgical, endoscopic, and radiological interventions<br>Allowed therapeutic regimens are: drugs as antiemetics, antipyretics, analgetics, diuretics, electrolytes, and physiotherapy. This grade also includes wound infections opened at the bedside
Grade II	Requiring pharmacological treatment with drugs other than such allowed for grade I complications<br>Blood transfusions and total parenteral nutrition are also included
Grade III	Requiring surgical, endoscopic or radiological intervention
Grade IIIa	Intervention not under general anesthesia
Grade IIIb	Intervention under general anesthesia
Grade IV	Life-threatening complication (including CNS complications)* requiring IC/ICU management
Grade IVa	Single organ dysfunction (including dialysis)
Grade IVb	Multiorgan dysfunction
Grade V	Death of a patient
Suffix "d"	If the patient suffers from a complication at the time of discharge (see examples in Table 2), the suffix "d" (for "disability") is added to the respective grade of complication. This label indicates the need for a follow-up to fully evaluate the complication.

*Brain hemorrhage, ischemic stroke, subarrachnoidal bleeding, but excluding transient ischemic attacks.
CNS, central nervous system; IC, intermediate care; ICU, intensive care unit.

TABLE 2. Clinical Examples of Complication Grades

Grades	Organ System	Examples
Grade I	Cardiac	Atrial fibrillation converting after correction of K^+-level
	Respiratory	Atelectasis requiring physiotherapy
	Neurological	Transient confusion not requiring therapy
	Gastrointestinal	Noninfectious diarrhea
	Renal	Transient elevation of serum creatinine
	Other	Wound infection treated by opening of the wound at the bedside
Grade II	Cardiac	Tachyarrhythmia requiring β-receptor antagonists for heart rate control
	Respiratory	Pneumonia treated with antibiotics on the ward
	Neurological	TIA requiring treatment with anticoagulants
	Gastrointestinal	Infectious diarrhea requiring antibiotics
	Renal	Urinary tract infection requiring antibiotics
	Other	Same as for I but followed by treatment with antibiotics because of additional phlegmonous infection
Grade IIIa	Cardiac	Bradyarrhythmia requiring pacemaker implantation in local anesthesia
	Neurological	See grade IV
	Gastrointestinal	Biloma after liver resection requiring percutaneous drainage
	Renal	Stenosis of the ureter after kidney transplantation treated by stenting
	Other	Closure of dehiscent noninfected wound in the OR under local anesthesia
Grade IIIb	Cardiac	Cardiac temponade after thoracic surgery requiring fenestration
	Respiratory	Bronchopleural fistulas after thoracic surgery requiring surgical closure
	Neurological	See grade IV
	Gastrointestinal	Anastomotic leakage after descendorectostomy requiring relaparotomy
	Renal	Stenosis of the ureter after kidney transplantation treated by surgery
	Other	Wound infection leading to eventration of small bowel
Grade IVa	Cardiac	Heart failure leading to low-output syndrome
	Respiratory	Lung failure requiring intubation
	Neurological	Ischemic stroke/brain hemorrhage
	Gastrointestinal	Necrotizing pancreatitis
	Renal	Renal insufficiency requiring dialysis
Grade IVb	Cardiac	Same as for IVa but in combination with renal failure
	Respiratory	Same as for IVa but in combination with renal failure
	Gastrointestinal	Same as for IVa but in combination with hemodynamic instability
	Neurological	Ischemic stroke/brain hemorrhage with respiratory failure
	Renal	Same as for IVa but in combination with hemodynamic instability
Suffix "d"	Cardiac	Cardiac insufficiency after myocardial infarction (IVa–d)
	Respiratory	Dyspnea after pneumonectomy for severe bleeding after chest tube placement (IIIb–d)
	Gastrointestinal	Residual fecal incontinence after abscess following descendorectostomy with surgical evacuation. (IIIb–d)
	Neurological	Stroke with sensorimotor hemisyndrome (IVa–d)
	Renal	Residual renal insufficiency after sepsis with multiorgan dysfunction (IVb–d)
	Other	Hoarseness after thyroid surgery (I–d)

TIA, transient ischemic attack; OR, operating room.

APPENDIX 7 - Response Evaluation Criteria in Solid Tumours (RECIST v 1.1) Quick Reference

Full guidance document available at: https://project.eortc.org/recist/wp-content/uploads/sites/4/2015/03/RECISTGuidelines.pdf

Definitions

At baseline, tumour lesions/lymph nodes are categorised as measurable or non measureable:

Measurable tumour lesions:

Must be accurately measure in at least one dimension (longest diameter in the plane of measurement should be recorded) with a minimum size of:

- 10mm by CT scan (CT scan slice thickness no great that 5mm).
- 10mm caliper measurement by clinical exam (lesions which can't be accurately measured with calipers should be recorded as non-measureable).
- 20mm by chest X-ray.

Malignant lymph nodes:

To be considered pathologically enlarged and measurable, a lymph node must be ≥15mm in short axis when assessed by CT scan (CT scan slice thickness recommended to be no greater than 5 mm). At baseline and in follow-up, only the short axis will be measured and followed.

Non measurable:

All other lesions, including small lesions (longest diameter <10mm or pathological lymph nodes with P10 to <15mm short axis) as well as truly non-measurable lesions. Lesions considered truly non-measurable include: leptomeningeal disease, ascites, pleural or pericardial effusion, inflammatory breast disease, lymphangitic involvement of skin or lung, abdominal masses/abdominal organomegaly identified by physical exam that is not measurable by reproducible imaging techniques.

Guidance on bone lesions, cystic lesions, and lesions previously treated with local therapy is contained within the full RECIST guidance document.

Methods of Measurement

The same method of assessment and the same technique should be used to characterise each identified and reported lesion at baseline and during follow-up. Imaging based evaluation should always be done rather than clinical examination unless the lesion(s) being followed cannot be imaged but are assessable by clinical exam. Further guidance is given in the full RECIST guidance document.

Response Criteria

	**Evaluation of target lesions**
* Complete Response (CR):	Disappearance of all target lesions. Any pathological lymph nodes (whether target or non-target) must have reduction in short axis to <10mm.
* Partial Response (PR):	At least a 30% decrease in the sum of diameters of target lesions, taking as reference the baseline diameters.
* Progressive Disease (PD):	At least a 20% increase in the sum of diameters of target lesions, taking as reference the smallest sum on study (this includes the baseline sum if that is the smallest on study). In addition to the relative increase of 20%, the sum must also demonstrate an absolute increase of at least 5mm. Note: the appearance of one or more lesions is also considered progression.
* Stable Disease (SD):	Neither sufficient shrinkage to qualify for PR nor sufficient increase to qualify for PD, taking as reference the smallest sum diameters

	while on study.

	**Evaluation of non-target lesions**
* Complete Response (CR):	Disappearance of all non-target lesions and normalisation of tumour marker level. All lymph nodes must be non-pathological in size (<10mm short axis).
*Incomplete Response / Stable Disease (SD):	Persistence of one or more non-target lesion(s) or/and maintenance of tumour marker level above the normal limits.
* Progressive Disease (PD):	Unequivocal progression of existing non-target lesions (1). Note: The appearance of one or more new lesions is also considered progression.

(1) Although a clear progression of "non-target" lesions only is exceptional, in such circumstances, the opinion of the treating physician should prevail and the progression status should be confirmed later on by the review panel (or study chair).

Evaluation of best overall response

The best overall response is the best response recorded from the start of the treatment until disease progression/recurrence (taking as reference for PD the smallest measurements recorded since the treatment started). In general, the patient's best response assignment will depend on the achievement of both measurement and confirmation criteria.

The table below provides a summary of the overall response calculation at each time point.

**Target lesions**	**Non-Target lesions**	**New Lesions**	**Overall response**
CR	CR	No	CR
CR	Incomplete response/SD	No	PR
CR	Not-evaluated*	No	PR
PR	Non-PD or not all evaluated	No	PR
SD	Non-PD or not all evaluated	No	SD
Not all evaluated	Non-PD	No	Not evaluable
PD	Any	Yes or No	PD
Any	PD	Yes or No	PD
Any	Any	Yes	PD

** When no imaging/measurement is done at all at a particular time point, the patient is not evaluable (NE) at that time point. If only a subset of lesion measurements are made at an assessment, usually the case is also considered NE at that time point, unless a convincing argument can be made that the contribution of the individual missing lesion(s) would not change the assigned time point response.*

APPENDIX 8 – Expected serious adverse events for radiotherapy or surgery alone

Expected events from standard surgery (ILND or PLND) alone:

- Haematoma.
- Lymphatic leak or seroma.
- Infection.
- Necrosis of wound edges.
- Lymphoedema.
- Haemorrhage

These risks are not expected to be greater in the InPACT trial population than in the non-trial population undergoing therapeutic ILND as part of routine care.

Expected events from neoadjuvant chemoradiotherapy alone (only if no concurrent cisplatin administered only):

- Exposure of / toxicity to skin, bladder, bowels, spine, femoral heads, and testicles/scrotum.
- Constipation and/or diarrhoea.

Expected events from adjuvant chemoradiotherapy alone (only if no concurrent cisplatin administered only):

- Exposure of / toxicity to skin, bladder, bowels, spine, femoral heads, and testicles/scrotum.
- Constipation and/or diarrhoea.
- Lymphoedema which may be worse than that seen with surgery alone.

Glossary

AE	Adverse Event
ALT	Alanine Aminotransferase
ANC	Absolute Neutrophil Count
AST	Aspartate Aminotransferase
AUC	Area Under Curve
CI	Chief Investigator
CIS	Carcinoma In Situ
CRF	Case Report Form
CTCAE	Common Terminology Criteria for Adverse Events
DCF	Data Capture Form
DFS	Disease Free Survival
ECOG-ACRIN	European Cooperative Oncology Group (ECOG) and the American College of Radiology Imaging Network (ACRIN)
EORTC	European Organisation for Research and Treatment of Cancer
FBC	Full Blood Count
G-CSF	Growth Colony-Stimulating Factors
GFR	Glomerular Filtration Rate
HPV	Human Papilloma Virus
HR	Hazard Ratio
IB	Investigator's Brochure
ICR	The Institute Of Cancer Research
ICR-CTSU	The Institute Of Cancer Research – Clinical Trials & Statistics Unit
IDMC	Independent Data Monitoring Committee
IEC	InPACT Executive Committee
ILND	Inguinal Lymph Node Dissection
LFT	Liver Function Test
MDT	Multi-disciplinary team
NCI	National Cancer Institute
NCRN	NIHR Clinical Research Network
NCRI	National Cancer Research Institute
NIHR	National Institute for Health Research
PI	Principal Investigator
PIS	Patient Information Sheet
PLND	Prophylactic Pelvic Lymph Node Dissection
PTEN/Akt	phosphatase and tension homolog (PTEN)/Protein kinase B (Akt) pathway
QoL/QL	Quality of Life
R&D	Research and Development
RCT	Randomised controlled trial
RTOG	Radiation Therapy Oncology Group
RTTQA	Radiotherapy Clinical Trials Quality Assurance group
SAE	Serious Adverse Event
SAR	Serious Adverse Reaction
SMPC	Summary of Product Characteristics
SUSAR	Suspected Unexpected Serious Adverse Reaction
TMG	Trial Management Group
TSC	Trial Steering Committee
U+E	Urea & Electrolytes
UKCRC	UK Clinical Research Collaboration
ULN	Upper Limit of Normal
WBC	White Blood Cell
WHO	World Health Organisation
